# Non-COVID outcomes associated with the coronavirus disease-2019 (COVID-19) pandemic effects study (COPES): A systematic review and meta-analysis

**DOI:** 10.1371/journal.pone.0269871

**Published:** 2022-06-24

**Authors:** Vincent Issac Lau, Sumeet Dhanoa, Harleen Cheema, Kimberley Lewis, Patrick Geeraert, David Lu, Benjamin Merrick, Aaron Vander Leek, Meghan Sebastianski, Brittany Kula, Dipayan Chaudhuri, Arnav Agarwal, Daniel J. Niven, Kirsten M. Fiest, Henry T. Stelfox, Danny J. Zuege, Oleksa G. Rewa, Sean M. Bagshaw

**Affiliations:** 1 Department of Critical Care Medicine, Faculty of Medicine and Dentistry, University of Alberta, and Alberta Health Services, Edmonton, Alberta, Canada; 2 Faculty of Medicine and Dentistry, University of Alberta, and Alberta Health Services, Edmonton, Alberta, Canada; 3 Department of Health Research Methods, Evidence & Impact, McMaster University, Hamilton, Ontario, Canada; 4 Division of Critical Care Medicine, Department of Medicine, McMaster University, Hamilton, Ontario, Canada; 5 Alberta Strategy for Patient-Orientated Research Knowledge Translation Platform, University of Alberta, Edmonton, Alberta, Canada; 6 Division of Infectious Disease, Department of Medicine, Faculty of Medicine and Dentistry, University of Alberta, and Alberta Health Services, Edmonton, Alberta, Canada; 7 Department of Medicine, General Internal Medicine, McMaster University, Hamilton, Ontario, Canada; 8 Department of Critical Care Medicine, Cumming School of Medicine, University of Calgary, and Alberta Health Services, Calgary, Alberta, Canada; 9 Critical Care Strategic Clinical Network, Alberta Health Services, Calgary, Alberta, Canada; 10 O’Brien Institute of Public Health, University of Calgary, Calgary, Alberta, Canada; 11 School of Public Health, University of Alberta, Edmonton, Alberta, Canada; Duta Wacana Christian University School of Medicine / Bethesda Hospital, INDONESIA

## Abstract

**Background:**

As the Coronavirus Disease-2019 (COVID-19) pandemic continues, healthcare providers struggle to manage both COVID-19 and non-COVID patients while still providing high-quality care. We conducted a systematic review/meta-analysis to describe the effects of the COVID-19 pandemic on patients with non-COVID illness and on healthcare systems compared to non-pandemic epochs.

**Methods:**

We searched Ovid MEDLINE/EMBASE/Cochrane Database of Systematic Reviews/CENTRAL/CINAHL (inception to December 31, 2020). All study types with COVID-pandemic time period (after December 31, 2019) with comparative non-pandemic time periods (prior to December 31, 2019). Data regarding study characteristics/case-mix/interventions/comparators/ outcomes (primary: mortality; secondary: morbidity/hospitalizations/disruptions-to-care. Paired reviewers conducted screening and abstraction, with conflicts resolved by discussion. Effect sizes for specific therapies were pooled using random-effects models. Risk of bias was assessed by Newcastle-Ottawa Scale, with evidence rating using GRADE methodology.

**Results:**

Of 11,581 citations, 167 studies met eligibility. Our meta-analysis showed an increased mortality of 16% during the COVID pandemic for non-COVID illness compared with 11% mortality during the pre-pandemic period (RR 1.38, 95% CI: 1.28–1.50; absolute risk difference: 5% [95% CI: 4–6%], p<0.00001, very low certainty evidence). Twenty-eight studies (17%) reported significant changes in morbidity (where 93% reported increases), while 30 studies (18%) reported no significant change (very low certainty). Thirty-nine studies (23%) reported significant changes in hospitalizations (97% reporting decreases), while 111 studies (66%) reported no significant change (very low certainty). Sixty-two studies (37%) reported significant disruptions in standards-to-care (73% reporting increases), while 62 studies (37%) reported no significant change (very low certainty).

**Conclusions:**

There was a significant increase in mortality during the COVID pandemic compared to pre-pandemic times for non-COVID illnesses. When significant changes were reported, there was increased morbidity, decreased hospitalizations and increased disruptions in standards-of-care.

**Systematic review registration:**

PROSPERO CRD42020201256 (Sept 2, 2020).

## Introduction

Severe acute respiratory syndrome coronavirus 2 (SARS-CoV-2), the virus that causes coronavirus disease-19 (COVID-19), has spread globally to over 180 countries on 6 continents with over 500 million confirmed cases of COVID-19 worldwide, and over 6 million deaths [1, 2]. The COVID-19 pandemic has contributed to widespread disruption to the delivery of non-urgent healthcare services (e.g., scheduled surgical and elective procedure postponements/cancellations, delayed and missed cancer screening) [3] to create health system capacity and prioritize acute care access for patients with COVID-19. This has been further compounded by successive waves of surging case counts with incomplete opportunity for health systems recovery in between [4].

This shift in prioritization of the health system may have unintentional and underappreciated effects on patients without COVID, including altered access to health services and/or altered models of care. The pandemic may be contributing to substantial negative consequences for patients [5, 6] along with indirect and unintended harm reduction (e.g., reduced exposure to low-value healthcare). As an illustration, during the pandemic, patients have been found to have delayed presentations to hospital for several non-COVID urgent illnesses (e.g., stroke, acute coronary syndrome, intoxications, etc.), often due to patients’ perception to strictly adhere to public health interventions and/or fearing risk of contracting COVID-19 in hospitals [7–9]. Healthcare professionals and health systems have operated under considerable strain and may have struggled to maintain usual standards-of-care for patients admitted with non-COVID illnesses, while also having adapting to meet expanded care needs for patients with COVID-19 [10]. While the collateral damage on health systems of the COVID-19 pandemic has enormous potential global public health importance, it has remained largely unquantified.

Accordingly, to focus attention on this issue, we conducted a systematic review (SR) and meta-analysis (MA) to describe the effects of the COVID-19 pandemic on non-COVID outcomes with respect to patient mortality, morbidity, acute care hospitalizations and disruptions to standards-of-care (both at the population and healthcare system levels). Our SR serves to inform health care leaders, professionals and health policy makers, who have generated and implemented policy to prioritize resources throughout the COVID-19 pandemic, of the potential widespread impact of COVID-19 on capacity to sustainably provide standards-of-care and optimize outcomes for patients presenting with illnesses unrelated to COVID-19.

## Methods

### Searches and inclusion criteria

This SR was conducted and reported in accordance with the PRISMA guidelines [11], and was registered in PROSPERO (international prospective register of systematic reviews) on September 2, 2020 (CRD42020201256). The complete PRISMA checklist is included (S1 Table).

We systematically searched Ovid MEDLINE, EMBASE, Cochrane Database of Systematic Reviews, Cochrane Controlled Trials Register (CENTRAL), and Cumulative Index to Nursing and Allied Health Literature (CINAHL) from inception 1948 to December 31, 2020. Last search was completed on Dec 31, 2020. Searches were performed by a research librarian (DKL), and were adjudicated by a second health information specialist (MS) using Peer Review Electronic Search Strategy (PRESS) criteria (S1 Appendix) [12].

We used a combination of subject headings and keywords: *mortality; morbidity; pandemic; non-pandemic time periods; outcomes; healthcare disruption; healthcare system delivery; public health policy/measures; societal/public behaviour; acute care hospitalizations; occupancy rates; economics*. We also screened reference lists of identified relevant individual studies and reviews.

### Operational definitions

Exposure and study and control time periods were defined as during the COVID-19 pandemic (December 31, 2019 to December 31, 2020) compared to non-COVID-19 pandemic time periods (December 1948 to December 31, 2019).

Mortality was evaluated at the longest time interval provided for each study, and classified as increased or decreased relative to pre-pandemic epochs.

Morbidity was defined as the state of being symptomatic or unhealthy for a disease or condition [13], and as specifically defined in the individual studies relevant to the reported base health outcome.

A “disruption to standards-of-care” was defined as any change to a delivered health service (e.g., time to presentation or arrival, cancellation or delay to timely surgery or procedure, or diagnosis and/or treatment intervention, follow-up, etc.) which had a statistically significant change during the COVID-19 pandemic period as compared to a non-COVID pandemic historical control period (e.g., same months) [3, 14].

### Eligibility criteria

Articles were considered eligible if they met the following criteria: (1) adult patients (≥18 years old); (2) randomized control trials (RCTs), observational studies and case series with control groups at any level (e.g., population level, healthcare facilities, etc.). We excluded all animal and pediatric studies. Conference abstracts and non-peer reviewed websites were excluded. We excluded case reports and case series without control groups. No language restrictions were applied.

### Study selection and data abstraction

Paired reviewers (VL, SD, HC, PG, DL, BM, AVL, MS, KL, BK, DC, AA) independently screened the titles and abstracts of identified articles. Articles deemed potentially eligible by either or both reviewers advanced to the full-text review stage, and were screened for inclusion by paired reviewers (including pilot testing against eligibility criteria). Disagreements at this stage were resolved through discussion and consultation with a third reviewer, if necessary. We used Covidence (Veritas Health Innovation, Melbourne, Australia) to manage search results, screening, and selection of studies [15]. Our data abstraction is outlined in S2 Table.

An *a priori* data abstraction tool was piloted for all reviewers and was subsequently used to collect the following data from eligible articles: study characteristics (title, author), patient group demographic/clinical data, interventions and comparators, clinical outcome data (including morbidity and mortality, acute care hospitalizations/occupancy rates and disruptions to care), and jurisdiction(s) in which the study was performed.

### Risk of bias assessment

We assessed risk of bias in observational cohort and case-control studies using the Newcastle-Ottawa Scale (NOS), examining the following domains: selection, comparability and exposure for cohort and case-control studies. Each of the criteria for the NOS scales for cohort/case-control studies are found in the footnotes [16]. Quality of the studies were based on either good (3–4 stars in selection domain and 1–2 stars in comparability domain and 2–3 stars in outcome/exposure domain), fair (2 stars in selection domain and 1–2 stars in comparability domain and 2–3 stars in outcome/exposure domain) or poor (0–1 star in selection domain or 0 stars in comparability domain or 0–1 stars in outcome/exposure domain) [16].

### Grading of recommendations assessment, development and evaluation

We used the Grading of Recommendations Assessment, Development and Evaluation (GRADE) approach to assess the following domains for each clinical outcome: individual study risk of bias, indirectness, imprecision, inconsistency and publication bias. Certainty in evidence from observational studies started as low, with RCTs starting as high. Final certainty was rated as high, moderate, low or very low [17–19].

### Data synthesis and analysis

Continuous data was presented as means and standard deviations (SD), or medians and inter-quartile ranges (IQR), and were compared (where appropriate) using a t-test or Wilcoxon rank sum test. Categorical variables and proportions were compared using the Pearson’s Chi-Square or Fischer’s exact tests as appropriate. We summarized the eligible studies in terms of point estimates or proportions, with p-values or 95% confidence intervals [CIs], if available. Significance was set at 0.05.

We performed a meta-analysis of observational studies in this SR, with RevMan (Copenhagen: The Nordic Cochrane Centre, Cochrane Collaboration 2014) version 5.4 software for the outcome of mortality. We will use the method of DerSimonian and Laird to pool effect sizes for each outcome under a random-effects model for all outcomes of interest [20]. Study weights were measured using the inverse variance method. We presented the results as relative risk (RR) with 95% confidence intervals (CIs) for dichotomous outcomes [21]. We assessed heterogeneity using the I^2^ statistic, the χ^2^ test for homogeneity (p <0.1 for significance of substantial heterogeneity). We considered an I^2^ value greater than 50% indicative of substantial heterogeneity. We investigated further with subgroup analyses to assess clinical and methodological sources of heterogeneity. We assessed for publication bias using Begg’s funnel plots if there are 10 or more studies per outcome [21–23].

Given the heterogeneity, variation and disparate reporting for morbidity, hospitalizations/occupancy, disruptions in standards-of-care, we could not conduct a meta-analysis for these outcomes.

### Subgroup analyses

Potential and expected clinical sources of heterogeneity were explored for selected outcomes (e.g. mortality). When a sufficient number of trials were available (e.g. >10 studies), we conducted the following pre-specified subgroup pooled analyses (hypothesized direction of effect in parentheses):

- High vs. low risk of bias studies (hypothesis: high risk of bias studies would favour pre-pandemic usual care management outcomes).
- High (HIC) vs. low-middle income (LMIC) countries, as defined by World Health Organization [2] (hypothesis: outcomes would favour HIC during both pandemic and pre-pandemic times)
- Acute care hospital vs. jurisdictional/public health/population restrictions/interventions (hypothesis: acute care/public health interventions would be favoured during pandemic times)
- Medical vs. surgical vs. medical/surgical case-mixes (hypothesis: surgical health care interventions would be favoured during pandemic times compared to medical cases)

If subgroups effects were credible, we presented the outcomes separately for each subgroup.

### Dealing with missing data

If we encountered missing data, we attempted to contact the study authors for additional information or clarity. If we could not obtain additional data, we analyzed the available data and reported the potential impact of missing data in the discussion.

## Results

### Study characteristics

**Fig 1:**
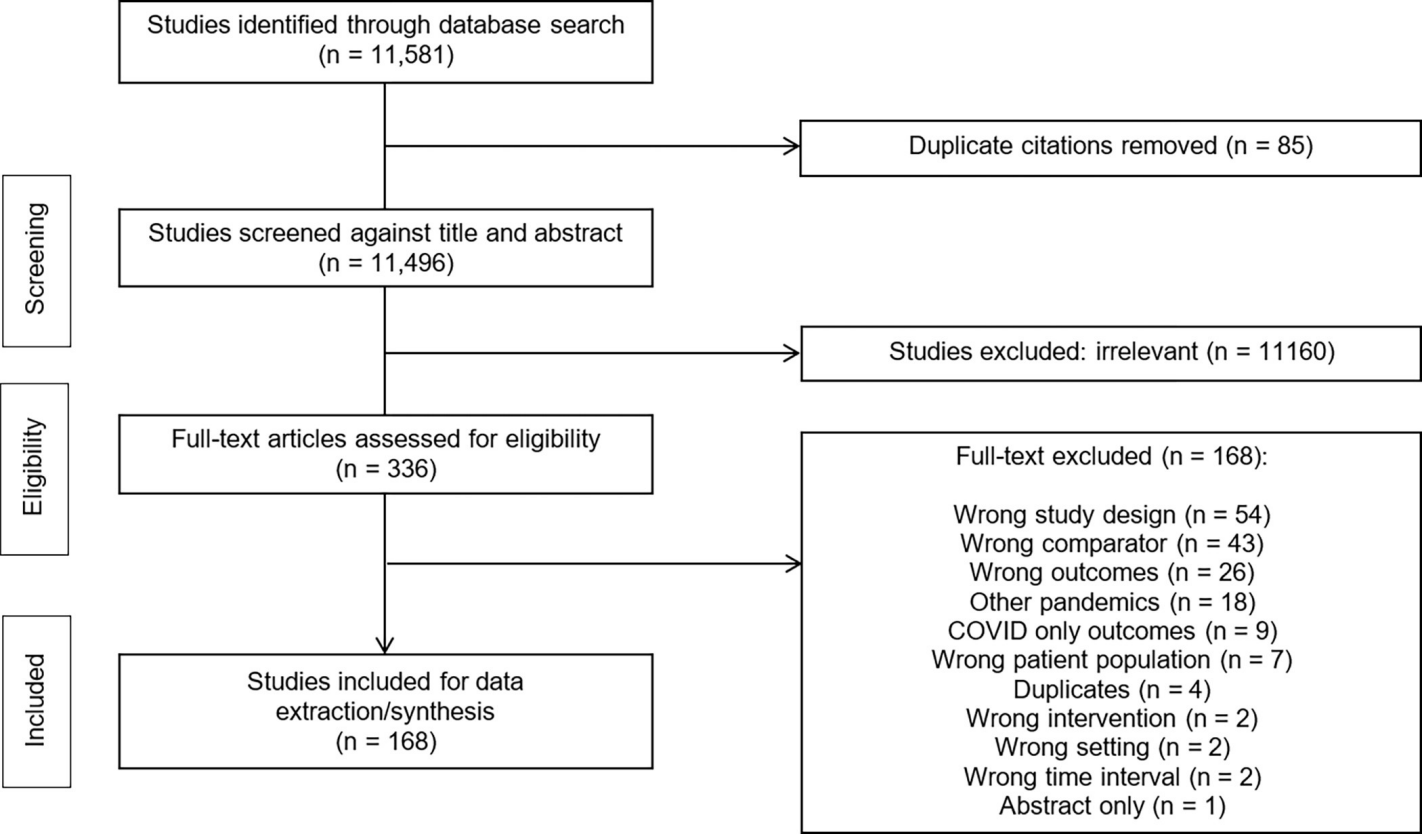
COPES PRISMA flow diagram (non-COVID illness).

Of 11,581 records identified through our search, we reviewed 336 full-texts, and included 167 studies which fulfilled eligibility criteria (Fig 1). Summary of study characteristics are presented in Table 1. A complete list of all collected study data, demographics, baseline characteristics, subgroups and outcomes can be found in S2–S4 Tables [24–188].

**Table 1:** Summary statistics of study design and characteristics for COPES Non-COVID illness during COVID pandemic (n = 168).

**Publication Status**	**n (%)**	**Country**	**n (%)**
Peer-reviewed publication	161 (96%)	Multinational	4 (2%)
Pre-print	6 (4%)	Single	163 (98%)
**Study Design**		**Primary Illness Category**	
Observational (cohort)	164 (98%)	Cardiovascular	51 (30%)
Observational (case-control)	2 (1%)	Mixed multi-illness	45 (27%)
Case-series with control group	1 (1%)	Neurologic	26 (16%)
		Trauma	12 (7%)
**REB approval**		Respiratory	8 (5%)
Yes	91 (54%)	Gastrointestinal	8 (5%)
Waived/not required	46 (27%)	Infectious	5 (3%)
Not reported	25 (16%)	Musculoskeletal/skin and soft tissue	5 (3%)
Not applicable	5 (3.0%)	Urologic	4 (2%)
		Head and neck	3 (2%)
**Consent obtained**		Transplant	2 (1%)
Yes	22 (13%)	Metabolic/toxins	1 (1%)
Waived/not required	76 (45%)	Renal	1 (1%)
Not reported	59 (36%)		
Not applicable	10 (6.0%)	**Subgroups:**	
		**Risk of bias**	
**Funding**		Good (low risk of bias)	25 (15%)
Industry	2 (1%)	Poor (high risk of bias)	142 (85%)
Government	23 (13%)		
Institutional	18 (11%)	**High vs. low/middle income country**	
Non-for-profit	9 (5%)	High	146 (88%)
Other	6 (4%)	Low/middle	21 (12%)
None	75 (45%)		
Not reported	47 (28%)	**Case-Mix**	
		Medical	59 (36%)
**Setting**		Surgical	40 (24%)
Acute care hospital	111 (67%)	Mixed (medical/surgical)	68 (41%)
Emergency department	26 (16%)		
Ward	20 (12%)	**Level of healthcare intervention**	
Intensive care unit	15 (9%)	Acute care hospital level interventions	134 (80%)
Other/Not applicable	22 (13%)	Jurisdiction/public health/population level interventions	33 (20%)

**COPES**: Coronavirus Disease (COVID-19) and Outcomes Associated with Pandemic Effects Study (COPES), **COVID-19**: Coronavirus Disease-2019, **REB**: research ethics board

Of the 167 studies, there were 164 (98%) observational cohort studies and 2 (1%) case-control studies, and 1 (1%) case-series with control groups. The predominant setting for these studies was acute care hospitals (111 studies, 66%). These studies were largely conducted in a single country (163 studies, 97%) with 35 individual countries contributing (highest was the United States with 31 studies) (Table 1).

The top five primary illness categories were as follows: cardiovascular (51 studies, 30%); mixed multi-illness (45 studies, 27%); neurological (26 studies, 16%); trauma (12 studies, 7%); and, respiratory or gastrointestinal (8 studies, 5%), each (Table 1).

### Risk of bias

The risks of bias (RoB) assessments using the Newcastle-Ottawa Scale tools for observational studies are shown in S5A and S5B Table cohort (5A) and case-control (5B), respectively.

For cohort studies (S5A Table), NOS tools revealed full scores for only 14 out of 163 studies (9%). Common deficiencies were found in 150 (92%) studies, with plurality in the following areas: lack of comparability of cohorts (111 studies, 68%), lack of long enough follow-up (113 studies, 69%), and lack of adequate follow-up (111 studies, 68%).

For case-control studies (S5B Table), NOS tools revealed full scores for 1 out of 3 (33%) of studies. Deficiencies were found in comparability of cases and controls, and non-response rate.

## Data synthesis and analysis

### Primary and secondary outcomes and GRADE assessments

Study outcomes are presented in S6 Table, with summary of significant changes in mortality (primary outcome), morbidity, acute care hospitalizations/occupancy and disruptions to care (secondary outcomes) presented in S7 Table. GRADE assessment is shown in Table 2. We found an overall “very low” certainty of evidence for non-COVID illnesses during the COVID-19 pandemic period for all outcomes (mortality, morbidity, acute care hospitalizations/occupancy, disruptions to care).

**Table 2:** Grading of Recommendations Assessment, Development and Evaluation (GRADE) of COPES outcomes: Mortality, morbidity, hospitalizations, disruptions to care.

Certainty assessment							Study Measurements/Results/Impact	Certainty	Importance
№ of studies	Study design (sources)	Risk of bias	In-consistency	Indirect-ness	Im- precision	Other considerations ^a^			
Mortality									
76	Observational studies(74 cohort, 2 case-control) Sample size (76 studies): • 353,539 control patients (pre-pandemic) • 138,323 pandemic period patients	very serious ^b^	serious ^c^	not serious ^d^	not serious ^e^	none ^f^	• ***Study results (meta-analysis*, *76 studies)***: • Absolute effect estimates—mortality events (76 studies): ○ Pandemic: 22,348 deaths/138,323 patients (16%) ○ Pre-pandemic: 40,768 deaths/354,539 patients (11%) ○ Absolute difference: 5% fewer deaths per 100 patients during pre-pandemic period ○ ***Mortality*: *RR 0*.*76 [95% CI*: *0*.*70–0*.*82] favouring pre-pandemic period*, *p <0*.*00001*, *I***^***2***^ ***= 97% (high heterogeneity)*** • *Subgroup analyses*: *persistent statistical significance favouring pre-pandemic period for cardiovascular*, *respiratory*, *trauma/musculoskeletal*, *high & low risk of bias*, *high income countries*, *acute care hospital*, *medical*, *and surgical subgroups* • *The change in mortality outcome was reported in 97 studies*, *of which 50/97 (52%) studies reported a statistically significant change in mortality*. • *RoB was rated as “very serious”–given the high proportion of poor NOS vs*. *good NOS scores* • *There is serious inconsistency in this literature (given the discrepancies (48% of studies did not statistically significant mortality difference)*. *However*, *this means publication bias is unlikely given the extensive and thorough search performed for this SR alongside the balanced findings of both significant and non-significant mortality outcomes* • *Imprecision was rated as “not serious” for imprecision*, *pooled 95% CI does not cross 1*, *and is significantly difference than null (p < 0*.*00001)* • *Given all observational studies start at a “low certainty rating”*, *plus downgrades for RoB*, *inconsistency and imprecision would consider the certainty in the evidence to be “very low” quality for mortality*	⨁◯◯◯ Very Low Quality	CRITICAL
Morbidity									
58	Observational studies (57 cohort, 1 case-control)	very serious ^b^	serious ^c^	not serious ^d^	serious ^e^	none ^f^	• *No meta analyses possible given heterogeneity of morbidity outcomes* • *The change in morbidity outcome was reported in 58 studies*, *of which 28/58 (48%) studies reported a statistically significant change in morbidity*. • *RoB was rated as “very serious”–given the high proportion of poor NOS vs*. *good NOS scores* • *There is serious inconsistency in this literature (given the discrepancies (52% of studies did not statistically significant morbidity difference)*. *However*, *this means publication bias is unlikely given the extensive and thorough search performed for this SR alongside the balanced findings of both significant and non-significant morbidity outcomes* • *Imprecision was rated as serious*, *given as many of the 95% CIs are still wide or cross 1*, *while many p-values or 95% CIs that are reported do not show significance in differences* • *Given all observational studies start at a “low certainty rating”*, *plus downgrades for RoB*, *inconsistency and imprecision would consider the certainty in the evidence to be “very low” quality for morbidity*	⨁◯◯◯ Very Low Quality	CRITICAL
Acute care hospitalizations/capacity/occupancy									
150	Observational studies (147 cohort, 3 case-control)	very serious ^b^	serious ^c^	not serious ^d^	serious ^e^	none ^f^	• *No meta analyses possible given heterogeneity of hospitalization outcomes* • *The change in acute care capacity outcome was reported in 150 studies*, *of which 39/150 (26%) studies reported a statistically significant change in acute care capacity*. • *RoB was rated as “very serious”–given the high proportion of poor NOS vs*. *good NOS scores* • *There is serious inconsistency in this literature (given the discrepancies (74% of studies did not statistically significant acute care capacity difference)*. *However*, *this means publication bias is unlikely given the extensive and thorough search performed for this SR alongside the balanced findings of both significant and non-significant acute care capacity outcomes* • *Imprecision was rated as serious*, *given as many of the 95% CIs are still wide or cross 1*, *while many p-values or 95% CIs that are reported do not show significance in differences* • *Given all observational studies start at a “low certainty rating”*, *plus downgrades for RoB*, *inconsistency and imprecision would consider the certainty in the evidence to be “very low” quality for acute care capacity*	⨁◯◯◯ Very Low	IMPORTANT
Disruptions to care									
124	Observational studies (123 cohort, 1 case-control)	very serious ^b^	serious ^c^	not serious ^d^	serious ^e^	none ^f^	• *No meta analyses possible given heterogeneity of disruptions in care outcomes* • *The change in disruptions to care outcome was reported in 124 studies*, *of which 62/125 (50%) studies reported a statistically significant change in disruptions to care*. • *RoB was rated as “very serious”–given the high proportion of poor NOS vs*. *good NOS scores* • *There is serious inconsistency in this literature (given the discrepancies (50% of studies did not statistically significant disruptions to care)*. *However*, *this means publication bias is unlikely given the extensive and thorough search performed for this SR alongside the balanced findings of both significant and non-significant disruptions to care* • *Imprecision was rated as serious*, *given as many of the 95% CIs are still wide or cross 1*, *while many p-values or 95% CIs that are reported do not show significance in differences* • *Given all observational studies start at a “low certainty rating”*, *plus downgrades for RoB*, *inconsistency and imprecision would consider the certainty in the evidence to be “very low” quality for disruptions to care*	⨁◯◯◯ Very Low Quality	IMPORTANT

**CI:** confidence interval, **GRADE:** Grading of Recommendations Assessment, Development and Evaluation, **NOS:** Newcastle-Ottawa Scale, **RoB:** risk of bias, **SR:** systematic review

a. Other considerations: e.g. publication bias, large magnitude of effect, dose-response gradient, all plausible confounding would reduce the demonstrated effect or increase the effect if no effect was observed

b. “Very serious” rating based on poor RoB in 85.2%, and only good RoB in 14.8% of all studies (n = 169)

c. “Serious” rating based on overall inconsistency (specifically there are large discrepancies for differences in all outcomes: mortality (51.0% statistically significant change vs. 49.0% not), morbidity (64.1% statistically significant change vs. 35.9% not), acute care hospitalizations/capacity/occupancy (25.8% statistically significant change vs. 74.2% not), and disruptions in care (50.0% statistically significant change vs. 50% not)

d. “Not serious” rating for indirectness, given all studies measured directly at the 4 *a priori* outcomes (mortality, morbidity, acute care hospitalizations/capacity/occupancy and disruptions to care)

e. “Not serious” for imprecision, pooled 95% CI does not cross 1, and is significantly difference than null (p < 0.00001)

f. There is unlikely to be any significant other considerations. Publication bias is unlikely to be present, given the extensive search during this SR, alongside finding which demonstrate both increases and decreases in various outcomes (mortality, morbidity, acute care hospitalizations/capacity/occupancy and disruptions to care). Furthermore, there is also no consistent large magnitude of effect, dose-response gradient, and many studies still have residual confounding.

**Fig 2:**
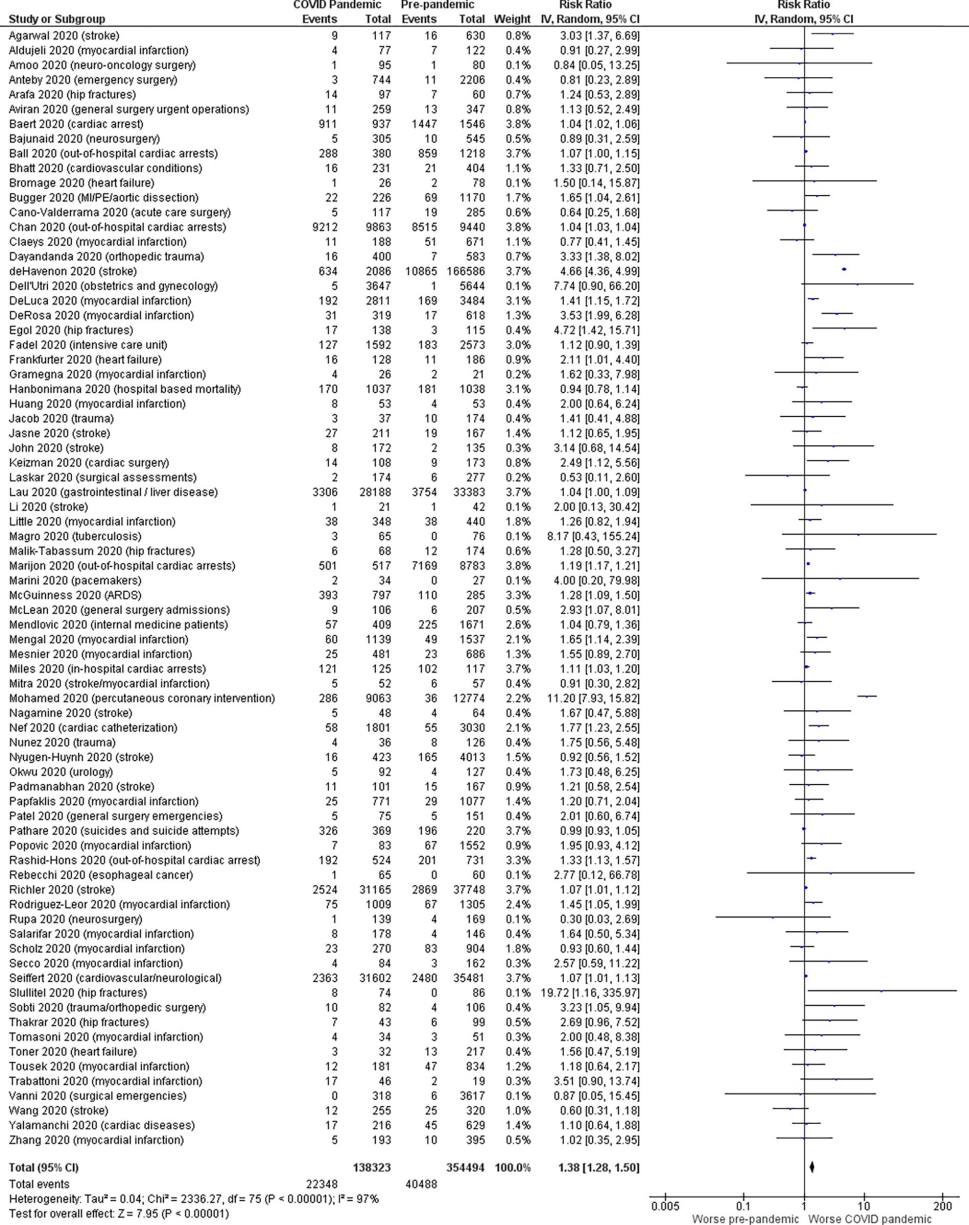
Forest plot for overall mortality (meta-analysis).

For overall mortality (Fig 2), our meta-analysis (74 observational studies reporting mortality counts, 491,862 patients) demonstrated an increase mortality of 16% during the COVID pandemic compared to 11% mortality during the pre-pandemic period for non-COVID illness (RR 1.38, 95% CI: 1.28–1.50; absolute risk difference: 5% [95% CI: 4–6%], p<0.00001, I^2^ = 97%). This observation was consistent for grouped systems including: cardiovascular (RR 1.27, 95% CI: 1.19–1.35; p<0.00001, 34 studies); respiratory (RR 1.28, 95% CI: 1.09–1.50; p = 0.003, 1 study); and trauma/musculoskeletal (RR 2.21, 95% CI: 1.50–3.24; p<0.0001, 9 studies).

Fifty studies (29.8%) reported a statistically significant change in mortality, while 47 studies (28.0%) reported no significant change, and 71 studies (42%) did not report on mortality. Of those 50 studies which reported a significant change in mortality, 49 studies (98.0%) reported an increase in mortality, while one study (2%) reported a decrease in mortality during the COVID-19 pandemic compared with non-COVID-19 pre-pandemic historical controls. Ninety-seven observational studies reporting mortality (starting at “low” quality of evidence) were downgraded for RoB (“very serious” RoB due to high proportion of poor NOS scores), and inconsistency (high heterogeneity). This led to a “very low” level of certainty in the quality of evidence.

Twenty-eight studies (17%) reported a statistically significant change in morbidity, while 30 studies (18%) reported no significant change, and 110 studies (66%) did not report on morbidity. Of those 28 studies which reported significant changes in morbidity, 26 studies (93%) reported an increase in morbidity, while two studies (7%) reported a decrease in morbidity during the COVID-19 pandemic compared with non-COVID-19 pre-pandemic historical controls. Morbidity was reported in 58 observational studies, where we similarly downgraded for RoB (“very serious” RoB due to high proportion of poor NOS scores), inconsistency (high heterogeneity), and imprecision (wide confidence intervals). This led to a “very low” level of certainty in the quality of evidence.

Thirty-nine studies (23%) reported a statistically significant change in acute care hospitalizations/occupancy, while 111 studies (66%) reported no significant change, and 18 studies (10%) did not report on hospitalizations/occupancy. Of those 39 studies which reported statistically significant change in hospitalizations/occupancy, one study (3%) reported an increase in hospitalizations/occupancy, while 38 studies (97%) reported a decrease in hospitalizations/occupancy during the COVID-19 pandemic compared with non-COVID-19 pre-pandemic historical controls. Hospitalizations and occupancy were reported in 150 observational studies, which were downgraded for RoB (“very serious” RoB due to high proportion of poor NOS scores), inconsistency (high heterogeneity), and imprecision (wide confidence intervals). This led to a “very low” level of certainty in the quality of evidence.

Sixty-two studies (37%) reported a statistically significant change in disruptions to care, while 62 studies (37%) reported no significant change, and 43 studies (26%) did not report on disruptions to care. Of those 62 studies which reported on disruptions to care, 47 studies (76%) reported an increase in disruptions to care, while 15 studies (24%) reported a decrease in disruptions to care (with surgical and elective procedure delays/cancellations and delays to presentation/treatment being the most common reasons) during the COVID-19 pandemic compared with non-COVID-19 pre-pandemic historical controls. Disruptions in standards-of-care were reported in 124 observational studies, where we downgraded for RoB (“very serious” RoB due to high proportion of poor NOS scores), inconsistency (high heterogeneity), and imprecision (wide confidence intervals). This led to a “very low” level of certainty in the quality of evidence.

### Subgroups

Pre-specified subgroup analyses for mortality are shown S4 Table with subgroup Forest plots shown in S1–S5 Figs.

For RoB (S2 Fig), there was a similar increase in mortality for both studies with a high (RR 1.37, 95% CI: 1.19–1.54; p<0.00001, 62 studies) and low RoB (RR 1.46, 95% CI: 1.30–1.63; p<0.00001, 14 studies).

For HIC vs. LMIC countries (S3 Fig), there was a similar increase in mortality for HIC during the COVID-19 pandemic compared with non-COVID-19 pandemic historical controls (RR 1.42, 95% CI: 1.30–1.54; p<0.00001, 71 studies). However, LMIC showed no difference in mortality (RR 1.10, 95% CI: 0.87–1.38; p = 0.42, 5 studies).

For level of healthcare intervention (S4 Fig), there was a similar increased mortality for acute care hospital settings (RR 1.40, 95% CI: 1.29–1.52; p<0.00001, 75 studies) compared with jurisdictional/public health/population restrictions/interventions during the COVID-19 pandemic compared with non-COVID-19 pandemic historical controls. However jurisdictional settings showed no difference in mortality (RR 0.99, 95% CI: 0.93–1.05; p = 0.78, 1 study).

For case-mix (S5 Fig), there was a increase in mortality for both medical (RR 1.38, 95% CI: 1.26–1.51; p<0.00001, 50 studies) and surgical case-mix (RR 1.69, 95% CI: 1.27–2.24; p = 0.0003, 23 studies) during the COVID-19 pandemic compared with non-COVID-19 pandemic historical controls. However, mixed cases showed no difference in mortality (RR 1.12, 95% CI: 0.88–1.44; p = 0.36, 3 studies).

There was no significant change between inverse variance pooling and Mantel-Haenszel Random-Effects Forest Plot (S6 Fig). Mantel-Haenszel fixed-effects model are also shown (S7 Fig), although it is implausible that assumption that true effect was the same across all studies.

### Publication bias

Visual inspection of Begg’s funnel plots did not reveal publication bias for the outcome of mortality (S8 Fig).

## Discussion

In this systematic review of non-COVID illness occurring during the COVID-19 pandemic, patient outcomes were variably affected by the pandemic compared to historical non-pandemic epochs. However, our meta-analysis revealed a significant increase in mortality during the COVID pandemic for non-COVID illness as compared to pre-pandemic time periods (very low certainty evidence), which was consistent across most subgroups evaluated. A substantial proportion of studies reported changes in morbidity; health services and disruptions associated with the pandemic, although this was not universal. The following directional trends were observed: increased morbidity; decreased hospitalizations and lower occupancy; and increased disruptions in care in multiple jurisdictions from low certainty evidence (mainly due to the majority being observational studies with high risk of bias).

While this would preclude strong inferences or definitive recommendations on the nature of the public health interventions and health systems responses to the COVID-19 pandemic crisis, this analysis provides insight into the potential substantial trade-offs that have occurred for both patients with non-COVID illness and health systems capacity to meet standards-of-care. In multiple jurisdictions, excess all-cause mortality (USA: 72 deaths per 100,000, UK: 95 deaths/100,000, Spain: 102 deaths/100,000) has been reported over and above recorded COVID-19 deaths alone [44]. Therein lies the controversy of how the pandemic itself and public health policies around prioritization have had unintended damage to the normal functioning of our health systems and negatively impacted outcomes for non-COVID patients. This is further reinforced by the ethical dilemma of choosing between COVID versus non-COVID patients with scarce healthcare resources [188], especially if triage protocols are enacted [188–192].

Our systematic review adds new knowledge on the potential scope and magnitude of the effects of the COVID-19 pandemic on all non-COVID illness. There is emerging literature that excess mortality is not only driven by COVID-19 deaths [165], but there is also evidence of non-COVID excess mortality and morbidity [193], including in ICU settings [194], secondary to disruptions of global healthcare services by the COVID-19 pandemic [3]. The intensity of disruption (severity multiplied by duration) may have altered the apparent effects among non-COVID illness, leading to the variability observed for different jurisdictions and illnesses. For example, overwhelmed medical systems (e.g., Italy, United States, Brazil, India) may have had higher attributable excess mortality [44, 155], relative to initially less strained jurisdictions (e.g., Australia, New Zealand, Taiwan) by preserving existing healthcare capacity. Jurisdictions experiencing substantially strained healthcare capacity largely prioritized acute care hospitals and intensive care services for surges in COVID pandemic cases [4]. As such, to preserve and generate added capacity (e.g., redeployment of resources), healthcare policy was directed to postpone, delay or cancel elective and non-urgent procedures and scheduled surgeries [195], forced outpatient services to switch to virtual platforms [196], and required unprecedented compromise of entire healthcare systems to meet these challenges. Furthermore, there may be added unmeasured effects of the COVID-19 pandemic that we have not captured or may not be proximally seen (e.g., routine childhood immunization; cancer screening; intimate partner violence; mental health treatments; ethanol and substance abuse), with downstream effects not realized for years to come. Alternatively, it is also plausible that the disruptions caused by the COVID-19 pandemic to the health system have realized new efficiencies and reduced utilization of low-value care (e.g., discretionary diagnostics, imaging and procedures) [197], which may have led to risk of iatrogenic harm by the health care system.

There are fundamental trade-offs that occur when employing public health measures and policies during pandemics. Potential negative effects of the pandemic include affecting social determinants of health (e.g., social isolation, increases in domestic violence, unemployment rates, proportion of populations living in poverty, social security, etc.) alongside healthcare disruption that may have contributed to the overall excess mortality, morbidity, and disruptions in standards-of-care in the non-COVID population. As a society, do we continually tradeoff and prioritize COVID patients at the expense of non-COVID patients, especially those who continue to flaunt public health measures, refuse vaccines and spread misinformation? Are we willing to accept prolonged, sustained disruptions to healthcare systems and society, while continually delay care of non-COVID patients? This is all interwoven and extremely complex pieces of the puzzle within public health policy all need to be weighed such that both COVID and non-COVID patients are not harmed.

Anticipating ongoing global disruptions to healthcare is a key to weathering unanticipated short and long-term COVID-19 pandemic effects to non-COVID patients, which includes: (1) evidence-based, expedited vaccination where available, with mandates quickly implemented; (2) surge capacity planning aimed at: i) creating capacity as needed; ii) preserving acute health system capacity for non-pandemic illnesses; iii) attending to non-acute healthcare systems needs that were lower priority (e.g. social determinants, etc.). This systematic review highlights the potential unintended and collateral effects on health services access, care quality and outcomes for patients with non-COVID-related illness [10], and should spark further research and debate on how to achieve balance alongside determining healthcare policy between pandemic response and non-pandemic population health, particularly given the continued spread of emerging variants of concern contributing to prolongation of the pandemic [198].

The strengths of our SR include a comprehensive search strategy and a rigorous process for study selection and data abstraction based on an *a priori* protocol, with due consideration to study quality, risk of bias and overall certainty of the evidence using GRADE alongside our meta-analysis methodology.

This SR also has several limitations, most of which relate to limitations of the primary studies analyzed. As mentioned, given the heterogeneity and variable reporting, we could not conduct a meta-analysis for all outcomes. GRADE certainty of evidence was very low for all outcomes, driven primarily by many studies with high risk of bias (with the majority of included studies being observational in nature, without adjustment for baseline characteristics and illness severity) and inconsistency (high heterogeneity in jurisdictional responses to COVID). Delayed or lack of presentation to acute care hospitals may have resulted in increased death out of hospital with death upon arrival or no transfer to acute care facility, which may have biased findings due to under-reporting. Moreover, there is both likelihood of underreporting in the literature and temporal delays in further publications describing health systems effects of the COVID pandemic on non-COVID illnesses [199]. Furthermore, the time-horizon for mortality, morbidity and disruption will likely be far longer than has been captured in the studies to date, with the full scope of effects requiring longer periods for observation. Accordingly, these results must be interpreted carefully and within context.

## Conclusion

The COVID-19 pandemic had variable associations with non-COVID illness patient outcomes (e.g., mortality, morbidity, acute care hospitalizations/occupancy and disruptions in standards-of-care) in multiple jurisdictions (very low certainty). Where significant changes were described, there was evidence of increased mortality, increased morbidity, decreased acute care hospitalizations/occupancy and increased disruptions in care across variations in case-mix and multiple jurisdictions (very low certainty). Informing healthcare policy and decision-makers of the potential pandemic effects is crucial to mitigate the impact of the COVID-19 pandemic on both COVID and non-COVID patients.

## Supporting information

S1 Appendix COPES systematic review search strategy. (DOCX)

S1 Table COPES PRISMA checklist. (DOC)

S2 Table Characteristics of non-COVID papers (pre-pandemic vs. pandemic periods). (DOCX)

S3 Table REB, consent, funding for included studies. (DOCX)

S4 Table Subgroups. (DOCX)

S5 Table Section A—Risk of Bias Assessment for Observational Cohort Studies–Newcastle-Ottawa Score, Section B: Risk of Bias Assessment for Observational Case-Control Studies–Newcastle-Ottawa Score. (DOCX)

S6 Table Mortality, morbidity, hospitalizations/occupancy, disruption in care outcomes (with statistical significance). (DOCX)

S7 Table Summary statistics of statistically significant outcomes for COPES Non-COVID Illness during COVID pandemic. (DOCX)

S1 Fig Forest plot for subgroup analysis by admission type (mortality). (TIF)

S2 Fig Forest plot for subgroup analysis by risk of bias (mortality). (TIF)

S3 Fig Forest plot for subgroup analysis by high vs. low/middle income countries (mortality). (TIF)

S4 Fig Forest plot for subgroup analysis by hospital vs. jurisdictional interventions (mortality). (TIF)

S5 Fig Forest plot for subgroup analysis by case mix (mortality). (TIF)

S6 Fig Mantel-Haenszel random-effects forest plot. (TIF)

S7 Fig Mantel-Haenszel fixed-effects forest plot. (TIF)

S8 Fig Assessment of publication bias (Begg’s funnel plot). (TIF)
