# Supplementary material for: Non-COVID outcomes associated with the coronavirus disease-2019 (COVID-19) pandemic effects study (COPES): A systematic review and meta-analysis
Source: PLoS One. 2022 Jun 24;17(6):e0269871. doi: 10.1371/journal.pone.0269871 (PMC9231780; doi:10.1371/journal.pone.0269871)
Supplement: S1 Appendix — (DOCX) [file pone.0269871.s001.docx]

**Supplemental Appendix 1**: COPES Systematic Review Search Strategy

Database(s): **Ovid MEDLINE(R) ALL** 1946 to Dec 31, 2020

Search Strategy:

**# Searches Results**

1 "10.1093/aje/kwx203 " [DOI] 1

2 "10.1136/heartjnl-2020-317062" [DOI] 1

3 "10.1097/BOT.0000000000001845 " [DOI] 1

4 "10.1016/j.cjca.2020.05.023" [DOI] 1

5 "10.1016/j.cjca.2020.05.003 " [DOI] 1

6 "10.4414/smw.2020.20314 " [DOI] 1

7 "10.1016/j.jamda.2020.05.045" [DOI] 1

8 "Minimal impact of COVID-19 outbreak on the postoperative morbidity and mortality" [Article Title] 1

9 "10.2807/1560-7917.ES.2020.25.28.2001239" [DOI] 1

10 "10.1016/j.jacc.2020.05.038" [DOI] 1

11 "10.3390/ijerph17124392" [DOI] 1

12 "10.1002/uog.22088 " [DOI] 1

13 "Excess all-cause mortality during the COVID-19 pandemic in Europe" [Article Title] 1

14 "10.3390/ijerph17124233" [DOI] 1

15 "10.1016/S0140-6736(20)31234-4 " [DOI] 1

16

"Excess Mortality Estimation During the COVID-19 Pandemic: Preliminary Data from Portugal" [Article

Title]

4

17 "10.1136/bmj.m1835" [DOI] 1

18

"COVID-19 in Italy: impact of containment measures and prevalence estimates of infection" [Article

Title]

1

19 "10.3390/ijerph17103452 " [DOI] 1

20 "10.1002/14651858.CD013574" [DOI] 1

21 "10.2807/1560-7917.ES.2020.25.19.2000620 " [DOI] 1

22 "10.1186/1471-2458-13-211 " [DOI] 1

23 "10.1016/j.annepidem.2017.12.005 " [DOI] 1

24 "10.1016/j.annepidem.2017.12.009 " [DOI] 1

25 "10.1007/s10654-012-9701-y" [DOI] 1

26 "10.1017/S0950268811002238 " [DOI] 1

27 "10.1017/S0950268811001968 " [DOI] 1

28 "10.1186/1471-2458-11-41 " [DOI] 1

29 "10.3201/eid1609.091723 " [DOI] 1

30 or/1-29 [TEST ARTICLES] 32

31 Coronavirus Infections/ 22661

32 Coronavirus/ 3149

33 Betacoronavirus/ 15320

34 (coronavir* or corona-virus*).tw,kf. 29941

35 betacoronavir*.tw,kf. 403

36 (covid19 or covid-19).tw,kf. 39265

37 (nCov or novel-CoV or 2019nCoV).tw,kf. 1162

38 (CoV-2 or CoV2 or sarscov2).tw,kf. 13790

39 Wuhan-virus*.tw,kf. 10

40 ((wuhan or hubei or huanan) and (severe-acute-respiratory or pneumonia*) and outbreak*).tw,kf. 619

41 or/31-40 [COVID-19 FILTER ADAPTED FROM THE CDC] 57818

42 Influenza Pandemic, 1918-1919/ 132

43 pandemic*.tw,kf. 42672

44 non-pandemic*.tw,kf. 106

45 or/42-44 [GENERAL PANDEMICS] 42706

46 41 or 45 [COVID AND OTHER PANDEMICS] 82997

47 exp Mortality/ 383206

48 Hospital Mortality/ 39870

49 Maternal mortality/ 10329

50 Hospitalization/sn 31896

51 Cause of Death/ 49016

52 Survival Analysis/ 135796

53 Time-to-Treatment/ 6832

54 Survival Rate/ 173372

55 fatal outcome*.tw,kf. 9306

56 (mortalit* adj2 (attribut* or excess* or in-hospital or rate*)).tw,kf. 170838

57 (morbidit* adj2 (attribut* or excess* or in-hospital or rate*)).tw,kf. 18703

58 premature death*.tw,kf. 7072

59 ((health* or care*) adj2 disrupt*).tw,kf. 982

60 (health* adj3 deliver*).tw,kf. 45710

61 postpon*.tw,kf. 10826

62 or/47-61 [Outcomes] 730183

63 46 and 62 4571

64 30 and 63 32

65 30 not 64 0
