# Supplementary material for: Non-COVID outcomes associated with the coronavirus disease-2019 (COVID-19) pandemic effects study (COPES): A systematic review and meta-analysis
Source: PLoS One. 2022 Jun 24;17(6):e0269871. doi: 10.1371/journal.pone.0269871 (PMC9231780; doi:10.1371/journal.pone.0269871)
Supplement: S2 Table — (DOCX) [file pone.0269871.s003.docx]

**S2 Table: Characteristics of non-COVID papers (pre-pandemic vs. pandemic periods)**

| **Study Author (year)** | **Study Design** | **Country** | **Sample size (pandemic)** | **Sample size (pre-pandemic)** | **Age [mean (SD) or median (IQR)]: pandemic vs. pre-pandemic** | **Female (%)** | **Primary illness category** | **Patient population (primary illness)** | **Pandemic time period** | **Non-pandemic time period** |
| --- | --- | --- | --- | --- | --- | --- | --- | --- | --- | --- |
| Abdelaziz 2020 | Observational (cohort) | UK | 46 | 69 | 63 years (11) vs. 67 years (12) | 30% vs. 24% | Cardiovascular | Myocardial infarction | Mar 1-31, 2020 | Mar 1, 2019 - Mar 31, 2019 |
| Agarwal 2020 | Observational (cohort) | India | 2,200 | 3,398 | 29 years (IQR: 0-78) vs. 55 years (IQR: 0-92) | 39% vs. 34% | Multiple illnesses (other) | Ophthalmologic pathology | Mar 25-Jul 15, 2020 | Mar 25, 2019 -Jul 15, 2019 |
| Agarwal 2020 | Observational (cohort) | US | 120 | 634 | 68 years (IQR: 58-79), 72 years (IQR: 60-81) | 51% vs. 45% | Neurological | Stroke | Mar 1 - May 15, 2020 | Jun 2019 - Feb 2020 |
| Aldujeli 2020 | Observational (cohort) | Lithuania | 77 | 122 | STEMI: 67 years (59-76) vs. 69 (59-83), NSTEMI: 70 years (64-80) vs. 70 (60-79) | STEMI: 28% vs. 35%, NSTEMI: 27% vs. 40% | Cardiovascular | Myocardial infarction | Mar 11 - Apr 20, 2020 | Mar 11, 2019 - Apr 20, 2019 |
| Amaddeo 2020 | Observational (cohort) | France | 293 | 377 | 67 years (IQR: 60-73) vs. 67 years (IQR: 60-74) | 16% vs. 18% | Gastrointestinal | Hepatocellular carcinoma | Mar 6 - Apr 17, 2020 | Mar 6, 2019 - Apr 17, 2019 |
| Amoo 2020 | Observational (cohort) | Ireland | 95 | 80 | 55 years vs. 58 years | 46% vs. 55% | Neurological | Neuro-oncology/glioma surgery | Mar 1 - May 31, 2020 | Mar 1, 2019 - May 31, 2019 |
| Amukotuwa 2020 | Observational (cohort) | Australia | 277 | 2,125 | 66 years (17) vs. 68 (17) | 43% vs. 48% | Neurological | Stroke | Mar 1 - May 10, 2020 | Mar 1, 2019 - Jan 31, 2020 |
| Andersson 2020 | Observational (cohort) | Denmark | 1,118 | 4,296 | Early period: 75 (13) vs. 74 (13), Late period: 73 (13) vs. 73 (13) | 39% vs. 38% | Cardiovascular | Heart failure | Mar 12 - 31, 2020 | Jan 1, 2019 - Mar 11, 2020 |
| Anteby 2020 | Observational (cohort) | Israel | 774 | 1,142 | NR | 48% vs. 43% | Multiple illnesses (other) | Surgical complaints | Feb 1 - Mar 31, 2020 | Feb 1, 2019 - Mar 31, 2019 |
| Arafa 2020 | Observational (cohort) | UK | 97 | 60 | COVID+ 2020: 86 (8) vs. COVID- 2020: 83 (8) vs. 2019: 83 (8) | COVID+ 2020: 52% vs. COVID- 2020: 73% vs. 2019: 68% | Musculoskelal/soft tissue | Hip fractures | Mar 1 - May 31, 2020 | Mar 1, 2019 - May 31, 2019 |
| Athiel 2020 | Observational (cohort) | France | 38,925 | 49,077 | NR | 100% vs. 100% | Multiple illnesses (other) | Gynecological emergencies | Jan 1 - May 31, 2020 | Jan 1, 2019 - May 31, 2019 |
| Aviran 2020 | Observational (cohort) | Israel | 259 | 347 | 60 years (21) vs. 57 (20) | 55% vs. 49% | Multiple illnesses (other) | General surgical operations | Mar 15 - Apr 14, 2020 | Mar 15, 2019 - Apr 14, 2019 |
| Baert 2020 | Observational (cohort) | France | 1,005 | 1,620 | 69 years (17) vs. 69 years (17) | 33% vs. 34% | Cardiovascular | Out-of-hospital cardiac arrest | Mar 1 - Apr 31, 2020 | Mar 1, 2019 - Apr 31, 2019 |
| Bajunaid 2020 | Observational (case-control) | Saudi Arabia | 305 | 545 | 34.9 years (23) vs. 37.0 (22.5) | 38% vs. 45% | Neurological | Neurosurgical procedures | Mar 11 - Apr 30, 2020 | Mar 11, 2019 - Apr 30, 2019 |
| Ball 2020 | Observational (cohort) | Australia | 380 | 1,218 | 69 years (54-80) vs. 67 years (52-78) | 34% vs. 31% | Cardiovascular | Out-of-hospital cardiac arrest | Mar 16 - May 12, 2020 | Mar 16, 2017 - May 12, 2019 |
| Barten 2020 | Observational (cohort) | Netherlands | 10,347 | 12,626 | NR | NR | Multiple illnesses (other) | Emergency department utilization | Feb 15 - Apr 15, 2020 | Feb 15, 2019 - Apr 15, 2019 |
| Batra 2020 | Observational (cohort) | India | 21 | 193 | 49 years (IQR: 35-82) vs. 47 years (IQR: 17-80) | 33% COVID vs 15% Non Covid | Head and neck | Tracheostomy from head and neck cancer surgery | May 19, 2020 - Jun 30, 2020 | Mar 2019 - Feb 2020 |
| Becq 2020 | Observational (cohort) | France | 331 | 402 | 62.3 years (17.9) vs. 62.1 (17.9) | 32% vs. 33% | Gastrointestinal | Upper Gastrointestinal Bleed | Jan 17, 2020 - Apr 17, 2020 | Jan 17, 2019 - Apr 17, 2019 |
| Benites-Goni 2020 | Observational (cohort) | Peru | 65 | 154 | 66 years (median) vs. 70 years | 26% vs. 53% | Gastrointestinal | Upper Gastrointestinal Bleed | Jan 3, 2020 - June 6, 2020 | Oct 19, 2019 – Feb 29, 2020 |
| Bhatt 2020 | Observational (cohort) | USA | 231 | 6,256 | 70 years (15) | NR | Cardiovascular | Acute Cardiovascular Hospitalizations | Mar 1, 2020 - Mar 31, 2020 | Jan 2019 - Mar 2020 |
| Bilinski 2020 | Observational (cohort) | Multinational | NA | NA | NR | NR | Multiple illnesses (other) | COVID-19 and all-cause mortality | Feb 13, 2020 - Sep 19, 2020 | Feb 13, 2015 - Sep 19, 2019 |
| Birkmeyer 2020 | Observational (cohort) | USA | 505,060 | 505,060 | NR | NR | Multiple illnesses (other) | Total medical admissions | Jan 1, 2020 - Jul 11, 2020 | Jan 1, 2019 - Jul 11, 2019 |
| Blangiardo 2020 | Observational (cohort) | Italy | 136,420 | NA | NR | NR | Multiple illnesses (other) | All-cause mortality | Jan 1, 2020 - Apr 28, 2020 | Jan 1, 2016 - Apr 28, 2019 |
| Boyarsky 2020 | Observational (cohort) | USA | 1,673 | NA | NR | 30% | Renal/urological | Living and deceased donor kidney transplants | Mar 1, 2020 - Apr 30, 2020 | Mar 1, 2016 - Apr 30, 2019 |
| Bromage 2020 | Observational (cohort) | UK | 26 | 78 | 74 years (14) vs. 71 (15) | 46% vs. 42% | Cardiovascular | Acute heart failure | Mar 2, 2020 - Apr 19, 2020 | Mar 2, 2019 - Apr 19, 2019 |
| Bugger 2020 | Observational (cohort) | Austria | 226 | 1,170 | 68.6 (13.4) vs. 68.7 (14.6) | 37% vs. 35% | Cardiovascular | Hospital admission for myocardial infarction, pulmonary embolism, abdominal aortic dissection | Mar 16, 2020 - Apr 26, 2020 | Mar 16, 2016 - Apr 26, 2019 |
| Bustos Sierra 2020 | Observational (cohort) | Belgium | 11,492,641 | NA | NR | NR | Multiple illnesses (other) | All-cause mortality | Mar 2, 2020 - Jun 21, 2020 | Jan 1900 - Jun 2020 |
| Butt 2020 | Observational (cohort) | Qatar | 43,175 | 58,858 | NR | NR | Cardiovascular | Patients presenting to the ED with "acute chest pain" suggestive of cardiac origin | Mar 1, 2020 - Apr 30, 2020 | Mar 1, 2019 - Apr 30, 2019 |
| Butt 2020 | Observational (cohort) | Qatar | 5,549 | 13,340 | NR | NR | Multiple illnesses (other) | Acute appendicitis, ACS, other cardiovascular disease diagnoses (including cardiac arrhythmias, congestive heart failure, and angina pectoris without acute myocardial infarction), stroke, acute bone fractures, cancers, live births, and respiratory tract infections (excluding tuberculosis) | Mar 1, 2020 - Mar 30, 2020 | Mar 1, 2019 - Jan 30, 2020 |
| Calderon-Larranaga 2020 | Observational (cohort) | Sweden | NR | 2,379,792 | NR | NR | Multiple illnesses (other) | All-cause mortality | Mar 1, 2020 - May 18, 2020 | Mar 1, 2015 - May 18, 2019 |
| Cannata 2020 | Observational (cohort) | UK | 578 | 1,372 | 78 years (12) vs. 77 years (13) | 46% vs 55% | Cardiovascular | Heart Failure hospitalizations | Jan 7, 2020 - Jun 14, 2020 | Jan 7, 2019 - Jun 14, 2019 |
| Cannavo 2020 | Observational (cohort) | Italy | 32 | 139 | 60 years (14) vs. 58 (10) | 59% vs. 40% | Transplant | All solid organ donation | Feb 21, 2020 - Apr 3, 2020 | Feb 21, 2019 - Feb 20, 2020 |
| Cano-Valderrama 2020 | Observational (cohort) | Spain | 117 | 285 | 54 years | 46% | Multiple illnesses (other) | Acute care surgery in the department of surgery | Mar 16, 2020 - Apr 26, 2020 | Mar 16, 2019 - Apr 26, 2019 |
| Casalino 2020 | Observational (cohort) | France | 101,920 | 266,342 | NR | NR | Multiple illnesses (other) | Emergency department presentations | Nov 26, 2019 - Mar 26, 2020 | Nov 5, 2018 - Nov 25, 2019 |
| Cates 2020 | Observational (cohort) | USA | 3,948 | 5,453 | 70 years (IQR: 61-77) vs. 69 years (IQR: 61-75) | 6% vs 6% | Respiratory | COVID-19 Admissions vs Influenza Admissions | Jan 3, 2020 - Jul 31, 2020 | Oct 1, 2018 - Feb 1, 2020 |
| Cevallos-Valdiviezo 2020 | Observational (cohort) | Ecuador | 36,922 | NA | NR | NR | Multiple illnesses (other) | All-cause mortality | Jan 1, 2020 - Oct 22, 2020 | Jan 1, 2014 - Mar 16, 2020 |
| Chan 2020 | Observational (cohort) | Hong Kong/China | 2,051 | 5,844 | 45 years | NR | Respiratory | Chronic Obstructive Pulmonary Disease (COPD) | Jan - Mar, 2020 | Jan - Mar, 2015-2019 |
| Chan 2020 | Observational (cohort) | USA | 9,863 | 9,440 | 63 years (19) vs. 62 years (19) | 39 vs. 37% | Cardiovascular | Out of hospital cardiac arrest (OHCA) | Mar 16, 2020 - Apr 30, 2020 | Mar 16, 2019 - Apr 30, 2019 |
| Claeys 2020 | Observational (cohort) | Belgim | 116 | 479 | 63 (12) years vs. 63 years (15) | 20% vs 26% | Cardiovascular | ST-elevated Myocardial Infarction (STEMI) | Mar 13, 2020 - Apr 3, 2020 | Mar 13, 2017 - Apr 3, 2019 |
| D'Apolito 2020 | Observational (cohort) | Italy | 166 | 706 | NR | NR | Musculoskelal/soft tissue | Hip and knee arthroplasty [joint dysfunction] - Total joint arthroplasty (TJA)  Allowed operations: septic arthritis, progressive arthritis, TJA dislocations, severe loosening | Feb 24, 2020 - Apr 10, 2020 | Feb 24, 2019 - Apr 10, 2019 |
| Davies 2020 | Observational (cohort) | Scotland | 258 | 2,457 | 59 years vs. 56 years | 62% vs. 55% | Musculoskelal/soft tissue | Elective orthopedic surgeries: total hip replacement, total knee replacement, carpal tunnel decompression | Mar 27, 2020 - Aug 10, 2020 | Mar 27, 2018 - Aug 10, 2019 |
| Dawoud 2020 | Observational (cohort) | England | 125 | 125 | 29 years (18) vs. 34 years (20) | 54% vs. 50% | Head and neck | Cervicofacial infection of odontogenic origin | Mar 16, 2020 - Jun 15, 2020 | Mar 16, 2019 - Jun 15, 2019 |
| Dayananda 2020 | Observational (cohort) | UK | 499 | 604 | 55 years (IQR: 29-75) vs. 44 (IQR: 16-71) | 53% vs. 44% | Trauma/orthopedic | Trauma/orthopedic injuries (by anatomical region): foot, ankle, leg, knee, hip, spine, shoulder, elbow, forearm, hand/wrist, polytrauma, femoral fractures | Mar 16, 2020 - May 31, 2020 | Mar 16, 2019 - May 31, 2019 |
| deHavenon 2020 | Observational (cohort) | USA | 2,612 | 166,586 | NA | 42% vs. 49% | Neurological | Ischemic stroke and comorbid COVID-19 | Apr 1, 2020 - Jul 31, 2020 | Jan 1, 2019 - Dec 31, 2019 |
| Dell'Utri 2020 | Observational (cohort) | Italy | 3,647 | 5,644 | NA | 100% | Renal/urological | Obstetric or gynecologic complaints in emergency services setting | Feb 24, 2020 - May 31, 2020 | Feb 24, 2019 - May 31, 2019 |
| DeLuca 2020 | Observational (cohort) | Multinational (18 European) | 2,956 | 3,653 | 64 years (IQR: 55-73) vs. 64 (IQR 55-73) | 26% vs. 26% | Cardiovascular | ST -segment elevation myocardial infarction treated by primary angioplasty (including mechanical reperfusion for failed thrombolysis) & subpopulation of STEMI patients with arterial hypertension | Mar 1, 2002 - Apr 30, 2020 | Mar 1, 2019 - Apr 30, 2019 |
| DeRosa 2020 | Observational (cohort) | Italy | 319 | 618 | 67 years vs. 65 years | 24% vs. 28% | Cardiovascular | Acute myocardial infarction (AMI): measured ST segment elevation myocardial infarction (STEMI); and non-ST segment elevation MI (NSTEMI), heart failure, heart failure (HF), atrial fibrillation (AF), failure of the implantable device (DF), and pulmonary embolism (PE) | Mar 12-19, 2020 | Mar 12-19, 2019 |
| D'Urbano 2020 | Observational (cohort) | Italy | 27 | 27 | 64 years (IQR: 30-87) vs. 65 (IQR: 20-92) | 45% vs. 46% | Multiple illnesses (other) | Laparoscopic or laparotomy cholecystectomy, appendectomy, hemothorax/pneumothorax/pleural effusion, GI perforation, bowel obstruction, bowel infarction, GI bleeding, Fasciotmy/abscess drainage/hematoma drainage, other surgery | Mar 9, 2020 - Apr 9, 2020 | Mar 9, 2019 - Apr 9, 2019 |
| Egol 2020 | Observational (cohort) | USA | 138 | 115 | 83 years (10) | 64% vs. 68% | Musculoskelal/soft tissue | Hip fractures | Feb 1, 2020 - Apr 15, 2020 | Feb 1, 2019 - Apr 15, 2019 |
| Eshraghian 2020 | Observational (cohort) | Iran | 124 | 230 | 51 years (15) vs. 51 (14) | 18% vs. 3% | Gastrointestinal | Liver-related conditions defined as: acute hepatitis requiring Hospital admission; complications in liver transplant recipients; and complications of liver cirrhosis including gastrointestinal bleeding, spontaneous bacterial peritonitis, hepatorenal syndrome, hepatic encephalopathy, and diuretic resistant ascites | Feb 19, 2020 - Apr 30, 2020 | Feb 19, 2019 - Apr 30, 2019 |
| Fadel 2020 | Observational (cohort) | USA | 1,592 | 2,573 | 64 years (17) vs. 64 years (17) | 50% vs. 50% | Multiple illnesses (other) | Acute respiratory distress syndrome (ADRS), chronic obstructive pulmonary disease (COPD)/Acute exacerbation of COPD, Pulmonary embolism, Sepsis all sources, Pneumonia all causes, hypertensive emergency, cardiac arrest, arrhythmias, CHF exacerbation, Alcohol and/or drug overdose/abuse, diabetic ketoacidosis, Cerebrovascular accident, seizures, upper G.I. bleeding, acute kidney injury, Trauma all causes, cardiogenic shock, asthma exacerbation, chest pain, acute pancreatitis, Lower G.I. bleeding | Mar 15, 2020 - Apr 30, 2020 | Mar 15, 2019 - Apr 30, 2019 |
| Frankfurter 2020 | Observational (cohort) | Canada | 1,106 | 800 | 75 years (median) | 51% | Cardiovascular | Heart failure | Mar 1 - April 19, 2020 | Mar 1, 2019 - Apr 19, 2019 |
| Friedman 2020 | Observational (cohort) | Mexico | 329 | 1,569 | 58 years (17.4) vs. 59.2 (20.1) | 32% vs 32% | Respiratory | Excess Out-of-Hospital Mortality | Apr 14, 2020 - May 11, 2020 | Jan 1, 2019 - Dec 31, 2019 |
| Giannouchos 2020 | Observational (cohort) | USA | 30,276 | 32,937 | 43 years (18) | 54.0% | Multiple illnesses (other) | Emergency department utilization | Jan 1, 2020 - Aug 31, 2020 | Jan 1, 2019 - Aug 31, 2019 |
| Gluckman 2020 | Observational (cohort) | USA | 1,915 | 13,329 | 67 years (13) vs. 68 (13) | 34% vs. 33% | Cardiovascular | Myocardial infarction | Feb 23, 2020 - May 16, 2020 | Dec 30, 2018 - Feb 22, 2020 |
| Goksoy 2020 | Observational (cohort) | Turkey | 265 | 353 | 45 years (19) vs. 46 (19) | 56% vs. 47% | Multiple illnesses (other) | Surgical complaints | Mar 15, 2020 - May 15, 2020 | Mar 15, 2019 - May 15, 2019 |
| Gramegna 2020 | Observational (cohort) | Italy | 26 | 21 | 66 years (IQR: 59-74) vs. 68 (IQR: 57-71) | 27% vs. 14% | Cardiovascular | ST-elevation myocardial infarction | Feb 21, 2020 - Apr 1, 2020 | Feb 21, 2018 - Apr 1, 2019 |
| Grewal 2020 | Observational (cohort) | USA | 66 | 88 | 63 years vs. 68 years | 47% vs. 49% | Neurological | Stroke | Mar 1, 2020 - Apr 30, 2020 | Mar 1, 2019 - Apr 30, 2019 |
| Gul 2020 | Observational (cohort) | Turkey | 35 | 114 | 41 years (12) vs. 43 (16) | NR | Renal/urological | Ureteral stones | Mar 16, 2020 - Jun 1, 2020 | Mar 16, 2019 - Jun 1, 2019 |
| Gupta 2020 | Observational (cohort) | India | 52 | 89 | 35 years (IQR: 3-81) vs. 33 (IQR: 3-87) | 14% vs. 12% | Musculoskelal/soft tissue | Open fractures and infection rates | Mar 25, 2020 - Jul 21, 2020 | Mar 25 - July 21, 2019 |
| Habonimana 2020 | Observational (cohort) | Burundi | 1,037 | 1,038 | NA | 53% vs. 48% | Multiple illnesses (other) | NR | Jan - May, 2020 | Jan - May, 2019 |
| Huang 2020 | Observational (cohort) | China | 53 | 53 | 62 years (IQR: 54-71) vs. 60 (IQR: 53-72) | 35% vs. 35% | Cardiovascular | Myocardial infarction | Feb 1, 2020 - Apr 15, 2020 | Jan 1 - Dec 31, 2019 |
| Jacob 2020 | Observational (cohort) | Australia | 97 | 126 | NR | NR | Trauma/orthopedic | Trauma | Mar 1, 2020 - Apr 30, 2020 | Mar 1, 2019 - Apr 30, 2019 |
| Jacobson 2020 | Observational (cohort) | USA | NR | NR | NR | NR | Respiratory | COVID-19 | Mar 1, 2020 - May 30, 2020 | Mar 1, 2018 - May 30, 2019 |
| Jasne 2020 | Observational (cohort) | USA | 211 | 167 | 69 years (IQR: 57-80); 71 (IQR: 61-81) | 46% vs. 56% | Neurological | Stroke code | Mar 1, 2020 - Apr 30, 2020 | February 1, 2020 |
| John 2020 | Observational (cohort) | UAE | 210 | 148 | 57 years (14) vs. 58 years (15) | 28% vs. 32% | Neurological | Stroke | Mar 1, 2020 - May 20, 2020 | Mar 1, 2019 - May 20, 2019 |
| Kastritis 2020 | Observational (cohort) | Greece | 653 | 1,133 | 55 years vs. 53 years | NR | Multiple illnesses (other) | Emergency department utilization | Mar 1 - 31, 2020 | Mar 1, 2019 - Mar 31, 2019 |
| Katsouras 2020 | Observational (cohort) | Greece | 158 | 239 | 63 years (IQR: 52-69) vs. 63 (IQR: 53-72) | 26% vs. 30% | Neurological | Stroke/acute coronary syndrome | Mar 2, 2020 - Apr 12, 2020 | Mar 2 2019 - Apr 12, 2019 |
| Keizman 2020 | Observational (cohort) | Israel | 108 | 173 | 64 years (14) vs. 64 (14) | NR | Cardiovascular | Cardiac surgery | Mar 1, 2020 - Apr 30, 2020 | Mar 1, 2019 - Apr 30, 2019 |
| Khalil 2020 | Observational (cohort) | UK | 1,718 | 1,681 | 33 years (IQR: 29-36) vs. 33 (IQR: 29-36) | 100% vs. 100% | Multiple illnesses (other) | Pregnancy/birth | Feb 1, 2020 - Jun 14, 2020 | Oct 1, 2019 - Jan 31, 2020 |
| Laskar 2020 | Observational (cohort) | UK | 174 | 277 | 58 years (22) vs. 57 (21) | 58% vs. 58% | Multiple illnesses (other) | Surgical complaints | Apr, 2020 | April, 2019 |
| Lau 2020 | Observational (cohort) | Hong Kong/China | 216 | 261 | NR | NR | Gastrointestinal | Gastrointestinal and Liver Diseases | Jan - May, 2020 | Jan - May, 2019 |
| Lauridsen 2020 | Observational (cohort) | Denmark | 60 | 342 | 69 years (IQR: 62-76) vs. 70 (IQR: 60-77) | 29% vs. 25% | Cardiovascular | Myocardial infarction | Mar 12, 2020 - May 13, 2020 | Mar 12, 2015 - May 13, 2019 |
| Leitinger 2020 | Observational (cohort) | Austria | 21 | 117 | 69 years (IQR: 22-97) vs. 71 (IQR: 20-96) | 38% vs. 58% | Neurological | Status epilepticus | Mar - Apr, 2020 | Jan 2018 - Feb 2020 |
| Lerner 2020 | Observational (cohort) | USA | NA | 37,550,949 | NR | NR | Multiple illnesses (other) | NR | Mar 2-15, 2020 | Oct 1, 2017 - May 21, 2020 |
| Leung 2020 | Observational (cohort) | Hong Kong/China | 319, 230 | 4,183 | NR | NR | Neurological | Epilepsy | Jun 18, 2009 - Augt 29, 2009 | Jan 23, 2015 - Mar 24, 2019 |
| Li 2020 | Observational (cohort) | China | 21 | 42 | 64 years (IQR: 54-72) vs. 65 (IQR: 56-74) | 29% vs. 26% | Neurological | Acute ischemic stroke | Jan 23, 2020 - Apr 8, 2020 | Feb 3, 2019 - Apr 17, 2019 |
| Little 2020 | Observational (cohort) | UK | 348 | 440 | 63 years (IQR: 55-71) vs. 63 (IQR: 55-73) | 20% vs. 22% | Cardiovascular | ST-Elevation Myocardial Infarction (STEMI) | Mar 1, 2020 - Apr 30, 2020 | Mar 1, 2019 - Apr 30, 2019 |
| Li 2020 | Observational (cohort) | Taiwan/China | 1,038 | 1,092 | NR | NR | Cardiovascular | ST-Elevation Myocardial Infarction (STEMI) | Feb 1, 2020 - Apr 30, 2020 | Feb 1, 2019 - Apr 30, 2019 |
| Luostarinen 2020 | Observational (cohort) | Finalnad | 119 | 105 | 56 years (IQR: 44-71) vs. 50 years (IQR: 62-72) | 38% vs. 42% | Trauma/orthopedic | Traumatic brain injury (TBI) and subarachnoid hemorrhage (SAH) | Jan 1, 2020 - May 31, 2020 | Jan 1, 2019 - May 31, 2019 |
| Lv 2020 | Observational (cohort) | China | 966 | 1,624 | 53 years (23) vs. 51 (22) | 44% vs. 46% | Trauma/orthopedic | Bone fracture | Jan 20, 2020 - Feb 19, 2020 | Jan 31, 2019 - Mar 2, 2019 |
| Madanelo 2020 | Observational (cohort) | Portugal | 122 | 263 | 54 years vs. 54 years | 15% vs. 33% | Renal/urological | Hematuria, renal colic, acute urinary retention, urinary track infection (UTI), post-renal acute renal injury, testicular pain, testicular torsion, balanoposthitis, perineal or scrotal abscess, lower UTI, flank pain, trauma, post-op[ wound complication, urological cancer, complications related to urinary diversions | Mar 11, 2020 - Apr 1, 2020 | Mar 11, 2019 - Apr 1, 2019 |
| Magnani 2020 | Observational (cohort) | Italy | 77,339 | NR | NR | NR | Respiratory | NA | Mar 15, 2020 - Apr 15, 2020 | Jan 1, 2015 - Apr 15, 2019 |
| Magro 2020 | Observational (cohort) | Italy | 65 | 76 | NR | NR | Infectious | TB | Mar 1, 2020 - Apr 30, 2020 | Mar 1, 2019 - Apr 30, 2019 |
| Malik-Tabassum 2020 | Observational (cohort) | UK | 68 | 174 | 84 years (9) | 63% vs. 81% | Trauma/orthopedic | Hip fracture | Mar 23, 2020 - Nov 5, 2020 | Mar 23, 2018 - Nov 5, 2019 |
| Mannucci 2020 | Observational (cohort) | Italy | 52,452,445 | NR | NR | NR | Respiratory | COVID-19 | Feb 20, 2020 - Mar 31, 2020 | Feb 20, 2015 - Mar 31, 2019 |
| Marijon 2020 | Observational (cohort) | France | 521 | 3,052 | 70 years (17) vs. 69 (18) | 36% vs. 40% | Cardiovascular | Cardiac arrest | Mar 16, 2020 - Apr 26, 2020 | Mar 16, 2012 - Apr 26, 2019 |
| Marini 2020 | Observational (cohort) | Italy | 34 | 27 | 81 years (9) vs. 80 (10) | 38% vs. 41% | Cardiovascular | Heart block/Bradycardia | Mar - Apr, 2020 | Mar - Apr, 2019 |
| Mariottini 2020 | Observational (cohort) | Finland | 4,216 | 20,840 | NR | NR | Metabolic/toxins | Detection of illegal drugs in post-mortem toxicology | Jan - Aug, 2020 | Jan 2015 - Aug 2019 |
| McGuinness 2020 | Observational (cohort) | USA | 797 | 285 | 63 years (IQR: 61-64) vs. 68 years (IQR: 66-70) | 48% vs. 40% | Respiratory | **Pandemic**: COVID-19, Barotrauma (Right or left pneumothorax, pneumomediastinum, pneumopericardium, subcutaneous emphysema), COVID-19 NEGATIVE PATIENTS: Sepsis, Non-trauma related neurologic disease, complications of gastrointestinal or genitourinary disease. **Pre-Pandemic:** Acute Respiratory Distress Syndrome | Mar 1, 2020 - Apr 6, 2020 | Feb 1, 2016 - Feb 1, 2020 |
| McLean 2020 | Observational (cohort) | UK | 106 | 207 | 58 years vs. 51 years | 63% vs. 61% | Gastrointestinal | Gastrointestinal (GI) obstruction, GI perforation; GI cancer | Mar 16, 2020 - Apr 15, 2020 | Feb 15, 2020 - Mar 15, 2020 |
| Mendlovic 2020 | Observational (cohort) | Israel | 409 | 1,671 | 75 years (18) vs. 77 (16) | 49% vs. 51% | Multiple illnesses (other) | Internal medicine | Mar 15, 2020 - Apr 30, 2020 | Mar 15, 2017 - Apr 30, 2019 |
| Mengal 2020 | Observational (cohort) | Pakistan | 1,139 | 3,072 | 56 years (12) vs. 53 (14) | 19% vs. 23% | Cardiovascular | Acute ST-Elevation Myocardial Infarction | Mar - Apr, 2020 | Mar 2019 - Feb 2020 |
| Merkler 2020 | Observational (cohort) | USA | 1,916 | 1,486 | 64 years (IQR: 51-76) vs. 62 years (IQR: 42-78) | 43% vs. 55% | Neurological | Acute Ischemic stroke | Mar 4, 2020 - May 2, 2020 | Jan 1, 2016 - May 31, 2018 |
| Mesnier 2020 | Observational (cohort) | France | 481 | 686 | 65 years (13) vs. 66 years (14) | 26% vs. 26% | Cardiovascular | ST-Elevation Myocardial Infarction, Non ST-Elevation Myocardial Infarction | Mar 15, 2020 - Apr 12, 2020 | Feb 17, 2020 - Mar 16, 2020 |
| Meyer 2020 | Observational (cohort) | Israel | 1,666 | 1,654 | 32 years (5) vs. 32 (5) | 100% | Multiple illnesses (other) | Pregnant women and neonates | Feb - Mar, 2020 | Feb - Mar, 2019 |
| Miles 2020 | Observational (cohort) | USA | 125 | 117 | 67 years (IQR: 57-76) vs. 66 (IQR: 56-77) | 34% vs. 42% | Cardiovascular | In-hospital cardiac arrest | Mar 1, 2020 - May 15, 2020 | Jan 1, 2019 - Dec 31, 2019 |
| Mitra 2020 | Observational (cohort) | Australia | 52 | 57 | 71 years (16) vs. 75 (14) | 33% vs. 16% | Cardiovascular | Stroke and Acute Myocardial Infarction | Mar 26, 2020 - Apr 23, 2020 | Mar 26, 2019 - Apr 23, 2019 |
| Mohamed 2020 | Observational (cohort) | UK | 9,063 | 264,078 | NA | NA | Cardiovascular | Stable Angina, Acute coronary syndrome | Mar - May, 2020 | Mar 2017 - Feb 2020 |
| Mohammad 2020 | Observational (cohort) | Sweden | 2,443 | 15,213 | 70 years (IQR: 61-77) vs. 70 (IQR: 61-77) | 33% vs. 33% | Cardiovascular | Myocardial Infarction | Mar 1, 2020 - May 7, 2020 | Mar 1, 2015 - May 7, 2019 |
| Monti 2020 | Observational (cohort) | Italy | 4 | 16 | 79 years (6) vs. 71 (10) | 100% vs. 81% | Multiple illnesses (other) | Giant cell arteritis | Feb 21, 2020 - Apr 30, 2020 | Feb 21, 2019 - Apr 30, 2019 |
| Mountantonakis 2020 | Observational (cohort) | USA | 8,160 | 4,326 | NR | NR | Cardiovascular | Out-of-hospital sudden cardiac arrest, Acute coronary syndrome | Mar 20, 2020 - Apr 22, 2020 | Mar 20, 2019 - Apr 22, 2019 |
| Moustakis 2020 | Observational (cohort) | South African | 202 | 567 | NON-TRAUMA: 55 years (16) vs. 53 years (17); TRAUMA: 36 years (16) vs. 33 years (12) | 13% vs. 20% | Gastrointestinal | Acute abdomen (all causes), bowel obstruction (all causes), critical and acute limb ischemia, appendicitis, upper gastrointestinal bleeding, soft-tissue infections, and management of suspected or confirmed malignancies | Mar 27, 2020 - Apr 30, 2020 | Feb 3, 2020 - Mar 26, 2020 |
| Mulholland 2020 | Observational (cohort) | Scotland | NR | NR | NR | NR | Multiple illnesses (other) | NR | Jan 5, 2020 - Jun 28, 2020 | Jan 5, 2018 - Jun 28, 2019 |
| Naccarato 2020 | Observational (cohort) | Italy | 16 | 29 | 77 years (IQR: 67-81); 78 years (IQR: 70-85) | NA | Neurological | Stroke | Mar 9, 2020 - Apr 9, 2020 | Mar 9, 2019 - Apr 9, 2019 |
| Nagamine 2020 | Observational (cohort) | USA | 48 | 64 | 65 years (16) vs. 69 years (15) | 39% vs. 25% | Neurological | Stroke | Mar 1, 2020 - Apr 30, 2020 | Mar 1, 2019 - Apr 30, 2019 |
| Nef 2020 | Observational (cohort) | Germany | 8,018 | 9,377 | NA | NA | Cardiovascular | COVID-19, cardiac death, pulmonary embolism, stroke | Mar 23, 2020 - Apr 26, 2020 | Mar 23, 2019 - Apr 26, 2019 |
| Nguyen-Huynh 2020 | Observational (cohort) | USA | 783 | 8,337 | 69 years (15) vs. 69 years (16) | 52% vs. 53% | Neurological | Stroke | Mar 15 - May 9, 2020 | Jan 1, 2019 - Mar 14, 2020 |
| Nunez 2020 | Observational (cohort) | Spain | 512 | 6,053 | 58 years (23) vs. 57 (22) | 49% vs. 52% | Trauma/orthopedic | Trauma incl. osteoporotic hip fractures | Mar 14, 2020 - Apr 2, 2020 | Mar 17, 2018 - Mar 13, 2020 |
| Ogliari 2020 | Observational (cohort) | UK | 182 | 6,499 | 67 years (IQR 58-78) vs. 67 (IQR: 57-77) | 65% vs. 67% | Trauma/orthopedic | Hip fractures | Mar 21, 2020 - Apr 4, 2020 | Mar 21, 2015 - Apr 4, 2019 |
| Okwu 2020 | Observational (cohort) | UK | 92 | 176 | NA | NA | Renal/urological | Renal calculi, hematuria, acute retention, urosepsis, cancer, testicular issues, renal failure, and others. | Mar 23, 2020 - Apr 30, 2020 | Mar 23, 2019 - Apr 30, 2019 |
| Orellana 2020 | Observational (cohort) | Brazil | 1,418 | 478 | NA | 38% vs. 44% | Cardiovascular | NA | Mar 14, 2020 - Mar 28, 2020 | Mar 14, 2018 - Mar 28, 2019 |
| Padmanabhan 2020 | Observational (cohort) | UK | 101 | 167 | 70 (15) years vs. 73 (14) | 48% vs. 49% | Neurological | Stroke or transient ischemic attack | Mar 15, 2020 - Apr 14, 2020 | Mar 15, 2019 - Apr 14, 2019 |
| Pagotto 2020 | Observational (cohort) | Brazil | 1,838 | 14,117 | NR | NR | Multiple illnesses (other) | Plastic surgeries | Mar 16, 2020 - May 24, 2020 | Jan 7, 2019 - Mar 15, 2020 |
| Papafaklis 2020 | Observational (cohort) | Greece | 771 | 1,077 | 64 years (IQR: 56-74) vs. 65 years (IQR: 56-74) | 21% vs. 24% | Cardiovascular | Acute coronary syndrome (ST-segment elevation myocardial infarction [STEMI], non-STEMI [NSTEMI], and unstable angina [UA]) | Mar 2, 2020 - Apr 12, 2020 | Mar 2, 2019 - Apr 12, 2019 |
| Patel 2020 | Case-series (with control group) | USA | 82 | 214 | 59 years (13) vs. 59 years (14) | 32% vs. 38% | Multiple illnesses (other) | Acute, primary rhegmatogenous retinal detachment (RRD) | Mar 9, 2020 - Apr 27, 2020 | Mar 9, 2018 - Apr 27, 2019 |
| Patel 2020 | Observational (cohort) | UK | 75 | 151 | 47 years (IQR: 32-63) vs. 47 years (IQR: 34-67) | 45% vs. 52% | Multiple illnesses (other) | NA | Mar 30, 2020 - Apr 12, 2020 | Mar 30, 2019 - Apr 12, 2019 |
| Pathare 2020 | Observational (cohort) | India | 220 | 369 | NA | 29% vs. 42% | Cardiovascular | NA | Mar 24, 2020 - May 3, 2020 | March 24 - May 3, 2019 |
| Patt 2020 | Observational (cohort) | USA | NA | NA | NR | NR | Multiple illnesses (other) | Cancer | Mar - Jul 2020 | Mar - Jul 2019 |
| Perkin 2020 | Case-series (with control group) | UK | 243 | 194 | 76 years vs. 78 years (median) | 38% vs. 50% | Multiple illnesses (other) | NA | Mar 12, 2020 - Apr 23, 2020 | Mar 12, 2019 - Apr 23, 2019 |
| Piccininni 2020 | Observational (cohort) | Italy | 166 | 950 | 75 years (IQR: 65-74) | NR | Multiple illnesses (other) | NA | February 21, 2020 - April 11, 2020 | Jan 2012 - Feb 2020 |
| Pintado 2020 | Observational (cohort) | Peru | 2,213 | 450 | 58 years vs. 61 years (median) | 54% vs. 57% | Trauma/orthopedic | All types of orthopedic trauma | Mar 16, 2020 - Apr 15, 2020 | Feb 16, 2020 - Mar 15, 2020 |
| Popovic 2020 | Observational (cohort) | France | 83 | 1,552 | 60 years (12) vs. 63 (13) | 24% vs. 44% | Cardiovascular | ST elevation myocardial infarction (STEMI) patients who underwent percutaneous coronary intervention | Feb-May, 2020 | Feb 2008 - May 2017 |
| Pop 2020 | Observational (cohort) | France | 174 | 288 | NR | NR | Neurological | Stroke | Mar 1, 2020 - Mar 31, 2020 | Mar 1, 2019 - Mar 31, 2019 |
| Quaquarini 2020 | Observational (cohort) | Italy | 469 | 2,443 | 69 years (IQR: 34-90) | 68% | Multiple illnesses (other) | Cancer  Breast cancer was the most common diagnosis (44%), followed by colorectal cancer (17%), prostate cancer (8%), kidney cancer (8%), lung cancer (6%), pancreatic cancer (4%), gynecological (3%), bladder and upper gastrointestinal cancer (3%), and others (4%) | Feb 24, 2020 - Apr 30, 2020 | Feb 24, 2016 - Apr 30, 2019 |
| RashidHons 2020 | Observational (cohort) | UK | 524 | 731 | 67 years vs. 63 years | 29% vs. 21% | Cardiovascular | Out of hospital cardiac arrest (OHCA) - STEMI and NSTEMI | Feb 1, 2020 - May 14, 2020 | Feb 1, 2019 - May 14, 2019 |
| Rebecchi 2020 | Observational (cohort) | Italy | 65 | 60 | 68 years (7) vs 66 (4) | NR | Gastrointestinal | Esophageal malignancies | Mar 9, 2020 - May 3, 2020 | Mar 9, 2019 - May 3, 2019 |
| Richter 2020 | Observational (cohort) | Germany | 47,983 | 119,657 | 74 years (19) vs. 75 (19) | 47% vs. 47% | Neurological | Acute ischemic stroke, transient ischemic attack, intracerebral hemorrhage | Jan 16, 2020 - Mar 15, 2020 | Mar 15, 2019 - Mar 15, 2020 |
| Riemann 2020 | Observational (cohort) | Germany | 1,080 | 1,080 | 49 years (IQR: 47-51) vs. 45 (IQR 44-47) | 40%, 39% | Head and neck | Suspected malignancies, head and neck tumor operation, acute bleeding/inflammation, acute mastoiditis | Mar 16, 2020 - May 10, 2020 | Jan 20, 2020 - Mar 15, 2020 |
| Rodriguez-Leor 2020 | Observational (cohort) | Spain | 1,113 | 1,503 | 63 years (13) vs. 64 (13) | 22% vs. 22% | Cardiovascular | ST Elevated myocardial infarction (STEMI) | Mar 16, 2020 - Apr 14, 2020 | Apr 1, 2019 - Apr 30, 2019 |
| Rupa 2020 | Observational (cohort) | Germany | 4,753 | 4,922 | NR | NR | Neurological | Neurosurgical procedure (cranial, spinal, peripheral nerve) study breaks it down by broad groups like aphasia, hydrocephalus, lower back pain etc. | Jan 1, 2020 - Jun 30, 2020 | Jan 1, 2019 - June 30, 2019 |
| Russo 2020 | Observational (cohort) | Italy | 97 | 107 | 79 years (IQR:74-83) vs. 77 years (IQR: 72-85] | 44% vs. 38% | Cardiovascular | Arrhythmogenic syncope leading to cardiac rhythm management procedures | Mar 10, 2020 - May 4, 2020 | Mar 10, 2019 - May 4, 2019 |
| Salarifar 2020 | Observational (cohort) | Iran | 178 | 146 | 59 years (12) vs. 60 (11) | 23% vs. 22% | Cardiovascular | ST-Elevation Myocardial Infarction (STEMI) | Feb 29, 2020 - Apr 30, 2020 | Mar 1, 2019 - Apr 30, 2019 |
| Scholz 2020 | Observational (cohort) | Germany | 387 | 1,329 | 64 years (0.4) vs. 65 (0.7) | 72% vs. 72% | Cardiovascular | ST elevation myocardial infarct (STEMI) | March 1, 2020 | March 2017-2019 |
| Scortichini 2020 | Observational (cohort) | Italy | 208,320 | 3,299,674 | NR | NR | Infectious | COVID 19 | Feb 2020 - May 2020 | Jan 2015 - Feb 2020 |
| Secco 2020 | Observational (cohort) | Italy | 162 | 84 | 68 years (30) vs. 70 (33) | 30% vs. 26% | Cardiovascular | acute coronary syndrome (ACS) (subgroups: ST elevation myocardial infarction [STEMI], non-STEMI [NSTEMI], other ACS) | Mar, 2020 | Mar, 2019 |
| Seiffert 2020 | Observational (cohort) | Germany | 31,602 | 35,841 | NR | NR | Cardiovascular | Stroke/transient ischemic stroke, myocardial infarction, Aortic rupture, acute limb ischemia | Jan - May, 2020 | Jan-May, 2019 |
| Sharma 2020 | Observational (cohort) | USA | 582 | 971 | NR | NR | Neurological | Stroke/transient ischemic attack | Mar-Apr, 2020 | Mar-Apr, 2019 |
| Silva 2020 | Observational (cohort) | Brazil | NR | NR | NR | NR | Multiple illnesses (other) | NA | Mar-May, 2020 | Mar-May, 2019 |
| Sinnathamby 2020 | Observational (cohort) | UK | NR | NR | NR | NR | Multiple illnesses (other) | NA | Jan-May, 2020 | Jan-May, 2019 |
| Slullitel 2020 | Observational (case-control) | Argentina | 74 | 86 | 86 year (IQR: 80-91) vs. 86 (IQR: 78-90) | 88% vs. 78% | Trauma/orthopedic | Hip fractures | May, 2020 | May, 2019 |
| Sobti 2020 | Observational (cohort) | UK | 94 | 188 | 84 years vs. 82 years | NR | Trauma/orthopedic | neck of femur fracture requiring surgery | Mar-May, 2020 | Mar 2019 - Feb 2020 |
| Stang 2020 | Observational (cohort) | Germany | NR | NR | NR | NR | Multiple illnesses (other) | all-cause mortality | Mar-Jun, 2020 | Mar-June, 2016-2019 |
| Stohr 2020 | Observational (cohort) | Germany | 5,799 | 5,799 | 71 years (15) | 50% | Cardiovascular | Unstable angina, uncontrolled hypertension, stroke, chronic obstructive pulmonary diseases (COPD) exacerbation, arrhythmia, heart failure, dizziness/syncope, ST elevation myocardial infarction (STEMI), cardiopulmonary resuscitation | Jan-Apr, 2020 | Jan 2019 - Apr 2019 |
| Stokes 2020 | Observational (cohort) | USA | NR | NR | NR | NR | Infectious | COVID-19 mortality and all-cause mortality | Jan - Dec, 2020 | Jan 2013 - Dec 2018 |
| Strang 2020 | Observational (cohort) | Sweden | 1,346 | 33,451 | 85 years (9) vs. 85 (10) | 49% vs. 56% | Infectious | Covid-19 palliative care and all other palliative care | Jan - May, 2020 | Jan-Dec 2019 |
| Strang 2020 | Observational (cohort) | Sweden | 6,613 | 16,548 | 80 years vs. 79 years | 50%% vs. 52% | Infectious | Covid-19 death and all-cause death | Mar - May, 2020 | Mar-May, 2016-2019 |
| Strauss 2020 | Observational (cohort) | USA | NA | NA | NR | NR | Transplant | Liver transplants | Mar - Aug, 2020 | Feb 2016 - Jan 2020 |
| Tanacan 2020 | Observational (cohort) | Turkey | 717 | 1,165 | NA | 65% vs. 66% | Infectious | Dermatological diseases | Mar - May, 2020 | Mar-May 2019 |
| Teo 2020 | Observational (cohort) | Hong Kong/China | 73 | 89 | 70 years (16) vs. 74 (13) | 68% vs. 55% | Neurological | Transient ischemic attack (TIA)/stroke | Jan 23, 2020 - Mar 24, 2020 | Jan 23, 2019 - Mar 24, 2019 |
| Thakrar 2020 | Observational (cohort) | UK | 43 | 154 | 82 years (11) vs. 82 years (12) vs. 84 years (9) vs. 82 years (11) | 20% vs. 38% vs. 36% vs. 40% | Trauma/orthopedic | Hip fracture | Mar 15, 2020 - Apr 13, 2020 | Mar 15, 2018 - Mar 14, 2020) |
| Tomasoni 2020 | Observational (cohort) | Italy | 34 | 51 | 66 years (12) vs 65 years (13) | 75% vs. 62% | Cardiovascular | ST-elevation myocardial infarction (STEMI) | Feb 21, 2020 - Apr 10, 2020 | Jan 3, 2020 - Feb 20, 2020 |
| Toner 2020 | Observational (cohort) | Australia | 32 | 217 | 80 years (14) vs. 80 years (11) | 56% vs. 48% | Cardiovascular | Heart failure | Mar 16, 2020 - Apr 14, 2020 | Mar 16, 2014 - Apr 14, 2017 |
| Tousek 2020 | Observational (cohort) | Czech Republic | 181 | 834 | 65 years (12) vs. 66 (13) | 30% vs. 35% | Cardiovascular | Acute Coronary Syndrome | Feb 1, 2020 - May 30, 2020 | Oct 1, 2018 - Jan 31, 2020 |
| Trabattoni 2020 | Observational (cohort) | Italy | 46 | 19 | NR | NR | Cardiovascular | ST-elevation myocardial infarction (STEMI) and non-STEMI (NSTEMI) | Mar 8, 2020 - Apr 10, 2020 | Mar 8, 2019 - Apr 10, 2019 |
| Uchino 2020 | Observational (cohort) | USA | 188 | 717 | NR | NR | Neurological | Stroke | Mar 9, 2020 - Apr 2, 2020 | Jan 1, 2020 - Mar 8, 2020 |
| Vandoros 2020 | Observational (cohort) | UK | 11,762 | 36,964 | NR | 50% vs. 48% | Multiple illnesses (other) | Mortality not reported as COVID-19 | Mar 7, 2020 - May 12, 2020 | Mar 7, 2015 - May 12, 2019 |
| Vanni 2020 | Observational (cohort) | Italy | 318 | 3,617 | NR | NR | Multiple illnesses (other) | Surgical needs for injury, abdominal disease, genitourinary disease, vascular disease, neurosurgical disease, thoracic disease, other | Mar 11, 2020 - May 3, 2020 | Mar 11, 2019 - Mar 10, 2020 |
| Vestergaard 2020 | Observational (cohort) | Multinational (24 European countries) | 185,287 | 278,782 | NA | NR | Multiple illnesses (other) | All-cause mortality | Jan 1, 2020 - May 7, 2020 | Jan 1, 2016 - May 7, 2019 |
| Vieira 2020 | Observational (cohort) | Portugal | 10,445 | 9,190 | NA | NR | Multiple illnesses (other) | All-cause mortality | Mar 16, 2020 - Apr 14, 2020 | Mar 16, 2010 - Apr 14, 2019 |
| Wang 2020 | Observational (cohort) | USA | 255 | 320 | 73 years (62-83) vs. 70 years (62-81), p = 0.513 | 47% vs. 48% | Neurological | Acute ischemic stroke | Mar 12, 2020 - Jun 30, 2020 | Dec 1, 2019 - Mar 11, 2020 |
| Weinberger 2020 | Observational (cohort) | USA | 780,975 | 658,700 | NA | NA | Multiple illnesses (other) | All-cause mortality | Mar 1, 2020 - May 30, 2020 | Jan 5, 2015 - Jan 25, 2020 |
| Westgard 2020 | Observational (cohort) | USA | 4,666 | 20,284 | 42 years (IQR: 29-60) vs. 44 years (IQR: 28-59) | 47% vs. 51% | Multiple illnesses (other) | Emergency department (ED) visits | Mar 14, 2020 - Apr 10, 2020 | Feb 16, 2019 - Mar 13, 2020 |
| Wong 2020 | Observational (cohort) | Hong Kong/China | 127,604 | 800,674 | NA | NR | Trauma/orthopedic | Trauma fractures and dislocations (upper-limb and lower-limb fractures, hip fractures, pelvic fractures, joint dislocations, spinal fractures), spinal pathologies (spinal cord compression, spinal cord injury, central cord syndrome), infections (necrotizing fasciitis, septic arthritis, osteomyelitis), bone and soft-tissue malignancies, elective reconstructive operations (total hip arthroplasty, total knee arthroplasty, anterior cruciate ligament reconstruction) | Jan 25, 2020 - Mar 27, 2020 | Jan 25, 2016 Mar 28, 2019 |
| Woolf 2020 | Observational (cohort) | USA | 505,059 | 419,058 | NR | NR | Multiple illnesses (other) | All-cause mortality | Mar 1, 2020 - Apr 25, 2020 | Dec 29, 2013 - Feb 29, 2020 |
| Yalamanchi 2020 | Observational (cohort) | India | 216 | 629 | 59 years (14) vs. 63 years (12) vs. 62 years (13) | NR | Cardiovascular | Acute Coronary Syndrome, acute decompensated heart failure, acute pulmonary embolism, others (hypertensive emergencies, metabolic abnormalities, etc.) | Mar 22, 2020 - Aug 1, 2020 | Mar 22, 2018 - Aug 1, 2019 |
| Zhang 2020 | Observational (cohort) | China | 119 | 276 | NR | NR | Cardiovascular | ST-elevation myocardial infarction (STEMI) and non-STEMI (NSTEMI) | Jan 1, 2020 - Mar 31, 2020 | Jan 1, 2018 - Mar 31, 2019 |

**ACS:** acute coronary syndrome, **COPES**: Coronavirus Disease (COVID-19) and Outcomes Associated with Pandemic Effects Study (COPES), **COVID-19**: Coronavirus Disease-2019, **ED:** emergency department; **IQR**: inter-quartile range, **NA:** not applicable, **NR**: not reported, **SD**: standard deviation
