# Supplementary material for: Non-COVID outcomes associated with the coronavirus disease-2019 (COVID-19) pandemic effects study (COPES): A systematic review and meta-analysis
Source: PLoS One. 2022 Jun 24;17(6):e0269871. doi: 10.1371/journal.pone.0269871 (PMC9231780; doi:10.1371/journal.pone.0269871)
Supplement: S4 Table — (DOCX) [file pone.0269871.s005.docx]

**S4 Table: Subgroups**

| **Study Author (year)** | **Risk of bias** | **Income country** | **Level of intervention** | **Case-mix** |
| --- | --- | --- | --- | --- |
| Abdelaziz 2020 | High | High | Acute care hospital | Medical |
| Agarwal 2020 | High | Low/middle | Acute care hospital | Mixed |
| Agarwal 2020 | Low | High | Acute care hospital | Medical |
| Aldujeli 2020 | Low | Low/middle | Acute care hospital | Medical |
| Amaddeo 2020 | Low | High | Acute care hospital | Mixed |
| Amoo 2020 | High | High | Acute care hospital | Surgical |
| Amukotuwa 2020 | High | High | Acute care hospital | Medical |
| Andersson 2020 | Low | High | Acute care hospital | Medical |
| Anteby 2020 | High | High | Acute care hospital | Surgical |
| Arafa 2020 | High | High | Acute care hospital | Surgical |
| Athiel 2020 | High | High | Acute care hospital | Surgical |
| Aviran 2020 | High | High | Acute care hospital | Surgical |
| Baert 2020 | High | High | Acute care hospital | Medical |
| Bajunaid 2020 | Low | High | Acute care hospital | Surgical |
| Ball 2020 | High | High | Acute care hospital | Medical |
| Barten 2020 | High | High | Acute care hospital | Medical |
| Batra 2020 | High | High | Acute care hospital | Surgical |
| Becq 2020 | High | High | Jurisdiction | Mixed |
| Benites-Goni 2020 | High | High | Acute care hospital | Mixed |
| Bhatt 2020 | High | High | Acute care hospital | Mixed |
| Bilinski 2020 | High | High | Acute care hospital | Mixed |
| Birkmeyer 2020 | Low | High | Acute care hospital | Mixed |
| Blangiardo 2020 | High | High | Acute care hospital | Mixed |
| Boyarsky 2020 | High | High | Acute care hospital | Surgical |
| Bromage 2020 | High | High | Acute care hospital | Medical |
| Bugger 2020 | High | High | Acute care hospital | Mixed |
| BustosSierra 2020 | High | High | Acute care hospital | Mixed |
| Butt 2020 | High | High | Acute care hospital | Mixed |
| Butt 2020 | High | High | Acute care hospital | Mixed |
| Calderon-Larranaga 2020 | Low | High | Acute care hospital | Mixed |
| Cannata 2020 | High | High | Acute care hospital | Mixed |
| Cannavo 2020 | High | High | Acute care hospital | Surgical |
| Cano-Valderrama 2020 | High | High | Acute care hospital | Surgical |
| Casalino 2020 | High | High | Acute care hospital | Mixed |
| Cates 2020 | High | High | Acute care hospital | Mixed |
| Cevallos-Valdiviezo 2020 | High | Low/middle | Acute care hospital | Mixed |
| Chan 2020 | Low | High | Acute care hospital | Medical |
| Chan 2020 | High | High | Acute care hospital | Medical |
| Claeys 2020 | High | High | Acute care hospital | Medical |
| D'Apolito 2020 | Low | High | Jurisdiction | Surgical |
| Davies 2020 | High | High | Jurisdiction | Surgical |
| Dawoud 2020 | High | High | Jurisdiction | Surgical |
| Dayananda 2020 | High | High | Jurisdiction | Surgical |
| deHavenon 2020 | High | High | Jurisdiction | Medical |
| Dell'Utri 2020 | Low | High | Jurisdiction | Mixed |
| DeLuca 2020 | High | High | Jurisdiction | Medical |
| DeRosa 2020 | High | High | Jurisdiction | Medical |
| D'Urbano 2020 | High | High | Acute care hospital | Surgical |
| Egol 2020 | High | High | Acute care hospital | Surgical |
| Eshraghian 2020 | High | High | Acute care hospital | Mixed |
| Fadel 2020 | Low | High | Acute care hospital | Medical |
| Frankfurter 2020 | High | High | Acute care hospital | Medical |
| Friedman 2020 | High | Low/middle | Acute care hospital | Medical |
| Giannouchos 2020 | Low | High | Acute care hospital | Mixed |
| Gluckman 2020 | High | High | Acute care hospital | Medical |
| Goksoy 2020 | High | High | Acute care hospital | Surgical |
| Gramegna 2020 | Low | High | Acute care hospital | Medical |
| Grewal 2020 | High | High | Acute care hospital | Medical |
| Gul 2020 | High | Low/middle | Acute care hospital | Surgical |
| Gupta 2020 | Low | Low/middle | Acute care hospital | Surgical |
| Habonimana 2020 | High | Low/middle | Acute care hospital | Mixed |
| Huang 2020 | High | High | Acute care hospital | Medical |
| Jacob 2020 | High | Low/middle | Acute care hospital | Medical |
| Jacobson 2020 | High | High | Jurisdiction | Mixed |
| Jasne 2020 | High | High | Acute care hospital | Mixed |
| John 2020 | High | High | Acute care hospital | Mixed |
| Kastritis 2020 | High | High | Acute care hospital | Mixed |
| Katsouras 2020 | High | High | Acute care hospital | Medical |
| Keizman 2020 | High | High | Acute care hospital | Surgical |
| Khalil 2020 | Low | High | Acute care hospital | Surgical |
| Laskar 2020 | High | High | Acute care hospital | Surgical |
| Lau 2020 | Low | High | Acute care hospital | Mixed |
| Lauridsen 2020 | High | High | Acute care hospital | Mixed |
| Leitinger 2020 | High | High | Acute care hospital | Medical |
| Lerner 2020 | High | High | Acute care hospital | Medical |
| Leung 2020 | High | High | Jurisdiction | Mixed |
| Li 2020 | High | Low/middle | Jurisdiction | Medical |
| Little 2020 | High | High | Acute care hospital | Medical |
| Li 2020 | High | High | Acute care hospital | Mixed |
| Luostarinen 2020 | High | High | Acute care hospital | Mixed |
| Lv 2020 | High | High | Acute care hospital | Surgical |
| Madanelo 2020 | High | Low/middle | Acute care hospital | Mixed |
| Magnani 2020 | Low | High | Acute care hospital | Mixed |
| Magro 2020 | High | High | Jurisdiction | Mixed |
| Malik-Tabassum 2020 | High | High | Acute care hospital | Medical |
| Mannucci 2020 | Low | High | Acute care hospital | Mixed |
| Marijon 2020 | High | High | Acute care hospital | Medical |
| Marini 2020 | High | High | Acute care hospital | Mixed |
| Mariottini 2020 | High | High | Acute care hospital | Surgical |
| McGuinness 2020 | Low | High | Acute care hospital | Medical |
| McLean 2020 | High | High | Acute care hospital | Surgical |
| Mendlovic 2020 | High | High | Acute care hospital | Medical |
| Mengal 2020 | Low | Low/middle | Acute care hospital | Medical |
| Merkler 2020 | High | High | Acute care hospital | Medical |
| Mesnier 2020 | High | High | Acute care hospital | Medical |
| Meyer 2020 | High | High | Acute care hospital | Medical |
| Miles 2020 | High | High | Acute care hospital | Medical |
| Mitra 2020 | Low | High | Acute care hospital | Medical |
| Mohamed 2020 | High | High | Acute care hospital | Medical |
| Mohammad 2020 | High | High | Acute care hospital | Medical |
| Monti 2020 | High | High | Acute care hospital | Medical |
| Mountantonakis 2020 | High | High | Acute care hospital | Medical |
| Moustakis 2020 | High | Low/middle | Acute care hospital | Surgical |
| Mulholland 2020 | High | High | Acute care hospital | Mixed |
| Naccarato 2020 | High | High | Acute care hospital | Medical |
| Nagamine 2020 | High | High | Acute care hospital | Mixed |
| Nef 2020 | High | High | Jurisdiction | Mixed |
| Nguyen-Huynh 2020 | High | High | Jurisdiction | Mixed |
| Nunez 2020 | High | High | Acute care hospital | Mixed |
| Ogliari 2020 | High | High | Jurisdiction | Mixed |
| Okwu 2020 | High | High | Acute care hospital | Surgical |
| Orellana 2020 | High | Low/middle | Jurisdiction | Mixed |
| Padmanabhan 2020 | High | High | Acute care hospital | Mixed |
| Pagotto 2020 | High | Low/middle | Acute care hospital | Surgical |
| Papafaklis 2020 | High | High | Jurisdiction | Mixed |
| Patel 2020 | High | High | Acute care hospital | Surgical |
| Patel 2020 | High | High | Acute care hospital | Surgical |
| Pathare 2020 | High | Low/middle | Jurisdiction | Mixed |
| Patt 2020 | High | High | Jurisdiction | Mixed |
| Perkin 2020 | High | High | Acute care hospital | Mixed |
| Piccininni 2020 | High | High | Jurisdiction | Mixed |
| Pintado 2020 | High | Low/middle | Acute care hospital | Surgical |
| Popovic 2020 | Low | High | Acute care hospital | Medical |
| Pop 2020 | High | High | Acute care hospital | Medical |
| Quaquarini 2020 | High | High | Acute care hospital | Mixed |
| RashidHons 2020 | High | High | Acute care hospital | Medical |
| Rebecchi 2020 | Low | High | Acute care hospital | Mixed |
| Richter 2020 | High | High | Jurisdiction | Mixed |
| Riemann 2020 | High | High | Jurisdiction | Surgical |
| Rodriguez-Leor 2020 | High | High | Acute care hospital | Mixed |
| Rupa 2020 | High | High | Acute care hospital | Surgical |
| Russo 2020 | High | High | Jurisdiction | Mixed |
| Salarifar 2020 | High | High | Acute care hospital | Surgical |
| Scholz 2020 | High | High | Acute care hospital | Medical |
| Scortichini 2020 | High | High | Acute care hospital | Medical |
| Secco 2020 | High | High | Acute care hospital | Medical |
| Seiffert 2020 | High | High | Acute care hospital | Medical |
| Sharma 2020 | High | High | Acute care hospital | Medical |
| Silva 2020 | High | Low/middle | Acute care hospital | Medical |
| Sinnathamby 2020 | High | High | Jurisdiction | Medical |
| Slullitel 2020 | High | Low/middle | Acute care hospital | Surgical |
| Sobti 2020 | High | Low/middle | Acute care hospital | Surgical |
| Stang 2020 | High | High | Jurisdiction | Mixed |
| Stohr 2020 | High | Low/middle | Acute care hospital | Medical |
| Stokes 2020 | High | High | Acute care hospital | Medical |
| Strang 2020 | High | High | Jurisdiction | Medical |
| Strang 2020 | High | High | Acute care hospital | Medical |
| Strauss 2020 | Low | High | Acute care hospital | Surgical |
| Tanacan 2020 | High | Low/middle | Acute care hospital | Medical |
| Teo 2020 | High | High | Jurisdiction | Mixed |
| Thakrar 2020 | High | High | Acute care hospital | Surgical |
| Tomasoni 2020 | High | High | Acute care hospital | Surgical |
| Toner 2020 | High | High | Acute care hospital | Medical |
| Tousek 2020 | High | High | Acute care hospital | Mixed |
| Trabattoni 2020 | High | High | Acute care hospital | Mixed |
| Uchino 2020 | High | High | Acute care hospital | Mixed |
| Vandoros 2020 | High | High | Jurisdiction | Mixed |
| Vanni 2020 | High | High | Acute care hospital | Surgical |
| Vestergaard 2020 | High | High | Jurisdiction | Mixed |
| Vieira 2020 | High | High | Jurisdiction | Mixed |
| Wang 2020 | Low | High | Acute care hospital | Mixed |
| Weinberger 2020 | High | High | Jurisdiction | Mixed |
| Westgard 2020 | High | High | Acute care hospital | Mixed |
| Wong 2020 | High | High | Acute care hospital | Mixed |
| Woolf 2020 | High | High | Jurisdiction | Mixed |
| Yalamanchi 2020 | High | High | Acute care hospital | Mixed |
| Zhang 2020 | High | High | Acute care hospital | Mixed |

**COPES**: Coronavirus Disease (COVID-19) and Outcomes Associated with Pandemic Effects Study (COPES), **COVID-19**: Coronavirus Disease-2019, **NA:** not applicable, **NR**: not reported, **Y**: Yes
