# Supplementary material for: Non-COVID outcomes associated with the coronavirus disease-2019 (COVID-19) pandemic effects study (COPES): A systematic review and meta-analysis
Source: PLoS One. 2022 Jun 24;17(6):e0269871. doi: 10.1371/journal.pone.0269871 (PMC9231780; doi:10.1371/journal.pone.0269871)
Supplement: S5 Table — (DOCX) [file pone.0269871.s006.docx]

**S5A Table: Risk of Bias Assessment for Observational Cohort Studies – Newcastle-Ottawa Score**

| Studies: | Selection  (max 4 stars) | Comparability  (max 2 stars) | Outcome  (max 3 stars) | Total  (max 9 stars) | Study Quality  Grade |
| --- | --- | --- | --- | --- | --- |
| Abdelaziz 2020 | 4 | 0 | 2 | 6 | Poor |
| Agarwal 2020 | 3 | 0 | 1 | 4 | Poor |
| Agarwal 2020 | 4 | 2 | 3 | 9 | Good |
| Aldujeli 2020 | 3 | 1 | 3 | 7 | Good |
| Amaddeo 2020 | 4 | 2 | 3 | 9 | Good |
| Amoo 2020 | 3 | 1 | 1 | 5 | Poor |
| Amukotuwa 2020 | 3 | 1 | 1 | 5 | Poor |
| Andersson 2020 | 4 | 2 | 3 | 9 | Good |
| Anteby 2020 | 3 | 1 | 1 | 5 | Poor |
| Arafa 2020 | 3 | 1 | 1 | 5 | Poor |
| Athiel 2020 | 4 | 0 | 1 | 5 | Poor |
| Aviran 2020 | 4 | 0 | 3 | 7 | Poor |
| Baert 2020 | 4 | 0 | 3 | 7 | Poor |
| Ball 2020 | 4 | 2 | 3 | 9 | Good |
| Barten 2020 | 4 | 0 | 3 | 7 | Poor |
| Batra 2020 | 3 | 0 | 2 | 5 | Poor |
| Becq 2020 | 4 | 0 | 1 | 5 | Poor |
| Benites-Goni 2020 | 4 | 0 | 3 | 7 | Poor |
| Bhatt 2020 | 4 | 0 | 2 | 6 | Poor |
| Bilinski 2020 | 4 | 0 | 2 | 6 | Poor |
| Birkmeyer 2020 | 4 | 1 | 1 | 6 | Poor |
| Blangiardo 2020 | 4 | 1 | 2 | 7 | Good |
| Boyarsky 2020 | 4 | 1 | 1 | 6 | Poor |
| Bromage 2020 | 4 | 0 | 1 | 5 | Poor |
| Bugger 2020 | 4 | 0 | 1 | 5 | Poor |
| BustosSierra 2020 | 4 | 0 | 1 | 5 | Poor |
| Butt 2020 | 4 | 0 | 1 | 5 | Poor |
| Butt 2020 | 4 | 0 | 1 | 5 | Poor |
| Calderon-Larranaga 2020 | 4 | 0 | 1 | 5 | Poor |
| Cannata 2020 | 4 | 1 | 2 | 7 | Good |
| Cannavo 2020 | 4 | 0 | 1 | 5 | Poor |
| Cano-Valderrama 2020 | 4 | 1 | 1 | 6 | Poor |
| Casalino 2020 | 4 | 1 | 1 | 6 | Poor |
| Cates 2020 | 4 | 1 | 1 | 6 | Poor |
| Cevallos-Valdiviezo 2020 | 4 | 0 | 1 | 5 | Poor |
| Chan 2020 | 4 | 0 | 1 | 5 | Poor |
| Chan 2020 | 4 | 2 | 3 | 9 | Good |
| Claeys 2020 | 4 | 0 | 1 | 5 | Poor |
| D'Apolito 2020 | 4 | 0 | 1 | 5 | Poor |
| Davies 2020 | 4 | 1 | 3 | 8 | Good |
| Dawoud 2020 | 4 | 0 | 1 | 5 | Poor |
| Dayananda 2020 | 4 | 0 | 1 | 5 | Poor |
| deHavenon 2020 | 4 | 0 | 1 | 5 | Poor |
| Dell'Utri 2020 | 4 | 0 | 1 | 5 | Poor |
| DeLuca 2020 | 4 | 2 | 3 | 9 | Good |
| DeRosa 2020 | 4 | 0 | 1 | 5 | Poor |
| D'Urbano 2020 | 4 | 0 | 1 | 5 | Poor |
| Egol 2020 | 4 | 0 | 1 | 5 | Poor |
| Eshraghian 2020 | 4 | 0 | 1 | 5 | Poor |
| Fadel 2020 | 4 | 0 | 1 | 5 | Poor |
| Frankfurter 2020 | 4 | 2 | 3 | 9 | Good |
| Friedman 2020 | 4 | 0 | 3 | 7 | Poor |
| Ghanchi 2020 | 4 | 0 | 1 | 5 | Poor |
| Giannouchos 2020 | 4 | 0 | 1 | 5 | Poor |
| Gluckman 2020 | 4 | 2 | 3 | 9 | Good |
| Goksoy 2020 | 4 | 0 | 3 | 7 | Poor |
| Gramegna 2020 | 4 | 0 | 3 | 7 | Poor |
| Grewal 2020 | 4 | 2 | 3 | 9 | Good |
| Gul 2020 | 4 | 0 | 3 | 7 | Poor |
| Gupta 2020 | 4 | 0 | 3 | 7 | Poor |
| Habonimana 2020 | 4 | 2 | 3 | 9 | Good |
| Huang 2020 | 4 | 0 | 3 | 7 | Poor |
| Jacob 2020 | 3 | 0 | 3 | 6 | Poor |
| Jacobson 2020 | 4 | 0 | 3 | 7 | Poor |
| Jasne 2020 | 3 | 0 | 3 | 6 | Poor |
| John 2020 | 4 | 0 | 3 | 7 | Poor |
| Kastritis 2020 | 4 | 0 | 1 | 5 | Poor |
| Katsouras 2020 | 4 | 0 | 1 | 5 | Poor |
| Keizman 2020 | 4 | 0 | 3 | 7 | Poor |
| Khalil 2020 | 4 | 0 | 3 | 7 | Poor |
| Laskar 2020 | 4 | 2 | 3 | 9 | Good |
| Lau 2020 | 4 | 0 | 1 | 5 | Poor |
| Lauridsen 2020 | 4 | 2 | 3 | 9 | Good |
| Leitinger 2020 | 4 | 0 | 1 | 5 | Poor |
| Lerner 2020 | 4 | 0 | 3 | 7 | Poor |
| Leung 2020 | 4 | 0 | 3 | 7 | Poor |
| Li 2020 | 4 | 0 | 1 | 5 | Poor |
| Little 2020 | 4 | 0 | 1 | 5 | Poor |
| Li 2020 | 4 | 0 | 1 | 5 | Poor |
| Luostarinen 2020 | 4 | 2 | 1 | 7 | Poor |
| Lv 2020 | 4 | 0 | 1 | 5 | Poor |
| Madanelo 2020 | 4 | 0 | 1 | 5 | Poor |
| Magnani 2020 | 4 | 1 | 3 | 8 | Poor |
| Magro 2020 | 4 | 0 | 1 | 5 | Good |
| Malik-Tabassum 2020 | 4 | 0 | 3 | 7 | Poor |
| Mannucci 2020 | 4 | 1 | 3 | 8 | Poor |
| Marijon 2020 | 4 | 1 | 1 | 6 | Good |
| Marini 2020 | 4 | 0 | 3 | 7 | Poor |
| Mariottini 2020 | 4 | 0 | 2 | 6 | Poor |
| McGuinness 2020 | 4 | 1 | 1 | 6 | Poor |
| McLean 2020 | 4 | 2 | 3 | 9 | Good |
| Mendlovic 2020 | 4 | 1 | 1 | 6 | Poor |
| Mengal 2020 | 4 | 0 | 3 | 7 | Poor |
| Merkler 2020 | 4 | 1 | 3 | 8 | Good |
| Mesnier 2020 | 4 | 0 | 3 | 7 | Poor |
| Meyer 2020 | 4 | 0 | 1 | 5 | Poor |
| Miles 2020 | 4 | 0 | 1 | 5 | Poor |
| Mitra 2020 | 4 | 0 | 1 | 5 | Poor |
| Mohamed 2020 | 4 | 1 | 3 | 8 | Good |
| Mohammad 2020 | 4 | 1 | 1 | 6 | Poor |
| Monti 2020 | 4 | 0 | 1 | 5 | Poor |
| Mountantonakis 2020 | 4 | 0 | 1 | 5 | Poor |
| Moustakis 2020 | 4 | 0 | 1 | 5 | Poor |
| Mulholland 2020 | 4 | 0 | 1 | 5 | Poor |
| Naccarato 2020 | 4 | 0 | 1 | 5 | Poor |
| Nagamine 2020 | 4 | 0 | 1 | 5 | Poor |
| Nef 2020 | 4 | 0 | 1 | 5 | Poor |
| Nguyen-Huynh 2020 | 4 | 2 | 1 | 7 | Poor |
| Nunez 2020 | 4 | 0 | 1 | 5 | Poor |
| Ogliari 2020 | 4 | 0 | 1 | 5 | Poor |
| Okwu 2020 | 4 | 0 | 1 | 5 | Poor |
| Orellana 2020 | 4 | 0 | 1 | 5 | Poor |
| Padmanabhan 2020 | 4 | 0 | 1 | 5 | Poor |
| Pagotto 2020 | 4 | 0 | 1 | 5 | Poor |
| Papafaklis 2020 | 4 | 0 | 1 | 5 | Poor |
| Patel 2020 | 4 | 1 | 1 | 6 | Poor |
| Patel 2020 | 4 | 1 | 3 | 8 | Good |
| Pathare 2020 | 4 | 0 | 1 | 5 | Poor |
| Patt 2020 | 4 | 0 | 1 | 5 | Poor |
| Piccininni 2020 | 4 | 0 | 1 | 5 | Poor |
| Pintado 2020 | 4 | 1 | 1 | 6 | Poor |
| Popovic 2020 | 4 | 0 | 1 | 5 | Poor |
| Pop 2020 | 4 | 0 | 0 | 4 | Poor |
| Quaquarini 2020 | 4 | 0 | 1 | 5 | Poor |
| RashidHons 2020 | 4 | 2 | 3 | 9 | Good |
| Rebecchi 2020 | 4 | 0 | 1 | 5 | Poor |
| Richter 2020 | 4 | 0 | 1 | 5 | Poor |
| Riemann 2020 | 4 | 0 | 1 | 5 | Poor |
| Rodriguez-Leor 2020 | 4 | 1 | 3 | 8 | Good |
| Rupa 2020 | 4 | 0 | 1 | 5 | Poor |
| Russo 2020 | 4 | 0 | 1 | 5 | Poor |
| Salarifar 2020 | 4 | 0 | 3 | 7 | Poor |
| Scholz 2020 | 4 | 1 | 1 | 6 | Poor |
| Scortichini 2020 | 4 | 1 | 1 | 6 | Poor |
| Secco 2020 | 4 | 1 | 1 | 6 | Poor |
| Seiffert 2020 | 4 | 1 | 1 | 6 | Poor |
| Sharma 2020 | 4 | 0 | 1 | 5 | Poor |
| Silva 2020 | 4 | 0 | 1 | 5 | Poor |
| Sinnathamby 2020 | 4 | 1 | 1 | 6 | Poor |
| Slullitel 2020 | 4 | 0 | 3 | 7 | Poor |
| Sobti 2020 | 4 | 0 | 3 | 7 | Poor |
| Stang 2020 | 4 | 0 | 1 | 5 | Poor |
| Stohr 2020 | 4 | 1 | 1 | 6 | Poor |
| Stokes 2020 | 4 | 0 | 1 | 5 | Poor |
| Strang 2020 | 4 | 1 | 1 | 6 | Poor |
| Strang 2020 | 4 | 0 | 1 | 5 | Poor |
| Strauss 2020 | 4 | 0 | 1 | 5 | Poor |
| Tanacan 2020 | 4 | 0 | 1 | 5 | Poor |
| Teo 2020 | 4 | 0 | 1 | 5 | Poor |
| Thakrar 2020 | 4 | 1 | 3 | 8 | Good |
| Tomasoni 2020 | 4 | 0 | 1 | 5 | Poor |
| Toner 2020 | 4 | 0 | 1 | 5 | Poor |
| Tousek 2020 | 4 | 0 | 1 | 5 | Poor |
| Trabattoni 2020 | 4 | 0 | 0 | 4 | Poor |
| Uchino 2020 | 4 | 0 | 1 | 5 | Poor |
| Vandoros 2020 | 4 | 1 | 1 | 6 | Poor |
| Vanni 2020 | 4 | 0 | 1 | 5 | Poor |
| Vestergaard 2020 | 4 | 0 | 1 | 5 | Poor |
| Vieira 2020 | 4 | 1 | 1 | 6 | Poor |
| Wang 2020 | 4 | 0 | 1 | 5 | Poor |
| Weinberger 2020 | 4 | 1 | 1 | 6 | Poor |
| Westgard 2020 | 4 | 0 | 1 | 5 | Poor |
| Wong 2020 | 4 | 1 | 3 | 8 | Good |
| Woolf 2020 | 4 | 1 | 1 | 6 | Poor |
| Yalamanchi 2020 | 4 | 0 | 1 | 5 | Poor |
| Zhang 2020 | 4 | 0 | 1 | 5 | Poor |

Non-randomized observational trials were assessed for ROB using the Newcastle-Ottawa Scale (NOS), examining the following domains: selection (max score of 4), comparability (max score of 2) and exposure (max score of 3) for cohort studies (Wells 2019). Quality of the studies were based on either good (3-4 stars in selection domain and 1-2 stars in comparability domain and 2-3 stars in outcome/exposure domain), fair (2 stars in selection domain and 1-2 stars in comparability domain and 2-3 stars in outcome/exposure domain) or poor (0-1 star in selection domain or 0 stars in comparability domain or 0-1 stars in outcome/exposure domain) quality (Wells 2019).

*Selection*

1. Representativeness of intervention cohort:
   1. Truly representative of average, treated probiotic patient in hospital*
   2. Somewhat representative of average, treated probiotic patient in hospital*
   3. Only selected group of patients
   4. No description of derivation cohort
2. Selection of non-intervention cohort:
   1. Drawn from same community as intervention/exposed cohort*
   2. Drawn from different source
   3. No description of the derivation of the non-exposed cohort
3. Ascertainment of intervention:
   1. Health record*
   2. Structured interview*
   3. Written self-report
   4. No description
4. Demonstration that outcome of interest was not present the start of the study:
   1. Yes*
   2. No

*Comparability*

1. Comparability of cohorts on the basis of the design or analysis:
   1. Study controls for baseline demographics (e.g. age)*
   2. Study controls for an additional factors (e.g. illness severity)*

*Outcome*

1. Assessment of outcome:
   1. Independent blind assessment*
   2. Record linkage*
   3. Self report
   4. No description
2. Was follow-up long enough for outcomes:
   1. Yes (median duration of follow-up 4 weeks)*
   2. No
3. Adequacy of follow-up cohort:
   1. Complete follow-up*
   2. Minimal loss to follow-up (<20%)*
   3. Follow-up rate <80% and no description of losses to follow-up
   4. No statement

**S5B Table: Risk of Bias Assessment for Observational Case-Control Studies – Newcastle-Ottawa Score**

| Studies: | Selection  (max 4 stars) | Comparability  (max 2 stars) | Outcome  (max 3 stars) | Total  (max 9 stars) | Study Quality  Grade |
| --- | --- | --- | --- | --- | --- |
| Bajunaid 2020 | 4 | 2 | 3 | 9 | Good |
| Patel 2020 | 4 | 1 | 3 | 8 | Good |
| Perkins 2020 | 4 | 0 | 2 | 6 | Poor |

Non-randomized observational trials were assessed for ROB using the Newcastle-Ottawa Scale (NOS), examining the following domains: selection (max score of 4), comparability (max score of 2) and exposure (max score of 3) for cohort studies (Wells 2019). Quality of the studies were based on either good (3-4 stars in selection domain and 1-2 stars in comparability domain and 2-3 stars in outcome/exposure domain), fair (2 stars in selection domain and 1-2 stars in comparability domain and 2-3 stars in outcome/exposure domain) or poor (0-1 star in selection domain or 0 stars in comparability domain or 0-1 stars in outcome/exposure domain) quality (Wells 2019).

*Selection*

1. Is case definition adequate?
   1. Yes, with independent validation*
   2. Yes, e.g. record linkage or based on self reports*
   3. No description
2. Representativeness of cases:
   1. Consecutive or obvious representativeness of cases*
   2. Potential for selection biases or not stated
3. Selection of controls
   1. Community controls*
   2. Hospital controls
   3. No description
4. Definition of controls:
   1. No history of disease (endpoint)*
   2. No description of source

*Comparability*

1. Comparability of cases and controls on the basis of the design or analysis:
   1. Study controls for age * (select the most important factor)
   2. Study controls for any additional factors* (This criteria could be modified to indicate specific control for a second important factor)

*Outcome*

1. Ascertainment of outcome:
   1. Secure record (e.g. surgical records)*
   2. Structured interview where blind to case/control status*
   3. Interview where not blind to case/control status
   4. Written self-report or medical record only
   5. No description
2. Same method for ascertainment for cases and controls:
   1. Yes*
   2. No
3. Non-response rate:
   1. Same rate for both groups*
   2. Non-respondents described
   3. Rate different and no designation
