# Supplementary material for: Non-COVID outcomes associated with the coronavirus disease-2019 (COVID-19) pandemic effects study (COPES): A systematic review and meta-analysis
Source: PLoS One. 2022 Jun 24;17(6):e0269871. doi: 10.1371/journal.pone.0269871 (PMC9231780; doi:10.1371/journal.pone.0269871)
Supplement: S6 Table — (DOCX) [file pone.0269871.s007.docx]

**S6 Table: Mortality, morbidity, hospitalizations/occupancy, disruption In care outcomes (with statistical significance)**

| **Study author (year)** | **Mortality** | **Significant change (mortality)**  **(95% CI or p-value <0.05)** | **Morbidity** | **Significant change (morbidity)**  **(95% CI or p-value <0.05)** | **Hospitalization/**  **occupancy** | **Significant Change (hospitalization/occupancy)**  **(95% CI or p-value <0.05)** | **Disruptions to care** | **Significant Change (disruptions to care)**  **(95% CI or p-value <0.05)** |
| --- | --- | --- | --- | --- | --- | --- | --- | --- |
| Abdelaziz 2020 | NR | NR | NR | NR | NR | N | Symptom-to-first medical contact: median 227 minutes (65-790) vs. 119 minutes (27-203), p = 0.01, Door-to-balloon time: median 47 minutes (38-63) vs. 48 minutes (39-70), p = 0.41, Troponin-I level on admission: median 2739 ng/L (932-10480) vs. 1245 (327-2789), p = 0.02 | Y |
| Agarwal  2020 | NR | NR | NR | NR | Routine inpatient services reduced 95.18% (722 vs. 6779), emergency inpatient services reduced 61.7% (452 vs. 1179), p < 0.05 | Y | Routine outpatient flow reduced by 97.14% (2936 vs. 102697), emergency services decreased by 35.25% (452 vs. 1179), reduced corneal donors by 99.61% (2 vs. 515), routine patient consultation reduced (71 ± 19/day vs. 978 ± 109/day, p < 0.001), major surgeries reduced 98.19% (162 vs. 8932), teleconsultation increased (2616 vs. 0) | Y |
| Agarwal 2020 | 7.7% vs. 2.5%, p = 0.004 | Y | Good disposition: 80.6% vs. 90.7%, p = 0.011, good discharge mRS (0-3): 57.3% vs. 68.3%, p = 0.021 | Y | NR | N | Door-to-needle: 36 mins (26-53) vs. 35 (27-47.5), p = 0.83, Door-to-reperfusion: 103 mins (92-160) vs. 97 (82-123), p = 0.06, Last-known well-arrival: 283 mins (89-769) vs. 326 (93-936), p = 0.42 | N |
| Aldujeli 2020 | STEMI: 4.3% vs. 6.7%, p = 0.70, NSTEMI: 6.7% vs. 4.8%, p = 0.66 | N | In-hospital CPR: STEMI: 4.3% vs. 6.7%, p = 0.69, NSTEMI: 6.7% vs. 4.8%, p = 0.66, In-hospital ischemic stroke: STEMI: 2.1% vs. 0%, p = 0.69, NSTEMI: 0% vs. 0%, In-hospital hemorrhagic stroke: STEMI: 0% vs. 0%, NSTEMI: 0% vs. 0% | N | NR | N | Pain-to-door time: STEMI: 620 mins (225-1500) vs. 349 (146-659), p = 0.01, NSTEMI: 1855 mins (880-5732) vs. 606 (388-944), p <0.0001, Door-to-reperfusion time: STEMI: 76 mins (64-113) vs. 86 mins (56-126), p = 0.98, NSTEMI: 332 mins (182-581) vs. 194 (92-329), p = 0.04 | Y |
| Amaddeo 2020 | NR | NR | NR | NR | NR | N | Treatment delay >1 month: 21.5% vs. 9.5%, p < 0.001, Changed/delayed treatment: OR 9.66 (95% CI: 2.85-32.72), p <0.001 | Y |
| Amoo 2020 | 1 death (1%) vs. 1 (1%) death, p = NS | N | 30-day morbidity: 6 (6.3%) [pulmonary embolism, hydrocephalus, dysphasia, hematoma, facial weakness, surgical site infection/sepsis] vs. 7 (8.75%) [pneumonia, cerebral edema, epistaxis, pseudomeningocele, subdural hematoma, intracranial hemorrhage, diabetes insipidus], p = NS | N | NR | N | Inter-hospital transfer length of time: mean: 76 h vs 93 h in 2019, p = 0.10; Admission to surgery: 2.39 vs 2.89 days | N |
| Amukotuwa 2020 | NR | NR | NR | NR | NR | N | Endovascular clot retrievals (ECR): decreased 16 in March 2020 and 11 in April 2020 vs. 18.4, p = 0.165, Direct presentation for ECR: 36.36% vs. 68.75% (April to March 2020), p < 0.025, Door-to-CT time: 9 mins (7-16) vs. 22 (15-32) | Y |
| Andersson 2020 | Mortality: 142 versus 132 per 1000 person-years; age- and sex-adjusted mortality rate ratio, 1.05 [95% CI, 0.93–1.18]; P=0.45. 90 patients with heart failure diagnosed with COVID-19 (0.08%). 25 of 90 died from COVID-19 (mortality: 37% [95% CI: 23-50%] within 15 days of COVID-19 diagnosis) | N | NR | NR | Hospitalizations for worsening heart failure: 1.04 vs. 0.93 per 1000 person-years; P=0.02. During lockdown: rates of new-onset heart failure diagnoses (1.26 versus 2.25 per 1000 person-years, p<0.0001); Hospitalizations for worsening heart failure (0.63 versus 0.99 per 1000 person-years, p <0.0001). | Y | NR | NR |
| Anteby 2020 | 2020 vs. 2019 vs. 2018: 0.4% vs. 0.96% vs. 0.4% (p = 0.54) | N | NR | NR | Admitted: 2020 vs. 2019 vs. 2018: 316 (41%) vs. 376 (33%) vs. 396 (37%), p = 0.01 | Y | NR | NR |
| Arafa 2020 | 2020 COVID+: 36.8% vs. 2020 COVID-: 11.5% vs. 2019: 11.7%, p = 0.014 | Y | Intra-op complications: 2020 COVID+: 5.9% vs. 2020 COVID-: 3.9% vs. 2019: 3.5%, Post-op delirium: 2020 COVID+: 17.6% vs. 2020 COVID-: 15.6% vs. 2019: 22.8%, p =0.056, Wound infection: 0 vs. 0 vs. 1.8%, p = 0.492, Falls: 17.6% vs. 5.2% vs. 0.0%, p = 0.012, Re-operation: 0.0% vs. 2.6% vs. 1.8%, p = 1.0 | Y | Length of stay: COVID+ 2020: 24.21 days (19.29), p = 0.068 | N | NR | NR |
| Athiel 2020 | NR | NR | NR | NR | Hospitalization rate: Mar-May 2020 (5.3%) vs. Dec 2019-Feb 2020 (4.6%) vs. Mar-May 2019 (3.9%) vs. Dec 2018-Feb 2019 (4.4%) | NR | Emergency hospitalizations: Mar-May 2020 (1081) vs. Dec 2019-Feb 2020 (976) vs. Mar-May 2019 (1078) vs. Dec 2018-Feb 2019 (785), Consultations in gynecological emergency units: Mar-May 2020 (24095) vs. Dec 2019-Feb 2020 (24982) vs. Mar-May 2019 (24217) vs. Dec 2018-Feb 2019 (14708), p ˂ 0.000000001 | Y |
| Aviran 2020 | 30-day mortality: 4.2% vs. 3.7%, p = 0.76 | N | Complications: 22.7% vs. 18.3%, p = 0.347, Severe complications: 4.2 vs. 3.7% | N | Admissions: 249 vs. 347 (25% less, p <0.0001), | Y | Emergency department length of stay: 7.33 hours (3.48) vs. 6.46 (3.16), p = 0.003, Hospital length of stay: 3 days (1-42) vs. 4 (1-77), p = 0.048 | Y |
| Baert 2020 | 30-day mortality: 100% vs. 96.5%, p = 0.001 | Y | NR | NR | NR | N | Bystanders initiated CPR: 49.8% vs. 54.9%; difference, − 5.1 percentage points [95% CI, − 9.1 to − 1.2]; First aid responder initiated CPR: 84.3% vs. 88.7%; difference, − 4.4% points [95% CI, − 7.1 to − 1.6], mobile medical teams CPR: 67.3% vs. 75.0%; difference, − 7.7% points [95% CI, − 11.3 to − 4.1]). First aid providers used defibrillators: (66.0% vs. 74.1%; − 8.2% points [95% CI, − 11.8 to − 4.6]). Return of spontaneous circulation (ROSC): 19.5% vs. 25.3%; difference, − 5.8 % points [95% CI, − 9.0 to − 2.5] | Y |
| Bajunaid 2020 | 30-day mortality: 1.6% vs. 1.8%, p = 0.84 | N | General complications: 3.61% vs. 5.7%, p = NS, Craniosacral complications: 9.18% vs. 9.17%, p = NS | N | Length of stay: 6 days (3-14.5) vs. 7 days (4-14), p = NS | N | NR | NR |
| Ball 2020 | Discharged alive: 20.0% vs. 36.7%, p =0.004, Survival to discharge: 6.1% vs. 11.7%, p = 0.002 | Y | NR | NR | NR | N | Initiation of resuscitation by EMS: 40.6% vs. 46.9%, p = 0.001, Median response time: 8 mins (6.5-10.6) vs. 7.5 (6-10.2), p = 0.001, median time-to-first shock: 14 mins (10-19.5) vs. 11 (9-17), p < 0.001 | Y |
| Barten 2020 | NR | NR | NR | NR | Emergency department volume: -18% in 2020 vs. 2019, -29% further down once lockdown initiated (no p-values reported) | NR | NR | NR |
| Batra 2020 | Absolute value of mortality in COVID tracheostomy patients vs Non-COVID time matched controls | NR | Clavien-Dindo grades 3 and 4 (COVID group = 3/21 vs Non-COVID group = 41/193; p = 0.57) Group A = Covid Group; Group B = Non-Covid time matched control The level of major morbidity was similar in the two groups, i.e. 14.3% in group A and 21.2% in group B, although the median hospital stay was significantly longer in group A (10 days) than in group B (7 days) (P = 0.001). | Y | N/A | N | Changes in practice were further classified into three categories: (1) anaesthesia-related changes; (2) surgery-related changes; and (3) OR-related change. Increased rescheduling of surgerys, longer hospital stays BUT definitive cancer care surgery has NOT been deferred and max patient and healthcare worker saftey has been insured. Group A = Covid Group; Group B = Non-Covid time matched control In group A, six of 21 patients (28.6%) underwent an elective preoperative tracheostomy compared to 13 of 193 in group B (6.7%) (P = 0.005). Median hospital stay was significantly longer in group A, i.e. 10 days (range 1–19 days) compared to 7 days (range 2–37 days) in group B (P = 0.001). Twelve of the 21 patients (57.1%) in group A had their surgery postponed in comparison to 53 of 193 control patients in group B (27.5%) (P = 0.01). | Y |
| Becq 2020 | NA | NR | NA | NR | NA | N | Urgent endoscopies outside regular working hours in the Paris area decreased as a result describe the observed number of acts during the 2020 mandatory period of home isolation, compared to the values in prior years and the expected value for 2020.  From January 17th to March 16th, the number of acts was respectively 252 and 265 in 2018 and 2019.   The observed number of acts within the same period in 2020 (two-month period before home isolation) was 252, similar to the estimated number of 258 (-2.3%). No p-values.  From March 17th to April 17th, the number of acts was respectively 147 and 137 in 2018 and 2019.   The observed number of urgent endoscopies within the same period in 2020 (one-month of home isolation) was 79, lower than the estimated number of 142 (-44.0%) and compared to the 137 acts of 2019 (-44.4%). No p-values.  Beginning of Pandemic urgent endoscopies to home isolation period decreased 52.1% (January 17th to April 17th) N = 117 vs 56. No p-values. | Y |
| Benites-Goni 2020 | NA | NR | NA | NR | NA | N | Time between arrival at the hospital and the performance of the endoscopy was significantly longer during the pandemic (10.00 vs. 13.08 hours, p-value = 0.019).  No significant differences in hospital stay or mortality | Y |
| Bhatt 2020 | In-hospital mortality was not signiﬁcantly different (6.2% vs. 4.4%; p = 0.30) in March 2020 compared with March 2019. | N | NA | NR | 43.4% (95% conﬁdence interval [CI]: 27.4% to 56.0%) fewer estimated daily hospitalizations in March 2020 compared with March 2019 (p < 0.001).   The daily rate of hospitalizations did not change throughout 2019 (–0.01% per day [95% CI: –0.04% to +0.02%]; p = 0.50), January 2020 (–0.5% per day [95% CI: –1.6% to +0.5%]; p = 0.31), or February 2020 (+0.7% per day [95% CI: –0.6% to +2.0%]; p = 0.27).   There was signiﬁcant daily decline in hospitalizations in March 2020 (–5.9% per day [95% CI: –7.6% to –4.3%]; p < 0.001). Length of stay was shorter (4.8 days [25th to 75th percentiles: 2.4 to 8.3 days] vs. 6.0 days [25th to 75th percentiles: 3.1 to 9.6 days]; p = 0.003)  Proportion of total cardiovascular hospitalizations that were for STEMI (ST elevation myocardial infarction) was comparable between March 2020 and before March 2020 (3.1% vs. 3.7%; p = 0.59). Similar results were seen when comparing March 2020 to March 2019 (3.1% vs. 5.1%; p = 0.21). | Y | Median length of stay was signiﬁcantly shorter in patients admitted in March 2020 compared with those admitted before March 2020 (4.8 [25th to 75th percentiles: 2.4 to 8.3] days vs. 5.7 [25th to 75th percentiles: 3.0 to 10.0] days; p = 0.001) and compared with month-matched patients admitted in March 2019 (4.8 [25th to 75th percentiles: 2.4 to 8.3] days vs. 6.0 [25th to 75th percentiles: 3.1 to 9.6] days; p = 0.003)  The estimated volume of daily acute cardiovascular hospitalizations was similar in January 2020 compared with month-matched hospitalizations in January 2019 (difference: – 6.5%; 95% confidence interval [CI]: – 17.9 to 6.6; p = 0.31) and in February 2020 compared with February 2019 (difference: – 9.3%; 95% CI: – 21.2% to 4.4%; p = 0.17). Conversely, there was a signifi cant decline of 43.4% (95% CI: 27.4% to 56.0%) in the total number cardiovascular hospitalizations in March 2020 compared with March 2019 (260 hospitalizations vs. 475 hospitalizations; p <0.001).  There were no signiﬁcant changes in the daily rates of cardiovascular hospitalizations throughout 2019 (–0.01% per day [95% CI: –0.04% to +0.02%]; p = 0.50), including during the ﬁrst 3 months of 2019 (–0.2% per day [95% CI: –0.4% to +0.1%]; p = 0.18).   Similarly, there were no significant changes in daily cardiovascular hospitalization rates in January 2020 (–0.5% per day [95% CI: –1.6% to=0.5%]; p = 0.31) and February 2020 (+0.7% per day [95% CI: –0.6% to +2.0%]; p = 0.27). In contrast, there was a signiﬁcant incremental daily decline in cardiovascular admissions across the health system in March 2020 (–5.9% per day [95% CI: –7.6% to –4.3%]; p < 0.001)  Declines corresponded with key COVID-19–related events, state guidance, and a rise in cumulative conﬁrmed cases of COVID-19 in Massachusetts. | Y |
| Bilinski 2020 | COVID MORTALITY US reported a total of 198 589 COVID-19 deaths (60.3/100 000), higher than countries with low and moderate COVID-19 mortality but comparable with high-mortality countries. For instance, Australia (low mortality) had 3.3 deaths per 100 000 and Canada (moderate mortality) had 24.6 per 100 000. Conversely, Italy had 59.1 COVID-19 deaths per 100 000; Belgium had 86.8 per 100 000. If the US death rates were comparable to Australia, the US would have had 187 661 fewer COVID-19 deaths (94% of reported deaths), and if comparable with Canada, 117 622 fewer deaths (59%), no p-value.  All-Cause Mortality 14 countries with all-cause mortality data, the patterns found for COVID-19–specific deaths were similar for excess all-cause mortality. In countries with moderate COVID-19 mortality, excess all-cause mortality remained negligible throughout the pandemic. In countries with high COVID-19 mortality, excess all-cause mortality reached as high as 102.1/100 000 in Spain, while in the US it was 71.6/ 100 000. However, since May 10 and June 7, excess all-cause mortality was higher in the US than in all high-mortality countries, no p-value | NR | NA | NR | NA | N | NA | N |
| Birkmeyer 2020 | Relative to the 2.1 percent in-hospital mortality rate for non-COVID-19 admissions during February 2020, in-hospital mortality rose by 0.3 percent (p< 0.05) in April before returning to baseline in May and June.  Stratified analysis of in-hospital mortality (with coefficients reported in the appendix and adjusting for age, sex, and diagnosis), suggested that much of the increase in in-hospital mortality for non-COVID admissions during April occurred for patients living in majority-Black, majority Hispanic, or high-poverty ZIP codes. In April, mortality in this subgroup was 0.5 percent higher than for those not in the subgroup (p = 0.03). By the end of May, that disparity in mortality had disappeared. | Y | NA | NR | Non-COVID-19 admission volumes declined substantially for all twenty primary medical conditions or diagnoses in April 2020.   Conditions that declined least included pancreatitis ( − 23.7 percent; p< 0.05), stroke ( − 24.7 percent; p< 0.05), and altered mental status ( − 27.1 percent; p< 0.05). Several conditions associated with much larger admission declines included chronic obstructive pulmonary disease (COPD)/asthma ( − 68.6 per- cent; p< 0.05), non-COVID-19 pneumonia ( − 53.6 percent, p< 0.05), and transient ischemic attack ( − 50.9 percent, p< 0.05).   Although not highlighted in the exhibits, patients with COVID-19 accounted for a large proportion of admissions with respiratory failure (73.7 percent), pneumonia (70.1 percent), and sepsis (38.2 percent); total admissions fell less for these three conditions than they did for most other medical illnesses (data not shown).   June/July, admissions for pancreatitis, alcohol-related conditions, and diabetes had returned to baseline levels. Non-COVID-19 admissions for urinary tract infection ( − 24.3 percent; p< 0.05), sepsis ( − 25.1 percent; p< 0.05), COPD/asthma ( − 40.1 percent; p< 0.05), and pneumonia ( − 44.1 percent; p< 0.05) remained substantially depressed.   Declines in non-COVID admissions for patients with acute ST-elevation myocardial infarction ( − 22.2 percent; p< 0.05) and stroke ( − 16.9 percent; p< 0.05). | Y | NA | NR |
| Blangiardo 2020 | Regional specific all-cause excess mortality rates in Italy. Various regions had singificant excess deaths, but overall Italy recorded barely any excess death country wide during the first four months of 2020. Macro- region  All cause deaths  Expected deaths posterior mean (95% interval)  Total excess deaths posterior mean (95% interval)  COVID-19 deaths  Non-COVID-19 excess deaths posterior mean (95% interval)  No p-values.  **colums of excess mortality are as above and in order for each region below North West 18,059 11,117 (10,392 to 11,917) 6,942 (6,142 to 7,667) 4,370 2,572 (1,772 to 3,297)  Lombardia 39,397 15,451 (14,611 to 16,384) 23,946 (23,013 to 24,786) 13,749 10,197 (9,264 to 11,037)  North East 26,694 18,661 (17,650 to 19,633) 8,033 (7,061 to 9,044) 5,986 2,047 (1,075 to 3,058)  Centre 20,903 19,315 (18,203 to 20,499) 1,588 (404 to 2,700) 2,256 -668 (-1,852 to 444)  South + Islands 31,367 30,846 (29,268 to 32,387) 521 (-1,020 to 2,098) 1,577 -1,056 (-2,597 to 521) | Y | NA | NR | NA | N | NA | N |
| Boyarsky 2020 | Waitlist death stratified by states with rates of COVID-19 and presented in Incidence Rate Ratio  2.22 (1.88, 2.52) waitlist death in states with very high COVID-19 rates, no p-value. Other states rates were not appreciably raised. | NR | IRR (Incident Rate Ratio) - Decreased Rates of DDKT (deceased donor kidney transplants) and LDKT (living donor kidney transplants)  DDKT - 0.76 0verall (0.73, 0.80)  LDKT - 0.14 overall (0.11, 0.15)*** >> markably decreased living donor kidney transplants in all states despite COVID-19 rates varying between states.  **Graphs in paper show variation in absolute counts of DDKT and LDKT during the early stages of the pandemic | Y | NA | N | NA | N |
| Bromage 2020 | In-hospital mortality was very low in both years, which precluded statistical comparison. | N | NA | NR | Patients had similar mean age at AHF (Acute heart faliure) admission (73 ± 1 4 years in 2020 vs. 71 ± 1 5 years in 20 1 9), similar proportions of women (46% vs. 42%) and similar ethnicity (42% vs. 39%). Overall, comorbidities and cardiovascular risk factors were similar between patients presenting in each year, although fewer patients in 2020 had pre-existing, significant valve disease (27% vs. 5 1 %, P = 0.04). Furthermore, similar proportions presented with decompensated HFrEF (Heart Faliure Reduced Ejection Fraction) (69% vs. 63%) and HF (Heart Faliure) with preserved ejection fraction (3 1 % vs. 37%) and this is consistent with overall NHFA (National Heart Foundation of Australia) data. We observed that patients hospitalized in 2020 had a higher proportion with NYHA (New York Heart Association) class III or IV symptoms (96% vs. 77%, P = 0.03) and severe peripheral oedema (39% vs. 1 4%, P = 0.01). In-hospital management of patients was similar in our centre in 2019 and 2020. Fewer patients were admitted to cardiology wards (23% vs. 37%) or reviewed by HF specialists (56% vs. 78%) | Y | NA | NR |
| Bugger 2020 | Death was reported in n (%) and compared by poisson regression for comparison of MI (myocardial infarction), MI+STEMI (ST elevation myocardial infarction), MI+NSTEMI (non ST elevation myocardial infarction), PE (pulmonary embolism), AAD (abdominal aortic dissection), MI+PE (pulmonary embolism) and MI+PE+AAD excess mortality compared between 2016-2020 periods in correspoinding weeks. Only MI, MI + PE and MI+PE+AAD were p<0.05.  Cumulative admissions compared between 2016-2020 (MI, PE, AAD) and in-hospital mortality. RR = 1.65 (p=0.041) | Y | NA | NR | Decreased hospital admissions for cardiovascular emergencies during COVID-19 associated  RR (Relative Risk) 2016-2019 compared to 2020  MI (Myocardial Infarction) RR = 0.77; p=0.004  PE (Pulmonary Embolism) RR = 0.79; p=0.056 AAD (Abdominal Aortic Dissection) = NS (not significant) MI+PE+AAD = 0.77; p<0.001 | Y | NA | NR |
| BustosSierra 2020 | Nine thousand five hundred ninety-one COVID-19 deaths and 39,076 deaths from all-causes were recorded, with a correlation of 94% (Spearman’s rho, p < 0.01).   During the period with statistically significant excess mortality (March 20th to April 28th; total excess mortality 64.7%), 7917 excess deaths were observed among the 20,159 deaths from all-causes. In the same period, 7576 COVID-19 deaths were notified, indicating that 96% of the excess mortality were likely attributable to COVID-19. Excess deaths were based on the Be-MOMO method explained in the paper from historical values in the last centurly (back to 1900). No p-value.   "Number of deaths in April 2020 was also similar to that of the flu epidemics of January 1951 and February 1960, which claimed about 15,500 deaths per month." | Y | NA | NR | NA | N | NA | N |
| Butt 2020 | NA | NR | 16–37% decline in ED volumes overall, with a 25–50% decline in patients presenting with cardiac symptoms in March and April 2020 compared with March and April 2019. Among those presenting with cardiac symptoms, we observed a 24–43% decline in cardiac diagnoses in March and April 2020 compared with March and April 2019. | N | NA | N | NA | N |
| Butt 2020 | Unadjusted mortality rate (number of deaths recorded in a given month divided by the population in the same month) per 100,000 population was 7.91 for March 2020, 6.92 for January 2020, and 6.48 for March 2019. Total number of deaths recorded were 221 for March 2020, 192 for January 2010, and 179 for March 2019. No p-value. | NR | NA | NR | Between January 2020 and March 2020, there was a decrease in admissions for seven of the eight conditions. Admissions were lower for acute appendicitis (-17.4%), ACS (acute coronary syndrome) (-57.9%), other cardiovascular disease diagnoses (-48.4%), stroke (-30%), bone fractures (-8.7%), cancer (-12.9%), and live births (-8.8%), whereas admissions were higher for respiratory tract infections (+15.1%).   Respiratory tract infection admissions included 88 patients with a diagnosis of ‘coronavirus infection’ who were diagnosed upon routine testing after admission.   Between March 2019 and March 2020, there was also a decrease in admissions for the same seven of the eight conditions.   Admissions were lower for acute appendicitis (-22.8%), ACS (-50.0%), other cardiovascular disease diagnoses (-81.3%), stroke (-31.3%), bone fractures (-38.8%), cancer (-23.9%), and live births (-17.0%), whereas admissions were higher for respiratory tract infections (+56.1%). No p-values. | Y | NA | NR |
| Calderon-Larranaga 2020 | EM (excess mortality) was first detected during the week of 23–29 March 2020. During the peak week of the epidemic (6–12 April 2020), an EM of 150% was observed (152% in 80+ year old women; 183% in 80+ year old men). During the same week, the highest EM was observed for DeSOs (Demographic Statistics) with lowest income (171%), lowest education (162%), lowest share of Swedish- born (178%) and lowest share of gainfully employed residents (174%). EM was further increased in areas with higher versus lower proportion of younger people (magnitude of increase: 1.2–1.7 times depending on socioeconomic measure). No p-values. | NR | NA | NR | NA | N | NA | N |
| Cannata 2020 | In-hospital mortality was significantly higher in 2020 compared with 2019 (p = 0.015)  Kaplan–Meier curves for in-hospital mortality in 2020 vs. 2019. Significantly higher during COVID19 p=0.001 | Y | NA | NR | NA | N | Median length of stay was comparable between 2019 and 2020 (6 days, IQR 3–12 days and 7 days, IQR 3–14 days, respectively; P = 0.29), although patients admitted in 2020 showed a lower readmission rate (5% in 2020 vs. 8% in 2019; P = 0.03). | Y |
| Cannavo 2020 | NA | NR | NA | NR | NA | N | During period A, 26 donors (45%) were excluded, mainly because of objection for organ donation (17/26, 65.4%); of these, 6 were excluded for unacceptable risk (23%), of whom half (3/6) were excluded for the presence of an active cancer and half because of COVID-19 positive BAL (brochealveolar lavage) (3/6).  We did not observe any difference in terms of consent rate or any other reason for excluding donors across the 3 periods (P value = .956).  During period A, a 39% reduction of total deceased donation rate was observed as compared to period B and a 55% reduction as compared to period C. Considering used donors, the reduction was 47% and 60%, respectively.No p-values.  Cross-sectional study included all consecutive potential deceased donors proposed by any NITp (Northern Italy Transplant program) ICUs during the 6 weeks after February 21, 2020 (period A) compared to all potential donors during the same time frame of 2019 (6 weeks after February 21, 2019; period B) and during the 6 weeks before February 20, 2020 (period C). The 3 periods were compared to investigate any change in the total number of potential and eligible donations, donors’ procurement, and total transplants by solid organ and priority reason for assignment. Living donors and donors outside the NITp (Northern Italy Transplant program) regions were excluded. | N |
| Cano-Valderrama 2020 | Mortality (6.7% vs. 4.3%, p = 0.358) were similar in both groups | N | Morbidity was higher during pandemic period (34.7% vs. 47.1%, p = 0.022), although this diﬀerence was not statistically signiﬁcant in the multivariate analysis. (OR = 1.2, 95% CI 0.7–2.2, p = 0.501).  A signiﬁcant increase in the rate of complicated appendicitis was observed during the pandemic period (7.95% vs. 42.50%, p < 0.001). | Y | Mean number of patients who underwent Acute Care Surgery during the control and pandemic periods was 2.3 and 0.9 patients per day and hospital (p < 0.001), representing a 58.9% decrease in Acute Care Surgery activity. | Y | Time from symptoms onset to patient arrival at the Emergency Department was longer during the pandemic (44.6 vs. 71.0 h, p < 0.001). Surgeries due to acute cholecystitis and complications from previous elective procedures decreased (26.7% vs. 9.4%) during the pandemic, while bowel obstructions and abdominal wall hernia surgeries increased (12.3% vs. 22.2%) (p = 0.001).  Mean number of patients who underwent ACS (Acute Care Surgery) during the control and pandemic periods was 2.3 and 0.9 patients per day per hospital respectively (diﬀerence 1.3 patients per day, CI 1.0–1.7, p < 0.001)  Even though the percentage of patients who underwent ACS due to acute appendicitis or anorectal abscess were similar in both periods, acute cholecystitis and surgeries required for treating complications of previous elective procedures decreased (26.67% vs. 9.40%; no p-value) and at the same time, surgical interventions due to intestinal obstruction and reparation of abdominal wall hernia increased (12.28% vs. 22.22%), no p-value. Interestingly, in the group of patients diagnosed with acute appendicitis, a signiﬁcant increase in the rate of complicated appendicitis was observed during the pandemic period (7.95% vs. 42.50%, p < 0.001). | Y |
| Casalino 2020 | NA | NR | NA | NR | During the influenza epidemic period, we found increasing trends in ED visits, medical/surgical ward (MSW), and ICU admissions (10.6%, 12.8%, and 4.6%, respectively), while for the COVID-19 epidemic period, we observed a decreasing trend in the number of ED visits (36.9%) and also a pattern of growth for MSW (medical/surgical wards) and ICU admissions (33.9% and 277.0%, respectively). During lockdown, ED visits and admissions to MSW and ICU declined (49.6%, 16.8%, and 15.5%, respectively). No p-values. | N | NA | NR |
| Cates 2020 | 21% vs 3.8% in-hospital mortality for COVID time period compared to influenza p<0.001 | Y | Largery increased morbidities in all categories.  Compared with patients with influenza, patients with COVID-19 had two times the risk for pneumonia, 1.7 times the risk for respiratory failure, 19 times the risk for ARDS (Acute Respiratory Distres Syndrome), and 3.5 times the risk for pneumothorax, underscoring the severity of COVID-19 respiratory illness relative to that of influenza  Risk for asthma and COPD exacerbations was approximately three times lower among patients with COVID-19 than among those with influenza.  Black patients accounted for 48.3% of COVID-19 patients and 24.7% of influenza patients; the proportion of Hispanic patients was similar in both groups. The percentage of COVID-19 patients admitted to an ICU (36.5%) was more than twice that of influenza patients (17.6%); the percentage of COVID-19 patients who died while hospitalized (21.0%) was more than five times that of influenza patients (3.8%); and the duration of hospitalization was almost three times longer for COVID-19 patients (median 8.6 days; IQR = 3.9–18.6 days) than that for influenza patients (3.0 days; 1.8–6.5 days) (p<0.001 for all). | N | 36.5% (n=828) vs 17.6% (n=961); p<0.001 | Y | NA | NR |
| Cevallos-Valdiviezo 2020 | From March 17th 2020 to Oct 22 2020, a total of 80,108 deaths of all causes were registered in Ecuador, of which 36,922 (95% CI: 32314-42696) were estimate to be in excess of expected levels. No p-values. | Y | NA | NR | NA | N | NA | N |
| Chan 2020 | NR | NR | Decreased hospitalizations for Acute Exacerbation of Chronic Obstructive Pulmonary Disease (AECOPD) AECOPD over a 3 month period  AECOPD severity indicators of length of stay (LOS) >5 days (%) and Mortality were reported to indicate acute exacerbation of chronic obstructive pulmonary disease (AECOPD) severity | N | 44% decrease in acute exacerbation of chronic obstructive pulmonary disease (AECOPD) admissions vs other diseases during COVID-19. No p-values reported. | N | Acute Exacerbation of Chronic Obstructive Pulmonary Disease (AECOPD) admissions monthly average decreased from 92 hospitalizations (2015-2019, Jan-Mar) to 41 (Jan-Mar 2020) - P value < 0.005. Significant decrease of 44% (95% CI 36.4%-52.8%, p < 0.001).  AECOPD hospitilizations were significantly lower in the 1st 3 months of 2020 compared to the same periods in previous years. Universal masking and higher temperature were correlated with decrease in AECOPD. Admissions dropped 44%. No p-value. | Y |
| Chan 2020 | Survival to discharge 17% lower during COVID-19  (adjusted RR, 0.83 [95% CI, 0.69-1.00]; P = 0.048) | Y | Rates of return of spontaneous circulation were 18% lower overall than before the pandemic, including 11% to 15% lower in communities with low COVID-19 mortality. Rates of survival to discharge were 17% lower, primarily in communities with moderate to high COVID-19 mortality, and incidence of Out-of-hospital cardiac arrest (OHCA) was higher, but largely in communities with high COVID-19 mortality.  P < 0.001 | Y | NA | N | Emergency Medical Services (EMS) treatment time intervals, median (IQR) *Time from 911 call to EMS arrival:* 2019: 8.5 (6.5-11.3)  2020: 9.0 (6.9-12.0)  Standardized difference, %: 0.5 *Duration of EMS Treatment* 2019: 22.2 (15.0-32.0) 2020: 24.8 (16.7-37.0) Standardized difference, %: 3.4  No p-values.  Rates of return of spontaneous circulation were 18% lower overall than before the pandemic, including 11% to 15% lower in communities with low COVID-19 mortality. Rates of survival to discharge were 17% lower, primarily in communities with moderate to high COVID-19 mortality, and incidence of Out-of-hospital cardiac arrest was higher, but largely in communities with high COVID-19 mortality. No p-values. | Y |
| Claeys 2020 | Comparable; 5.9% vs 6.7% hospital mortality   5 of 7 (71%) of COVID-19 Positive ST Elevation Myocardial Infarct (STEMI) patients died versus 3.3% (6/181) for the patients without evidence of COVID-19 infection (p <.0001). | Y | 188 ST Elevation Myocardial Infarct (STEMI) patients admitted for percutaneous coronary intervention during 3 month lockdown period vs average of 254 STEMI patients pre-lockdown periods (IRR of 0.74, p = 0.001)  Reperfusion strategy was predominatly PCI in both time periods (95 vs 96%).   Measured clinical characteristics, reperfusion therapy modalities, COVID-19 status | Y | Admission for ST Elevation Myocardial Infarction (STEMI) and percutaneous coronary intervention treatment: 188 STEMI patients admitted for PCI during 3 month lockdown period vs average of 254 STEMI patients pre-lockdown periods (incidence rate ratio: IRR of 0.74, p = 0.001) | Y | Significant delay in treatment initiation during lockdown period with more late presentations [14% vs 7.6, p =0.04] and longer "door to balloon times" [median 45 vs 39 min, p = 0.02]  Late presentation (>12h after onset) was observed in 14% of patients during lockdown vs 7.6% before lockdown (p=0.04)  Time delay between diagnosis and treatment was comparable. | Y |
| D'Apolito 2020 | The mortality in the 7-week period of the current year was 1.2% compared with 0% in 2019 [2 admitted patients died from COVID-19]. No p-values | NR | Main focus of study was impact on total joint arthroplasty | N | The mean length of stay in the arthroplasty department was 5 days in 2019 and 5.8 days this year (range, 4 to 6)  Patients admitted to the rehabilitation department after total joint arthroplasty (TJA) were 323 in 2019 (46% of all TJAs) and 45 in 2020 (27%) | Y | Number of hip and knee arthroplasties decreased from 706 in 2019 to 166 (76.5%) in same period of 2020. In 2019, a mean of 101 ± 9 hip and knee arthroplasties were performed per week compared with a mean of 24 ± 34 in 2020. No p-values. | NR |
| Davies 2020 | 30-day mortality was 0% (no p-values) | NR | Rate of noscomia COVID-19l infection (2020): 0% | N | NA | N | Fewer elective operations were performed in 2020 (258) compared with 2019 (1196) and 2018 (1261). No p-value. | N |
| Dawoud 2020 | NR | NR | 33% reduction (n=75 (2019) vs n=50 (2020)) in number of patients admitted during COVID-19 lockdown for cervicofacial infection of odontogenic origin | N | 33% reduction in number of patients admitted. No p-values reported. | NR | Proportionally more patients required extra-oral approach (p = 0.056) to drainage within the COVID-19 lockdown cohort than pre COVID-19. Whilst not statistically significant, this suggests that the severity of disease on admission is worse in the COVID-19 cohort. | N |
| Dayananda 2020 | COVID-19 mortality rate was 1.8% (n = 7). All had relevant comorbidities and median age of 92 years.  30 day mortality (including COVID-19 positive cases) was 4% (n=16), an increase from 1.2% (n=7) in 2019. (χ2(2) = 7.45; p = 0.006, chi-squared test). Excluding patients with COVID-19, mortality rates were 2.3% (n = 9) in the 2020 cohort, a statistically non-significant increase from 2019 (χ2(2) = 1.2; p = 0.273, chi-squared test) | Y | Of the 18 (4.5%) patients testing positive for COVID-19, 4 tested positive at the "clean" non-COVID-19 site   Comorbidities were recorded, as were COVID-19 positives at the segregated sites (the COVID-19 site UHW and "clean site")  COVID-19 mortality was recorded as a breakdown of cormorbidities | N | 34% comparative reduction in trauma surgery in 2020. No p-values reported. Median length of stay, days was reported for each injury to anatomical region. No p-values reported. | NR | A significant reduction in time to surgery was seen in 2020 using the split-service model.  In 2020, 85% of Femoral Fragility Fractures (FFF) patients received surgery within 24 hours of admission, compared to 60% in 2019. There was a statistically significant increase in consultant led surgery and an increased use of spinal, regional and local anaesthesia in 2020. Inpatient length of stay (LOS) significantly reduced in 2020 for injuries within most anatomical regions. Analysis of the Femoral Fragility Fractures (FFF) patients demonstrated a significant reduction in LOS, allowing for some patients who remain as inpatients at the time of analysis. LOS did not reduce for spinal or polytrauma patients in 2020. No p-values. | NR |
| deHavenon 2020 | Risk of death: matched cohort of patients with ischemic stroke (IS)-COVID and IS-PNA (Pneumonia), IS-COVID had a higher risk of death (IPW-weighted OR 1.56, 95% CI 1.33-1.82) and lower odds of favorable discharge (IPW-weighted (inverse probablity) OR 0.63, 95% CI 0.54-0.73) than both the IS control group and the IS pneumonia group (PNA)  IS-COVID patients were more than four times as likely to die in-hospital compared to IS controls (30.4% vs. 6.5%, p<0.001) | Y | Compared to ischemic stroke (IS) controls, IS-COVID were less likely to have hypertension, dyslipidemia, or be smokers, but more likely to be male, younger, have diabetes, obesity, acute renal failure, acute coronary syndrome, venous thromboembolism, intubation, and comorbid intracerebral or subarachnoid hemorrhage (all p<0.001). In April-July 2020, comorbid COVID-19 infection was present in 2,086/43,582 (4.8%) of IS patients.  Other comorbidities included: Respiratory failure, acute coronary syndrome, Pulmonary embolus, intracerebal hermorrhage:  Vs IS controls, IS-COVID developed more acute complications, including respiratory failure requiring mechanical ventilation (43.5% vs. 11.8%, p<0.001), acute coronary syndrome (18.3% vs. 8.5%, p<0.001), pulmonary embolus (7.4% vs. 2.0%, p<0.001), and comorbid intracerebral hemorrhage (9.6% vs. 6.6%, p<0.001) | Y | Hospital length of stay was longer for Ischemic stroke (IS)-COVID compared to IS controls (17.7 vs. 7.5 days, p<0.001) | Y | Ischemic stroke (IS)-COVID patients were less likely to receive Alteplase or mechanical thrombectomty: (1.8% vs. 5.6%, p<0.001) and (4.4% vs. 6.7%, p<0.001). | Y |
| Dell'Utri 2020 | Change in fetal deaths was measured: 5 fetal deaths were recorded during the COVID-19 period vs 1 in 2019. (chi square computed using as denominator all observed pregnancies = 4.29, p = 0.04) The frequency of intrauterine fetal deaths diagnosed at admission increased. No p-values. | Y | Emergency Services (ES) admissions decreased by 35.4 % (95 % CI—34.1–36.6). Reduction was more marked for gynecological complaints (-63.5 %, 95 %CI -60.5 to -66.5): in particular we observed a reduction of admissions for genital infection/cystitis of 75.7 % (95 %CI -71.4 to -80.1)  Admission for complaints associated with pregnancy decreased by 28.5 % (95 %CI -27.2 to-29.9). Frequency of admission for elective caesarean section/labor induction increased from 47.5 % in 2019 to 53.6 % in 2020 [statistically significant]. Cases of threat of pretermbirth increased of +13.7 % (95 %CI + 4.1 to +22.3; from 49 to 53 cases). Frequency of natural deliveries decreased. | Y | Admission for complaints associated with pregnancy decreased by 28.5 % (95 %CI -27.2 to-29.9). Frequency of admission for elective caesarean section/labor induction increased from 47.5 % in 2019 to 53.6 % in 2020. No p-values reported. | N | NA | NR |
| DeLuca 2020 | In-hospital mortality increased to 6.8% in 2020 from 4.9% in 2019 (192 deaths, 6.8% vs. 169 deaths, 4.9%, OR: 1.41; 95% CI: 1.15 to 1.71; p < 0.001)  Among COVID-19 positive ST elevation myocardial infarction patients, 18 of 62 positive patients died (29% vs. 5.5%, OR: 7.0; 95% CI: 4.1 to 12.3; p < 0.001). | Y | Outcomes: *1) number of patients with ST elevation myocardial infarction undergoing percutaneous revascularization;* significant reduction in primary percutaneous coronary intervention as compared with 2019 (incidence rate ratio: 0.811; 95% confidence interval: 0.78 to 0.84; p < 0.0001)  2) proportion of patients with ischemia time >12 h; significant increase in total ischemia time in 2020, 328 patients (11.7%) from 316 (9.1%) in 2019 (adjusted p = 0.001)  PPCI for STEMI in patients with arterial hypertension experienced a particularly heightened reduction in primary PCI procedures | Y | NA | N | Proportion of patients with a door-to-balloon time >30 min was significantly longer in the pandemic time period, 1,062 patients (57%) had a door-to-balloon time >30 min in 2019 vs 1,843 patients (52.9%) in 2019.  P = 0.003 | Y |
| DeRosa 2020 | ST Elevation myocardial infarct case fatality rate during the pandemic was substantially increased to 13.7% compared with 2019 4.7% rate [risk ratio (RR) = 3.3, 95% CI 1.7–6.6; P < 0.001]  Case fatality rate among SARS-CoV2-positive STEMIs was substantially higher (28.6%) compared with all other STEMI patients registered during the same week in 2020 (11.9%). No p-values. | Y | 48.4% reduction in admissions for acute myocardial infarct compared with the equivalent week in 2019 (P < 0.001). Significant reduction in ST-elevation myocardial infarction and Non-STEMI admissions during COVID-19: [STEMI; 26.5%, 95% confidence interval (CI) 21.7–32.3; P = 0.009] and non-STEMI (NSTEMI; 65.1%, 95% CI 60.3– 70.3; P < 0.001  Major complications were registered in 18.8% of cases in 2020 and in 10.4% in 2019 (RR = 1.8, 95% CI 1.1–2.8; P = 0.025) | Y | Reduction in admissions [see column H]]: reduction was higher for women (41.2%; P = 0.011) than men (17.8%; P = 0.191). A similar reduction in AMI admissions was registered in North Italy (52.1%), Central Italy (59.3%), and South Italy (52.1%)  319 acute myocardial infarction were registered during the 2020 week, with a 48.4% reduction (95% CI 44.6–52.5) compared with the equivalent week in 2019 (P < 0.001), when 618 patients were hospitalized for the same diagnosis 82 patients were hospitalized for HF during the 2020 week, compared with 154 during the equivalent week in 2019 (46.8% reduction, 95% CI 39.5–55.3; P = 0.005)  Substantial reduction in hospitalizations was noted for atrial fibrillation. A total of 41 AF-related hospitalizations were registered during the 2020 week, with a 53.4% reduction (95% CI 43.9–64.9) compared with the equivalent week in 2019 (P =0.017), when 88 patients were hospitalized for the same diagnosis. The mean age of AF patients was 70.0 ± 7.5 years in 2019 and 64.6 ± 12.3 in 2020 (P = 0.139). Finally, a 29.4% reduction (95% CI 0.45–0.89) was registered for DF (P = 0.349) while a 63.2% reduction (95% CI 0.14–0.61) was found for PE (P = 0.667) | Y | Both patient- and system-related declared delays were substantially increased during the COVID-19 outbreak. In fact, the time from symptom onset to coronary angiography was increased by 39.2% in 2020 compared with the equivalent week in 2019, while the time from first medical contact to coronary revascularization was increased by 31.5%. Rate of Primary Percutaneous Coronary Intervention dropped significantly among Non-ST elevation myocardial infarct patients  No P value | NR |
| D'Urbano 2020 | Fatality rate after surgery: during the pandemic decreased to (11.1) compared to the (19.6) rate recorded in 2019. No p-values.  COVID-19 mortality: 1 patient tested positive for COVID-19 and died. | NR | The complication rate during the pandemic increased substantially to 15 out of 27 cases (55.5), compared to 17 out of 46 cases (36.9) recorded in 2019  Type of surgery, postoperative complications and postoperative hospital stay. Postoperative complications were defined as wound infections, pneumonia, bleeding and anastomosis leak | N | Overall (41.3) reduction in the number of patients who were hospitalized and underwent emergency surgery during the lock down period. The mean length of hospitalization in 2019 was 16.5 d, ranging from 1 to 53 d, while in 2020, it was 12.9 d, ranging from 3 to 35 d. No p-values reported.  Total postoperative hospital length of stay (d)  2019: 16.5 (1-53)  2020: 12.9 (3-35) | NR | NA | NR |
| Egol 2020 | The COVID-19 positive hip fracture cohort (C+) had an increased mortality rate compared to the suspected COVID-19 cohort and negative cohort; 35.3% vs. 7.1% vs. 0.9%  53% of C+ and 14% of COVID-19 suspected patients (Cs) died by 30 days compared with 5.6% of COVID negative (C--) patients | Y | A comparison of the hip fracture cohort (C+) and Cs groups [lab test positive C+ and suspected COVID-19 Cs] vs the COVID negative (C--) hip fracture patients found: Greater in-hospital and 30-day mortality rates , a greater length of hospital stay, a greater major complication rate and a greater need for ventilators post-operatively   Complications were seen at a significantly higher rate in those with or suspected of having COVID than those not. The incidence of pneumonia (70.5% vs. 35.7% vs. 0.9%, p < 0.001), respiratory failure (41.2% vs. 28.6% vs. 1.9% p < 0.001), sepsis (17.6% vs. 7.1% vs. 2.8%, p = 0.033), and death (35.3% vs. 7.1% vs. 0.9%, p - 0.001) was significantly elevated in the C+ group compared T4 with the Cs and C2 cohorts  *Significant P values reported, others were insignificant - deep vein thrombosis/pulmonary embolism, myocardial infarct, stroke, acute renal failure, UTI, anemia | Y | NA | N | The positive hip fracture cohort (C+) group was delayed to surgery compared with the negative hip fracture (C--) patients by 1 day | N |
| Eshraghian 2020 | NA | NR | Significant decrease in the number of hospital admissions for liver-related disorders compared with the previous years control period at a large hepatobiliary referral centre  Number of admissions for each cause of liver-related complications was also reduced. Model for End-Stage Liver Disease (MELD) scores for patients with liver cirrhosis admitted to hospital and the mean duration of hospital stay were higher during the COVID-19 outbreak than the control period  Changes in Morbidity/Complications All Admissions (rate per day) Study period: 124 (1.74) Control: 230 (3.23)  Incidence rate ratio: 1.85 (1.49- 2.30)  Gastrointestinal Bleeding:  Study period: 14 (0.19)  Control: 35 (0.49) Incidence rate ratio: 2.50 (1.34- 4.64)   Spontaneous bacterial peritonitis: Study period: 19 (0.26)  Control: 34 (0.47) Incidence rate ratio: 1.78 (1.02- 3.13)  Hepatorenal syndrome: Study period: 23 (0.32)  Control: 45 (0.63)  Incidence rate ratio: 1.95 (1.18- 3.23)   Hepatic Encephalopathy Study period: 19 (0.26) Control: 37 (0.52) Incidence rate ratio: 1.94 (1.12- 3.38)   Acute Hepatitis: Study period: 6 (0.08)  Control: 14 (0.19) Incidence rate ratio: 2.33 (0.89- 6.07)  Diuretic Resistant Ascites Study period: 7 (0.09)  Control: 16 (0.22) Incidence rate ratio: 2.28 (0.94- 5.55)   Liver transplant related admissions Study period: 36 (0.50)  Control: 49 (0.69) Incidence rate ratio: 1.36 (0.88- 2.09)    Liver transplant related admissions Study period: 36 (0.50)  Control: 49 (0.69) Incidence rate ratio: 1.36 (0.88- 2.09) | Y | Reported significant decrease in the number of hospital admissions for liver-related disorders during the COVID-19 pandemic vs control, and a reduction in admissions for each cause of liver-related complications.  Mean duration of hospital stay was 7.44 days (SD 5.42) during the study period compared with 5.32 days (3.37) during the control time period (p<0.001)  Mean rate of hospital admissions for liver-related disorders during the study period was 1.74 admissions per day (SD 0·95) compared with 3.23 admissions per day (1·33) during the control period (incidence rate ratio [IRR] 1·85, 95% CI 1·49–2·30; p<0·001) | Y | NR | NR |
| Fadel 2020 | 53% of C+ and 14% of COVID-19 suspected patients (Cs) died by 30 days compared with 5.6% of COVID negative (C--) patients | Y | This paper reports on changes to ICU admissions for non-COVID-19 admissions and outcomes. A total of 21 different diagnoses on admission were compared. Demographics, ICU admission sources, hospital and ICU length of stay (LOS), hospital and ICU mortality, admission acute physiology score (APS), acute physiology and chronic health evaluation (APACHE III) score, and admission principal diagnosis to the ICU  Findings: Decrease in patient counts for all admission diagnoses was noted except for cardiogenic shock and the acute respiratory distress syndrome (ARDS). Non COVID-19 ICU admissions had a statistically significant higher APS score and APACHE III score in 2020 compared to 2019 (P < 0.0001 for both). Proportionally more patients were admitted with acute respiratory distress syndrome (P = 0.0041), sepsis (P = 0.0193), cardiogenic shock (P = 0.002), respiratory failure on mechanical ventilation (P < 0.0001), and patients on chronic dialysis (P = 0.0323).   There were proportionally fewer chronic obstructive lung disease (COPD) exacerbations (P = 0.0003), chest pain admissions (P = 0.0142), and post-operative surgical patients (P = 0.0004). Despite higher acuity at presentation, there were no statistical differences in ICU or hospital mortality within the Cleveland Clinic healthcare system. No differences in mortality for non-COVID-19 patients at the county level in Northeast Ohio were seen | Y | ICU Length of Stay (Mean) 2019: 3.0 (4.5) 2020: 2.7 (3.4) P < 0.0001  Decrease in presentation to hospital: A decrease of 40.5% from 67,217 during the same period last year, with incidence rate ratio (IRR): 0.5946 (95% CI: 0.5873–0.6020).  The number of patients presenting at all 10 hospital ERs from March 15 to April 30 2020 was 39,970, With universal COVID-19 testing for all admissions, ICU admissions for non-COVID-19 cases decreased by 38.1% from 2573 to 1592, IRR: 0.6187 (95% CI: 0.5812– 0.6586). During the above same period the total number of ICU admissions of COVID-19-confirmed cases was 274, and the total overall number of hospitalised COVID-19-confirmed patients was 656 | Y | NA | NR |
| Frankfurter 2020 | In-hospital mortality: 12.5% vs 6%, p=0.046 | Y | NR | NR | Hospitalization acute decompensated heart failure: 107/128 patients (84%) vs. 149/186 (80%); overall ED visits: +38.3% (26.3%-51.6%, p<0.001), ADHF-related ED visits: -43.5% (14.8%-79.4%, p=0.002); ADHF-related hospitalizations: -39.3% (8.6%-78.5%, p=0.009). Post-Ontario lockdown (more significant): ED visits: +44.0% (29.4%-60.3%, p<0.001), ADHF-related ED visits: -79.2% (34.3%-139.0%, p<0.001), ADHF-related hospitalizations: -64.5% (20.0%-225%, p=0.002) | Y | NR | NR |
| Friedman 2020 | Excess out-of-hospital deaths 2020: 194.7 deaths (95%CI: 135.5-253.9), +145% (70%-338%) vs. 2019, p-value not reported | NR | NR | NR | NR | N | Non-urgent cases (2020 vs. 2019): 39.0% vs. 59.1%, Urgent cases: 11.2% vs. 6.7%, Deceased cases: 20.0% vs. 7.9%, Ambulance arrival-on-scene time: 20.5 mins vs. 16.4 mins | N |
| Giannouchos 2020 | NR | NR | NR | NR | Inpatient ED visits: 8089 vs. 8562, -5.5% decline. No p-values reported. | NR | Outpatient ED visits: 30276 vs. 32927, -8.1% decrease. No p-values reported | NR |
| Gluckman 2020 | All acute MI mortality (unadjusted): Late period (odds ratio (OR): 1.02 [95% CI: 0.77-1.35]) vs. early (OR: 0.95 [0.69-1.31]) vs. reference, All acute MI mortality (adjusted): Late period (OR: 1.44 [95% CI: 0.86-1.53]) vs. early (OR: 1.02 [0.74-1.42]) vs. reference | N | NR | NR | AMI hospitalization rate: –19.0 cases (95% CI, –29.0 to –9.0) per week - for 5 weeks (early COVID-19 period - Feb 23 - Mar 28, 2020), then increased +10.5 (95% CI, +4.6 to +16.5) cases per week, marking the late COVID-19 period (Mar 29 - May 16, 2020). Median (IQR) length of stay for patients with AMI was shorter in the early COVID-19 period by 7 hours and in the later COVID-19 period by 6 hours compared with the before period (56 [41-115] hours and 57 [41-116] hours vs 63 [43-122] hours, respectively; P < .001) | Y | NR | NR |
| Goksoy 2020 | NR | NR | NR | NR | Inpatient surgical treatment: 41 (48.8%) vs. 49 (38.9%), p = 0.155, Length of hospital stay: 2.99 days (2.28) vs. 4.00 (3.27), p = 0.021 | Y | NR | NR |
| Gramegna 2020 | 4 deaths (15.4%) vs. 2 deaths (9.5%), p = 0.69 | N | Thromboembolism: 5 (19.2%) vs. 1 (4.8%), p = 0.02, Mechanical ventilation: 5 (19.2%) vs. 4 (19.0%), p = 0.99, Inotropic support: 7 (26.9%) vs. 4 (19.0%), p = 0.73, Mechanical circulatory support: 5 (19.2%) vs. 4 (19.0%), p = 0.99, Continuous renal replacement therapy: 2 (7.7%) vs. 0 (0.0%), p = 0.49 | Y | ICU length of stay: 3 days (1-6) vs. 3 (1-4), p = 0.72, Hospital length of stay: 4 days (3.0-7.8) vs. 9 (7-14), p < 0.01 | Y | Time from symptom onset to hospital: 15 hours (2-48) vs. 2 (1-3), p < 0.01, Late-presentation ST-elevation myocardial infarction: 13 (50%) vs. 1 (4.8%), p < 0.01, Door-to-perfusion: 60 mins (20-120) vs. 40 (30-60), p = 0.22, Procedural time: 72.5 mins (60-100) vs. 60 (45-90), p = 0.07 | Y |
| Grewal 2020 | COVID-AIS (Acute Ischemic Stroke): 2 patient (15.3%), p-value not reported | NR | Discharge modified Rankin score (mRS): COVID-AIS: 4 (3-4) vs. AIS 2020: 3 (1-4) vs. AIS 2019: 3 (1-4), p = 0.050, Discharge mRS > 2: 76.9% vs. 47.2%, 40.9% (p = 0.047), correction for age and sex in a logistic regression model [odds ratio (OR): 3.82, (CI 1.02–14.3), p = 0.046] | Y | NR | N | NR | N |
| Gul 2020 | NR | NR | Hydronephrosis grade at admission: None: 5 (14.3%) vs. 25 (21.9%), Grade 1: 10 (28.6%) vs. 40 (35.1%), Grade 2: 12 (34.3%) vs. 37 (32.5%), Grade 3: 5 (14.3%) vs. 9 (7.9%), Grade 4: 3 (8.6%) vs. 3 (2.6%), p = 0.325 | N | Hospital length of stay: 10.8 days (5.5) vs. 6.2 (3.5), p = 0.045 | Y | European Association of Urology Priority Group: Low: 13 (37.1%) vs. 35 (39.5%), Intermediate: 8 (22.9%) vs. 46 (40.4%), High: 6 (17.1%) vs. 16 (14%), Emergency: 8 (22.9%) vs. 7 (6.1%), p = 0.019 | Y |
| Gupta 2020 | NR | NR | Total infection rate: 15 (25.42%) vs. 21 (20.79%), p = 0.05, External fixator application: 42 (71.18%) vs. 51 (50.49%), p = 0.005, Readmission rate within 30 days: 7 patients (13.46%) vs. 10 (11.23%), p = 0.79 | Y | Direct admissions: 19 patients (36.53%) vs. 45 (50.56%), p = 0.20, post-operative length of stay: 3.12 days (3-21) vs. 3.34 (3-27), p = 0.81 | Y | Delay from injury to presentation in emergency/administration of antibiotic: 6.75 hours (1-15) vs. 4.04 (1-14), p < 0.0001, Delay from admission to surgery: 24.04 hours (8-96) vs. 19.32 (6-72), p = 0.15 | Y |
| Habonimana 2020 | Deaths (Jan - May 2020 vs. 2019): 170 (16.4%) vs. 181 (17.4%), p = 0.52, Deaths (May 2020 vs. 2019): 44 (21.9%) vs. 28 (13.6%), p = 0.028, Time-to-death (2020 vs. 2019): 6.7 days (8.9) vs. 7.8 days (10.9) | Y | NR | NR | Admissions: 1037 vs. 1038. No p-values reported. | NR | NR | NR |
| Huang 2020 | All-cause mortality: 6 (11.3%) vs. 2 (3.8%), p = 0.14 | N | Cardiogenic shock: 3 (5.7%) vs. 3 (5.7%), p = 0.98, Major bleeding: 0 (0%) vs. 0 (0%), p = NS, Cardiac rupture: 3 (5.7%) vs. 1 (1.9%), p = 0.30 | N | Hospital length of stay: 14 days (10-15) vs. 13 (10-14), p = 0.37 | N | Delay time (STEMI): 68 mins (61-76) vs. 40 (37-45), p < 0.001 | Y |
| Jacob 2020 | 3/97 vs 3/126, p-value not reported (not significant) | N | Complications: 3/97 vs 9/126 | N | N/A | N | N/A | N |
| Jacobson 2020 | Multiple weekly death rates by age: not significant | N | NR | NR | N/A | N | N/A | N |
| Jasne 2020 | Hospice/deceased: 27/211 vs 19/167, p-value not reported (not significant) | N | Door to needle time: 63 (45 - 105), 57 (44-75); Discharge mRS (0-2): 97/211, 78/167 | N | N/A | N | N/A | N |
| John 2020 | In-hospital mortality (ischemic stroke): 3.1% vs. 0.9% (p = 0.40), [hemorrhagic stroke]: 9.5% vs. 3.8% (p = 0.64) | N | N/A | NR | Total stroke: 130 vs. 109 (p=0.174), Ischemic stroke: 103 vs. 76 (p = 0.044), Hemorrhagic stroke: 27 vs. 33 (p = 0.44) | N | Ischemic stroke: Last known well to door: 517 mins (557) vs. 620 (744), p=0.293, Door-to-Needle (IV thrombolysis): 43 mins (15) vs. 36 (13), p=0.17, Door-to-groin (endovascular thrombectomy): 104 mins (33) vs. 68 (20), p =0.001 | Y |
| Kastritis 2020 | Dead on arrival: 7 patient vs. 9 patients (p-value not reported) | NR | NR | NR | Hospitalizations: 33.2% vs. 29.3%, p = 0.09, ICU admissions: 25 (3.8%) vs. 31 (2.7%) | N | NR | NR |
| Katsouras 2020 | NR | NR | NR | NR | Stroke admissions: incidence rate ratio [IRR]: 0.49 [95% CI: 0.33-0.74, p = 0.001], -51% reduction, Acute coronary syndrome admissions: IRR: 0.73 [95% CI: 0.58-0.92, p = 0.009], -27% reduction | Y | NR | NR |
| Keizman 2020 | In-hospital mortality: 14 (13%) vs. 9 (5.2%), p = 0.037 | Y | Revision: 0 (0.0%) vs. 8 (4.7%), p = 0.058, Stroke: 0 (0%) vs. 0 (0%), p = 1.0, Acute Kidney Injury: mild: 104 (97.2%) vs. 170 (98.8%), moderate: 3 (2.8%) vs. 1 (0.6%), severe: 0 (0.0%) vs. 1 (0.6%), p = 0.233, acute respiratory distress syndrome: 2 (16.7%) vs. 1 (3.3%), p = 0.39, atrial fibrilllation: 20 (22.2%) vs. 48 (27.9%), p = 0.40, heart failure: 1 (8.3%) vs. 1 (3.3%), p = 1.0, Pacemaker: 0 (0.0%) vs. 1 (3.3%), p = 1.0, Wound infection: superficial: 2 (2.2%) vs. 8 (4.7%), deep: 1 (1.1%) vs. 0 (0.0%), p = 0.24 | N | Ventilation: 4070.48 mins (9294.47) vs. 2721.28 (7779.54), p = 0.23, Intensive care time: 95.27 hours (180.40) vs. 71.43 (160.64), p = 0.30, Hospital time: 9.84 days (6.82) vs. 9.95 (8.39), p = 0.92 | N | NR | NR |
| Khalil 2020 | Stillbirths: 16 (9.31%) vs. 4 (2.38%), Difference: 6.93 (95% CI: 1.83-12.0), p = 0.01 | Y | Preterm birth: Prior to week 34: 62 (3.7%) vs. 42 (2.5%), p = 0.07, Prior to week 37: 127 (7.6%) vs. 113 (6.8%), p = 0.46, Caesarian delivery: 419 (24.8%) vs. 423 (25.6%), p = 0.60, | N | Admission to neonatal unit: 106 (6.2%) vs. 103 (6.1%), p = 0.94 | N | NR | NR |
| Laskar 2020 | 30-day mortality rate: 2 (1.2%) vs. 6 (2.2%), P = 0.72, odds ratio (ORs): 1.9 [95% CI: 0.38-9.54] | N | NR | NR | Unplanned 30-day readmissions: 16.7% vs. 12.6%, p = 0.23, hospital length of stay: 4.1 days (5.9) vs. 3.9 (8.9), p = 0.80 | N | Planned follow-ups: 36.2% vs. 49.1%, p < 0.01, odds ratio: 1.7 [95% CI: 1.15-2.51] | Y |
| Lau 2020 | In-hospital mortality: 11.7% vs 11.2%, P = 0.063 | N | NR | NR | Intensive care unit admission rate (5.1% vs 5.3%, P = 0.22) | N | Daily admissions: –178.27 (95% CI: –263.70 to –92.85; P < 0.001), 17% reduction, Surgeries: 4704 vs. 5844, Emergency surgeries: 8.0% vs. 6.7%, p < 0.001, Endoscopies: 9477 vs. 11604, Emergency endoscopies: 5136 (18.2%) vs. 4675 (14.0%), p < 0.001 | Y |
| Lauridsen 2020 | Mortality: 45% (95% CI: 32-58%) vs. 36% (95% CI: 31-41%), age/sex/cormorbidity adjusted: odds ratio (OR): 1.04 (95% CI: 0.68-1.59) | N | Intra-aortic baloon pump: 0 (0%) vs. <3, p > 0.05, left-ventricular assist device: <3 vs. 44 (13%), p < 0.05, extracorporeal membrane oxygenation: 4 (7%) vs. 26 (8%), p = 1.0, renal replacement therapy: 8 (13%) vs. 48 (14%), p = 1.0, mechanical ventilation: 38 (63%) vs. 261 (76%), p = 0.05, cardiogenic shock: 119 (5.6%) vs. 696 (5.9%) | Y | NR | N | NR | N |
| Leitinger 2020 | NR | NR | N | NR | NR | N | NR | N |
| Lerner 2020 | Proportion of scene deaths nearly doubled, increasing from 1.49% to 2.77% among all EMS activations with patient contact by week 15 [vs. week 11 2020]  Raw numbers of EMS-attendedscene deaths reported in 2020, the number increases from 6,294 in week 11 to 8,942 in week 15  No p value provided | NR | N | NR | NR | N | NA | N |
| Leung 2020 | Reported as a time trend (per year) and by Covid-19 pandemic using aRR/aOR ratio   Mortality rate Time trend (per year) 0.88 95%CI: 0.65–1.18 p = 0.381 COVID-19 pandemic 1.54 95%CI: 0.29–7.09 p= 0.588 | N | NA | NR | NR | N | The background yearly trend was relatively stable (Fig. 1) (adjusted relative risk (aRR): 0.98, 95% confidence interval (CI): 0.95–1.00, P = 0.09). An evident drop in acute ward admissions for seizure was noted in the study period during the COVID-19 pandemic (aRR: 0.70, 95% CI: 0.60–0.80, P<0.001)  Significant reduction for accident and emergency department was demonstrated in the study period (aRR: 0.78, 95% CI: 0.65–0.92, P = 0.003) on the top of background gradual declining trend (aRR: 0.95, 95% CI: 0.92–0.99, P =0.005).  Ratio of acute ward admissions for seizures per A&E attendance did not change significantly within the study period (aRR: 1.27, 95% CI: 0.81–2.01, P=0.31) .  LOS in acute general ward (length of stay) aRR/aOR (adjusted relative risk/ adjusted odds ratio). Time trend (per year) 1.05 (95 CI% 1.03–1.08), p=<0.001) COVID-19 pandemic: 0.80 (95% CI: 0.70–0.93, p= 0.003)  LOS in ICU/HDU (N = 87) Time trend (per year) 0.94 (95% CI: 0.80–1.10, p =0.41) COVID-19 pandemic 0.94 (95% CI: 0.46–1.94, p=0.87) | Y |
| Li 2020 | NA | NR | NA | NR | NR | N | Despite similarities in the site of occlusion and successful recanalization rates between the two groups. Median time from symptom onset to recanalization was 132 minutes longer during the pandemic compared with the previous year (672 vs. 540 min, P = 0.049)  NICU stay was longer during the pandemic (10 d vs. 7d, P = 0.013)  Endotracheal intubation and mechanical ventilation were more common among patients receiving GA during the pandemic than in 2019 (84.6% vs. 42.4% P<0.05). higher incidence of delayed extubation after emergent endovascular treatment (EVT) (69.2% vs. 45.5%, P<0.05)  Moreover, of the patients receiving general anesthesia (GA) during the pandemic, 69.2% experienced delayed extubation compared with 45.5% in 2019 (P=0.146). | Y |
| Little 2020 | No significant in-hospital all-cause mortality (10.9% (n=38) in 2020 vs 8.6% (n=38) in 2019, p=0.28)  Increased mortality in COVID -19 vs non- COVID 19 ST-Elevation Myocardial Infarction (STEMI) (21.7% (10) vs 9.3% (28) OR =2.2, CI 1.25-5.82 p =0.012) for patient having primary percutaneous coronary intervention (PPCI) | Y | 46 patients with active COVID-19 were more thrombotic and more likely to have intensive care unit admissions (32.6% (15) vs 9.3% (28), OR 5.74 (95%CI 2.24 to 9.89), p<0.001)  No significant difference in ICU admission (10.6% (37) in 2020 vs 9.8% (43) in 2019, p=0.69) | Y | NR | N | First call to door time was significantly longer in the 2020 cohort compared with the 2019 cohort (median (IQR) 87 (62–118) min vs 75 (57–95) min, p<0.001  Not associated with a delays in achieving revascularisation once in hospital (48 (34–65) min in 2020 vs 48 (35–70) min in 2019), p=0.35  COVID 19 vs non-COVID 19 STEMI  Door to balloon time was significantly increased for COVID-19 positive patients (51 (39–77) min vs 47 (32–63) min, p=0.026)  COVID -19 patient had longer in- hospital length of stay median/IQR : (4 (3–9) days vs 3 (2–4) days, p<0.001) | Y |
| Li 2020 | N | NR | N | NR | NR | N | The symptom onset-to-door time increased by 27% [(142 [75 to 338] vs 180 [84 to 460] min (p <0.01)] in 2020 compared with the equivalent months in 2019) | Y |
| Luostarinen 2020 | 30-day all cause mortality (multivariable logistic regression based on patients in 2019 and tested on patients admitted in 2020), performance of model used area of the curve (AUC) of the receiver operating characteristics with 95% confidence interval. Compared using t-test  AUC of the predicted risk of death model was 0.88 (95% CI, 0.82–0.94), including all TBI and SAH patients admitted in 2020.  Traumatic brain injury (TBI) patients admitted in 2020, the AUC was 0.85 (95% CI, 0.76–0.94) Aneurysmal subarachnoid hemorrhage (SAH) patients admitted in 2020, the AUC was 0.90 (95%CI, 0.81–0.98).  Standardized mortality rates (SMR) - calculated as 30-day mortality. The expected risk of 30-day mortality is adjusted for age, Glasgow Coma Scale score on admission, and pupillary light reactivity. SMRs are compared using a t test  Was no notable difference in SMRs for patients treated in the early and late intervals in 2020 compared with 2019 for any of the patient groups (p > 0.05 for all comparisons)  Early interval SMR for TBI p =0.754, late interval SMR for TBI p=0.566 Early interva lSMR for SAH p =0.330, late interval SAH for SAH p=0.691 | Y | NA | NR | NR | NR | Traumatic brain injury patients admitted in the late interval in 2020 underwent craniotomy or decompressive craniectomy less frequently than patients admitted in the early interval (61 vs. 35%, p = 0.038)  No difference in days in ICU (see below)  Traumatic brain injury (TBI) -   Median (IQR) - (2019 n= 56 - 4 (2-7), 2020 n= 67 - 3 (2-8) p=0.869)  Aneurysmal subarachnoid hemorrhage (SAH)  Median (IQR) - (2019 n= 49 - 8 (4-14), 2020 n= 52 - 9 (3-13) p=0.923) | Y |
| Lv 2020 | There were 1 male patient and 3 female patients, with an average age of 69.3 ± 15.5 years (range, 51 to 91). The mortality rate was 0.46% and the timing of death was 6.3 ± 2.3 d (range, 4 to 9) after injury.  In the control group, there were 7 patients died during hospital stay, including 5 male patients and 2 female patients with an average age of 26.9 ± 17.9 years (range, 6 to 61) mortality rate 0.43%  No p value reported | NR | NA | N | NR | N | The proportion of minimally invasive surgery in the epidemic group (45. 0%, 361/805) was significantly higher than that (34.8%, 554/1595) of the control group ( χ2 = 23.449, P < 0.01). | Y |
| Madanelo 2020 | NA | NR | N | NR | 46.4% fewer patients visited urological ED (122 in 2020 vs. 263 in 2019). (no p value reported)  Percent of patients requiring hospitalization was 11% in 2019 and 18.9% in 2020, p<0.05 | Y | The percentage of patients who required emergency surgery and hospitalization was 6.8% and 11.0% in 2019, respectively. At the corresponding period in 2020, 9.0% required emergency surgery (p>0.05) and 18.9% needed hospitalization (p<0.05) | Y |
| Magnani 2020 | Rate ratio of 1.11 (1.02-1.21 95 CI%) <age 60, 1.55 (1.52-1.58 95% CI) >age 60   RR - computed by dividing mortality rate in 2020 by mortality rate in 2015-2019 reference period  Most regions showed the same ratio of total mortality increase and COVID-19 mortality. The regression function showed slope = 0.47 (p < 0.001) and intercept = 7.8. Valle D’Aosta, with a large amount of swab testing activity, showed a higher COVID-19 deaths frequency compared to all-causes mortality increase.  Extrapolation of 2015-2019 show 45033 excess death, while confirmed COVID -19 was 21046 (no p values but iconfidence intervals used to estimate COVID-19 mortalities) | Y | NA | NR | NR | N | Only described in generalities of pandemic impact | N |
| Magro 2020 | Three (3) deaths in study period vs. 0 in control (4.6%, vs. 0% p = 0.04) Of those that died 1 tested negative for COVID, 2 were not tested | Y | N | NR | NR | N | 7 of 65 patients, 10.8% were lost to follow-up vs. 2 of 76 were lost to follow-up 2.6%, p=0.03  During the study period (2020) six new tuberculosis diagnoses were made (9.2%), significantly fewer than the 15 new diagnoses (19.7%) in the control (2019) period (p=0.04) | Y |
| Malik-Tabassum 2020 | Period A = March 23 to May 11, 2018, Period B = March 23 to May 11, 2019, Period C = March 23 to May 11, 2020  30-day mortality (%) 3.3 vs 10.7 vs. 8.8 (A,B,C) p=0.157 | N | NA | NR | NR | N | period A = March 23 to May 11, 2018, period B = March 23 to May 11, 2019 Period C = March 23 to May 11, 2020  Mean length of inpatient stay – days: Period A, B, C [SD] ( 15.8 [11.4], 16.3 [11.4], 8.6 [4.6]) p = <0.001  Significant reduction in time between admission and orthogeriatric assessment (mean(SD)= 25.7(26.8) h in period C, 39.0h(38.7) in period B, 37.1h(25.9) in period C), p=0.02  Mean length of inpatient stay was shorter (P<0.001), higher proportion of patient discharged to rehab facility within same NHS trust during the period of lockdown (23.5% in period C, 9.5% in period B, and 10% in period A). p=0.002  reduction in the mean time to surgery for hip fracture patients during period C compared to the previous years (21.8 h in period C, 28.2 in period B, 26.5 in period A), though this difference was not found to be statistically significant, p = 0.49 | Y |
| Mannucci 2020 | Non-COVID-19-Imputed Excess mortality (NCIEM) (difference between 2015-2019 mortality vs. 2020)  National value of 22.2, different regions within showed correlation with NCIEM and COVID-19 mortality (r2=0.61, p<0.001), total cases (r2=0.3,p=0.012) and inverse with cases/total tests ratio (r2=0.49,p=0.001) | Y | NA | N | NR | N | NR | N |
| Marijon 2020 | Proportion of patients who had an OHCA and were admitted alive decreased from 22·8% to 12·8% (p<0·0001)  Lower survival rate at hospital admission (odds ratio 0·36, 95% CI 0·24–0·52; p<0·0001) [after adjusting for potential confounders - (sex, age, location, bystander cardiopulmonary resuscitation, use of automatic external defibrillator before EMS arrival, shockable cardiac rhythm, and call answer to arrival delay)  Survival at hospital discharge (3.1% vs 5.4% p=0.0001) | Y | NA | N | NR | N | Increased call answer to arrival time (median = 10.4 vs 9.4 min) p<0.0001  Less shockable rhythm found at EMS arrival during pandemic(9.2% vs, 19.1%, P<0.0001)  less use of an automatic external defibrillator was observed (0·4% vs 3·0%, p=0·0009)  proportion of OHCAs with resuscitation attempt and advanced life support was lower than usual (53·1% vs 66·2%, p<0·0001) | Y |
| Marini 2020 | 30 day - mortality : Pandemic (n=2, 6%), comparator (0%) p=0.498  In hospital mortality: Pandemic (n=1, 3%) comparator (0%) p = 1.000 | N | Procedural complication (pandemic: n=1, 3% vs. n=1, 4%) p=0.868 | N | NR | N | Mean duration of hospitalization (COVID 19-vs comparator of 8.1 [6.7], 9.4 [6.9]) was unchanged p=0.369 | N |
| Mariottini 2020 | The proportion of positive cases for deceased individual for buprenorphine in 2020 was 1.92 and 1.81 times higher, for amphetamine 1.96 and 2.91 times higher and for THC-COOH 2.12 and 1.69 times higher in March and April, respectively.  P value not reported | NR | NR | N | NR | N | NR | N |
| McGuinness 2020 | NR | NR | Overall Barotrauma Rate [COVID-19 Patients: 24% (95% CI 21-28%); COVID Negative patients: 0.5% (95% CI 0-3%, p<0.001 vs the group with COVID-19 infections); Historical Acute Respiratory Distress Syndrome cohort: 11% (95% CI 8-15%, P<0.001 vs the group with COVID-19 infection)] | Y | NR | N | NR | N |
| McLean 2020 | All cause 30-day mortality rates were significantly higher post-lockdown (8.5% vs. 2.9% prior, p = 0.028), but no differences was observed in operative 30-day mortality (1 (6.7%) vs 2 (12.5%), p = 0.999) | Y | Patients admitted post-lockdown had a significantly longer hospital stay and had higher rates of Clavien-Dindo Grade ≥3 complications (p = 0.001), even when mortality was excluded from complications (p = 0.025) | N | NR | N | There was a significant reduction in admissions following the Government's instructions on the March 16, 2020 from a median of 7 to 3 daily admissions (p<0.001) | N |
| Mendlovic 2020 | NR | NR | NR | NR | NR | N | Patients admitted during the pandemic to IM were younger and had shorter mean hospital stay by over 2 days, when compared with the previous 3 years, 7.63 (10.87) vs. 5.52 (5.12), p<0.001. The median length of stay was the same, but the IQR was shortened by 1 day for the patients admitted during pandemic 4(2-8), 4(2-7), p=0.11. | N |
| Mengal 2020 | March-April, 2020 witnessed highest mortality of 5.3% p<0.001. March-April, 2019 had 3.2% p=0.007 mortality. Jan-Feb, 2020 group had 4.1% p=0.182 mortality. | N | Total ischemic time March-April, 2020: 429.25±272 minutes; January-February, 2020: 359.78±148.04 minutes; March-April, 2019: 346.75±207.31 minutes (no p-values reported) | NR | NR | N | NR | N |
| Merkler 2020 | Inpatient mortality was 32% among patients with COVID-19 with ischemic stroke vs 14% among COVID-19 patients without ischemic stroke (P = 0.003) | Y | NR | NR | NR | N | NR | N |
| Mesnier 2020 | In-hospital death occurred in 23 (3%) of 686 patients before lockdown, and in 25 (5%) of 481 patients after lockdown (p=0.12). In-hospital death occurred in 14 (4%) of 331 patients with ST-Elevation Myocardial Infarction (STEMI) before lockdown versus 16 (6%) of 252 patients with STEMI after lockdown (p=0.26), and in nine (3%) of 355 patients with Non ST-Elevation Myocardial Infarction (NSTEMI) before lockdown versus nine (4%) of 229 patients with NSTEMI after lockdown (p=0.34) | N | NR | NR | NR | N | Values reported as Before lockdown, After lockdown. ST-Elevation Myocardial Infarction: Time from symptom onset to admission, min 180 (108–390), 180 (115–363) p=0.70. Non ST-Elevation Myocardial Infarction: Coronary angiography within 24 h of admission 224 (63%), 143 (62%) P= 0.87. Coronary angiography 24–72 h after admission 102 (29%), 57 (25%) P=0.31 | N |
| Meyer 2020 | NR | NR | NR | NR | NR | N | The per-week comparison showed a significant reduction in Emergency Department referrals only in the last week of the period [337 (2020) vs. 515 (2019), p<0.001]. The proportion of women admitted to the delivery unit in active labor was significantly higher in the last three weeks (39.1% vs. 28.2%, p 0.005), and the rate of discharge was inversely correlated (45.8% vs. 56.7%, p=0.01). During February-March 2020, 1,666 women delivered, compared to 1,654 during February-March 2019. A trend was observed toward a lower rate of composite adverse neonatal outcomes in 2020 compared to 2019 (5.8% vs. 7.1%, p=0.08). | N |
| Miles 2020 | Overall survival rate at discharge was worse during the COVID-19 pandemic compared with before the pandemic. Only 3% of patients survived an In-hospital cardiac arrest and were discharged from the hospital during the 2.5 months of the COVID-19 pandemic (compared with 13% in 2019 before the pandemic [P=0.007] or 18.3% survival in 2018). Furthermore, within the COVID-19-positive cohort, only 2% survived to discharge. | Y | There were 125 In-hospital Cardiac Arrests (IHCAs) during a 2.5-month period at the hospital during the peak of the COVID-19 pandemic compared with 117 IHCAs in all of 2019. IHCAs during the COVID-19 pandemic occurred more often on general medicine wards than in intensive care units (46% versus 33%; 19% versus 60% in 2019; P<0.001), were overall shorter in duration (median time of 11 minutes [8.5–26.5] versus 15 minutes [7.0– 20.0], P=0.001), led to fewer endotracheal intubations (52% versus 85%, P<0.001), and had overall worse survival rates (3% versus 13%; P=0.007) compared with IHCAs before the COVID-19 pandemic. | N | NR | N | NR | N |
| Mitra 2020 | Death in Hospital: 9.6% vs 10.5%. P=0.99 | N | NR | NR | NR | N | Values written as State of Emergency period, Comparator period. Time from onset of symptoms to presentation (227 min (93-1193) vs 342 min (119-1220 p=0.24). Onset of symptoms to presentation of over 24h (11 (21.6) vs 14 (24.6) p=0.67. Time to Computed tomography of the brain (52 (21-97) vs 65 (45-90) p=0.11. Time to reperfusion intervention for all eligible stroke and ST-Elevation Myocardial Infarction (65 min (37-78) vs 44 min (39-60) p=0.54. | N |
| Mohamed 2020 | The rate of 30-day death in the overall cohort was 2.8% (n = 7553), the majority of which occurred in hospital (1.9%; n = 5258). The rate of 30-day death declined between 2017 and February 2020 (2.9% to 2.5%; P<.001), primarily driven by lower in-hospital death (2.1% vs 1.5%), before rising again between March 1, 2020 and May 10, 2020 (3.2%) due to higher rates of post-discharge mortality up to 30 days. Overall, both in-hospital cardiac and non-cardiac death rates declined over the study period (2017 to May 2020: cardiac, 1.21% vs 1.00%; non-cardiac, 0.91% vs 0.79%). No p-value reported. Overall, 59.7% (n = 4499) of 30-day deaths were due to cardiac causes. While 30-day cardiac and non-cardiac death rates both dropped between 2017 and February 2020, they were significantly increased in patients undergoing Percutaneous Coronary Intervention between March 1, 2020 and May 10, 2020. No p-value reported | NR | NR | NR | NR | N | NR | N |
| Mohammad 2020 | All cause mortality:Pandemic 2.9%. Control period: 2.3%. P=0.21 | N | NR | NR | NR | N | Values written as Pandemic, Control: ST-Elevation Myocardial Infarction: Time from symptom to Percutaneous Coronary Intervention (PCI) (191 min (116-401), 190 min (122-385)) P=0.45. Time from first Electrocardiogram to PCI (70 min (48-105), 75 (53-119)) P=0.02. High-sensitivity troponin T (2186ng/L (516-5407), 1955 ng/L (596-4853)) P=0.23, PCI (85.5%, 82.4%) P=0.019. Non ST-Elevation Myocardial Infarction: Time from symptom to Percutaneous Coronary Intervention (PCI) (1825 min (1069-2986), 2070 min (1260-3780)) P<0.001, Time from first Electrocardiogram to PCI (1422 min (855-2430) , 1600 min (959-2986)) P<0.001. High-sensitivity troponin T (271 ng/L (99-730), 282 ng/L (109-799) P=0.57. PCI (56.9%, 56.0%) P=0.51 | Y |
| Monti 2020 | NR | NR | NR | NR | NR | N | Duration of symptoms prior to diagnosis: Pandemic: 31 days (14-36). Pre-pandemic: 8 days (6-28). No p value reported. | N |
| Mountantonakis 2020 | Deaths on the scene: Pandemic: 71%. Pre-pandemic: 38%. No p-value reported | N | NR | NR | NR | N | NR | N |
| Moustakis 2020 | NR | NR | NR | NR | NR | N | There was a 44% reduction in the incidence of non-trauma admissions during lockdown (Incidence Rate Ratio (IRR) 0.56; 95% confidence interval (CI) 0.47 - 0.68; p<0.001) and a 53% reduction in the incidence of trauma-related admissions (IRR 0.47; 95% CI 0.34 - 0.66; p<0.001). Even when the prevalence of SARS-CoV-2 infection was minimal, COVID-19 lockdown in North West was associated with a significant reduction in surgical admissions. | Y |
| Mulholland 2020 | NR | NR | NR | NR | NR | N | NR | N |
| Naccarato 2020 | NR | NR | NR | NR | NR | N | Values written as COVID-19, No-COVID-19. Alert-to-admission: 128 min (56-146), 91 min (46-165) p=not significant. Door to needle: 51 min (41-58), 57 min (40-109) p=not significant. Door to groin: 81 min (74-87), 83 min (70-99) p=not significant. Complete stroke work-up: 31%, 69%. P<0.05 | Y |
| Nagamine 2020 | In-hospital mortality of 10.4% vs. 6.3%. P value not reported. | N | National Institutes of Health Stroke Scale (NIHSS) score at presentation, mean ± SD: 10.29 ± 8.5 vs. 9.52 ± 9.8; NIHSS at discharge if the patient survived, mean ± SD: 5.86 ± 5.2 vs. 7.05 ± 8.4. P values not reported. | NR | NR | N | No. of patients with acute stroke symptoms: 48 vs. 64. Mean time from last known well (LKW) to arrival for stroke code patients: 1,041 ± 1682.1 min vs. 554 ± 604.9 min. Of the patients presenting directly to the emergency department (ED) with a known last known well time, 27.8% (10/36) presented in the first 4.5 h vs. 40.5% (15/37). P values not reported | N |
| Nef 2020 | Non-signifcant increase in all-cause mortality of 2.6% (5984 vs. 5832), p = 0.16. Cardiovascular and cardiac mortality increased signifcantly by 7.6%, p = 0.02 and by 11.8%, p < 0.001, respectively. | Y | NR | NR | Number of patients admitted for cardiac catherterization: 2034 vs. 3138, p value not reported. | NR | 44.6% decrease in the number of elective procedures: 1112 vs. 2008; 18.9% decrease in the number of acute coronary syndrome (ACS) related procedures: 860 vs. 1061. P values not reported. | NR |
| Nguyen-Huynh 2020 | In-hospital mortality of 3.8% vs. 4.1%, p=0.74 | N | NR | NR | NR | N | Weekly stroke alert volume decreased to 98 (92-104) vs. 133 (130-136), p<0.0001;  More patients arrived by ambulance: 75.3% vs. 66.6%, p<0.001;  Fewer stroke alert calls from walk in: 18.7% vs. 26%, p<0.001;  Fewer stroke alert calls from inpatient services: 6% vs. 7.3%, p<0.001 | Y |
| Nunez 2020 | In-hospital mortality of 11.1% vs. 4.8% in Period 1, 7.3% in Period 2, 7% in Period 3, p=0.7 | N | NR | NR | Reduction in the number of emergency trauma visits: 512 vs. 1909 in Period 1, 2161 in Period 2, 1983 in Period 3 (p value not reported). Rate of hospital admissions increased to 12.9% vs. 7.6% in Period 1, 6.3% in Period 2, 8.2% in Period 3 (p=0.001).  Number of hospital admissions for osteoporotic hip fracture remained relatively stable at 36 patients vs. 42 patients in Period 1, 41 in Period 2, 43 in Period 3 (p=0.06). | Y | NA | NR |
| Ogliari 2020 | NA | NR | NR | NR | No. of outpatients attending the Fracture Clinic reduced to 182 in weeks 13-19, 2020 vs. 393 in 2015, 400 in 2016, 459 in 2017, 428 in 2018 and 473 in 2019 over the same weeks. A similar reduction was observed also when stratifying by sex or age (all p values < 0.001). In contrast, the mean number of new inpatient admissions for acute hip fracture per week remained unchanged. | Y | NA | NR |
| Okwu 2020 | In-hospital mortality of 5.43% vs. 2.27%, p value not reported. | NR | NR | NR | Number of acute admissions reduced by 58% to 92 vs. 176, p<0.0001.  Fewer patients admitted with renal stones: 27 (29%) vs. 40 (23%), p value not reported.  More patients admitted with haematuria: 35 (20%) vs. 15 (16%), p value not reported.  Fewer patients admitted with urosepsis or urinary tract infection: 8 (9%) vs. 23 (13%), p value not reported.  Fewer patients admitted with cancer-related issues: 13 (14%) vs. 21 (12%). | Y | NA | NR |
| Orellana 2020 | Ratios were calculated between deaths in 2019/2018 and 2020/2019 to estimate excess deaths. No significant excess overall mortality was seen in the ratios for 2019/2018, independently of Epidemiological Week (EW). Meanwhile, the ratios for 2020/2019 increased from 1.0 (95%CI: 0.9-1.3) in EW 12 to 4.6 (95%CI: 3.9-5.3) in EW 17. Excess overall mortality was observed with increasing age, especially in individuals 60 years or older, who accounted for 69.1% (95%CI: 66.8-71.4) of the deaths. The ratios for 2020/2019 for deaths at home or on public byways were 1.1 (95%CI: 0.7-1.8) in EW 12 and 7.8 (95%CI: 5.4-11.2) in EW 17. | N | NR | NR | NR | N | NA | N |
| Padmanabhan 2020 | Thirty-day mortality 11 (10.9%) vs 15 (8.9%), p=0.77 | N | NR | NR | No. of stroke admissions decreased to 101 vs. 167. P value not reported. | NR | NA | NR |
| Pagotto 2020 | NA | NR | NR | NR | NR | N | Total number of procedures reduced to 267 vs. 924 in pre-2020 period, 1036 in post-2019 period (p=0.0002), and 960 in pre-2019 period (p=0.2260). Proportion of reconstructive procedures increased to 95.1% vs. 80.2% in post-2019 period, p=0.00002. Number of outpatient visits decreased to 1571 vs. 3603 in pre-2020 period, 3907 in the post-2019 period (p=0.0003), and 3723 in pre-2019 period (p=0.4495) | Y |
| Papafaklis 2020 | In-hospital mortality of 3.3% vs. 2.7%, p=0.52.  In-hospital cardiac death rate of 2.6% vs. 2.4%, p=0.74 | N | Left ventricle systolic impairment (defined as ejection fraction <40%) at acute coronary syndrome presentation was registered more frequently: 22.2% vs 15.5%; odds ratio: 1.56, 95% CI: 1.22-1.99, P < 0.001. | Y | Acute coronary syndrome (ACS) admissions in the COVID-19 (n = 771) compared with the control (n = 1077) period were reduced overall (incidence rate ratio [IRR]: 0.72, P < .001) and for each ACS type (ST-segment elevation myocardial infarction [STEMI]: IRR: 0.76, P = .001; non-STEMI: IRR: 0.74, P < .001; and unstable angina [UA]: IRR: 0.63, P = .002).  The decrease in STEMI admissions was stable throughout the COVID-19 period (temporal correlation; R2 = 0.11, P = .53), whereas there was a gradual decline in non-STEMI/UA admissions (R2 = 0.75, P = .026) following the progressively stricter social measures. | Y | NA | NR |
| Patel 2020 | NA | NR | NR | NR | Fewer patients sought treatment within 1 day of symptoms: 16/80 patients (19.5%) vs. 41/106 patients (36.9%) in 2019; P=0.005 | N | 82 patients sought treatment for primary rhegmatogenous retinal detachment (RRD) during the 2020 pandemic period, whereas 111 and 103 patients sought treatment for primary RRD during the corresponding 2019 and 2018 control periods, respectively. P values not reported. | NR |
| Patel 2020 | 7% vs. 3%. P value not reported. | NR | NR | NR | No. of patients seen by the general surgery team: 75 vs. 151, 50.3% reduction, p value not reported. No. of patients admitted: 48% vs. 66%, p=0.01. Length of hospital stay: 0 (0-3) vs. 2 (0-4), p=0.04. | Y | NA | NR |
| Pathare 2020 | Increase in total number of suicides and attempted suicides by 67.7% to 220 vs. 369. Compared to 2019, suicides reported during lockdown were by older individuals, more likely to belong to age groups of 31–50  years vs < 30  years in 2019 (χ2 8.2, p < 0.05), signifcantly more men’s suicides were reported during the lockdown (71.2% vs 58.7%, χ2 8.4, p < 0.01), suicide cases were signifcantly more likely to be married (77.7% vs 49%, χ2 28, p < 0.01) and suicides during lockdown were signifcantly more likely to be employed (82.9% vs 59.5%, χ2 21.1, p < 0.01). | Y | NR | NR | NR | N | NA | N |
| Patt 2020 | NA | NR | NR | NR | Cancer-related hospitalizations declined in March (-30%), April (-41%), May (-36%), June (-31%), and July (-38%) as compared to the same months in 2019. P values not reported. | NR | Significant decreases in screening for breast, colon, prostate, and lung cancers were observed in 2020 compared with 2019, with the most reduction occurring in April for mammograms (-85%) and lung (-75%), colon (-74%), and prostate (-56%) screenings. Reductions in cancer biopsies were observed in both April and July for breast (-71% and -31%), colon (-79% and -33%), and lung (-58% and -47%) biopsies. Utilization reductions were observed in patient evaluation and management (E&M) visits, with the greatest reduction in April hospital outpatient E&M visits (-74%). Drops in utilization were higher for new patient E&M visits (-70% in April) than established patient E&M visits (-60% in April). Even with the expanded use of telemedicine, the delivery of E&M services via telehealth was only able to mitigate the drop in E&M utilization in April from -73% to -58%. [P values not reported] | NR |
| Perkin 2020 | Total deaths: 379 vs. 194 (p<0.001); Non-COVID weekly average deaths: 23 vs. 32 (p=0.03); men were more likely to have died of COVID-19 vs. non-COVID-19 in the pandemic study group: 67% vs. 53% (p<0.001); non-white ethnic groups over-represented among COVID deaths vs. non-COVID deaths in the pandemic period (69% vs. 58%, p<0.001); COVID-19 deaths in 2020 were more likely to have occurred in those living in the top five deciles of deprivation vs. non-COVID-19 deaths (44% vs. 38%, p=0.02). | Y | Multiple comorbidities were significantly more likely in those dying from COVID-19 or non-COVID causes in 2020, compared with deaths in 2019.  Diabetes mellitus was significantly more common among patients who died with COVID-19 in 2020 (33% (79/243), p<0.001), compared with deaths in 2019 (16% (32/194)).  Hypertension was more prevalent in both the COVID-19 mortality group (53% (129/243), p=0.02) and the non- COVID deaths in 2020 (55% (75/136), p=0.02), compared with deaths in 2019 (42% (81/194)).  Pre-existing ischaemic heart disease was significantly more prevalent among the non-COVID 2020 deaths compared with the deaths in 2019 (32% (44/136) vs 13% (26/194), p<0.001) | Y | NR | N | NA | N |
| Piccininni 2020 | Monthly all cause mortality of 154.4 per 1000 person years in March 2020 vs. around 10 per 1000 person years between Jan 2012 and Feb 2020. P values not reported. | NR | NR | NR | NR | N | NA | N |
| Pintado 2020 | NR | NR | NR | NR | Post- 'state of emergency' compared to pre- 'state of emergency' announced in Peru: - patient visits: 450 vs. 2213 (declined by 79.7%, no p-value given) - hospital admission rates: 27.3% vs. 8% (p<0.001) - Reasons for hospital admissions changed (hip fracture- 29% vs. 18%; hand fracture- 4.1% vs. 11.2%; foot fracture- 0.8% vs. 6.2%; diabetic foot- 11.4% vs. 2.2%; all are p<0.05) | Y | NR | NR |
| Popovic 2020 | COVID period vs. non-COVID period: greater in-hopsital mortality (8.4% vs. 4.3%, p=0.07)  COVID infected patients vs. non infected: greater in hospital mortality (27.3% vs. 5.6%, p=0.16) | N | COVID period vs. non-COVID period: greater rates of post-op distal embolization (36.1% vs. 15.9%, p<0.01)  COVID infected patient vs. non infected: greater rates of thrombotic myocardial infarction non-atherosclerotic coronary occlusion (54.5% vs. 6.9%, p<0.001), greater rates of post-op distal embolization (72.7% vs. 30.6%, p=0.04), lower left ventricular ejection fraction (LVEF) (<35% LVEF- 45.5% vs. 19.7%; 35-45% LVEF- 27.3% vs. 37.5%; >45% LVEF- 27.3% vs. 52.8%; p=0.02 via Chi-Square test) | Y | NR | N | COVID period vs. non COVID period: delayed first symptoms to balloon time (7.4 ±7.7hrs vs. 3.8 ±3hrs, p<0.001), no significant difference in other times (call to balloon, door to balloon) | N |
| Pop 2020 | NR | NR | NR | NR | Stroke unit admissions (COVID vs. non COVID): -0.6% (no p-value given)  Stroke alerts: 39.6% fewer (174 vs. 288, no p-value reported) | NR | Acute revascularization treatments: 40.9% fewer intravenous thrombolysis and 27.6% fewer mechanical thrombectomy (p=0.034)  Treatment time delays: no significant difference in symptom-to-hospital/hospital-to-intervention/symptom-to-intervention times | Y |
| Quaquarini 2020 | 4/469 (0.85%) patients died of COVID-19 (out of 7 COVID-19 patients). 7/469 patients contracted COVID-19, and 4 died (1 other death occurred which was attributed to disease progression and not COVID-19) | N | NR | NR | Access to anticancer treatment and radiological exams was recorded; A significant reduction in access for therapy was seen when compared with 2019 (2590 versus 2974, access rate ratio (ARR) = 0.85, p < 0.001), no significant differences in access numbers and ARR was evident between 2020 and 2018, 2017, or 2016 (2590 versus 2626 (ARR = 0.07), 2660 (ARR = 0.99), and 2694 (ARR = 0.96), respectively, p > 0.05)  A significant reduction in radiological exams was found in 2020 versus all the other years considered (211 versus 360, 355, 385, 390 for the years 2020, 2019, 2018, 2017, and 2016, respectively, p < 0.001) | Y | Treatment delay - (Secondary objective) 63 patients (63/469 total) had their treatment delayed due to COVID-19-related reasons. The median age of these patients was 70 years old; the majority had metastatic breast cancer (62%) and came from a city outside Pavia and its province (66%)  *Reasons for delay was recorded:* Fear of contracting the infection: (38%) Flu-like syndrome: 18% of patients Quarantine (13%) Deaths (13%) Living in red areas (5%) Displacement problems due to closure of the region and province borders (13%)  In the 2020 pandemic, 32 patients delayed treatment due to toxicity (20 cases) or worsening of clinical conditions (12 cases) compared to 45 patients in 2019 (25 for toxicity and 20 for worsening of clinical conditions), 39 in 2018 (22 for toxicity and 17 for worsening of clinical conditions), 41 (21 for toxicity and 20 for worsening of clinical conditions), and 35 in 2016 (18 for toxicity and 17 for worsening of clinical conditions). Only seven patients delayed/stopped active oncological treatments for ascertained COVID-19. No p-values reported. | NR |
| RashidHons 2020 | In-hospital mortality was higher in the OHCA group during the COVID-19 period compared with pre–COVID-19 (37.7% versus 27.8%; P<0.001). In the multivariable analysis, the adjusted probability of mortality also increased from 27.7% to 35.8% in the COVID-19 cohort compared with 16.9% to 29.8% in the pre–COVID-19 cohort (P<0.001) | Y | The procedural success was similar in both groups, with no difference in the in-hospital mortality, major adverse cerebrovascular events, bleeding, and other periprocedural complications  In hospital bleeding P = 0.34 | N | Acute myocardial infarction hospitalizations during COVID-19 period were reduced by >50% (n=20 310 versus n=9325). OHCA was more prevalent during the COVID-19 period compared with the pre–COVID-19 period (5.6% versus 3.6%), with a 56% increase in the incidence of OHCA (incidence rate ratio, 1.56; 95% CI, 1.39–1.74). Patients experiencing OHCA during COVID-19 period were likely to be older, likely to be women, likely to be of Asian ethnicity, and more likely to present with ST-segment–elevation myocardial infarction. The overall rates of invasive coronary angiography (58.4% versus 71.6%; P<.001) | Y | *Disruptions*: Patients with OHCA during the COVID-19 period experienced an increase in time to reperfusion therapy and slightly less specialist care and use of invasive coronary strategy, whereas the demographics of those selected for PCI seem to have been unchanged. Patients admitted with OHCA during the COVID-19 period were slightly less likely to be seen by a cardiologist - 91.0% versus 96.8%; P<0.001, less likely to be investigated with invasive coronary angiography (58.4% versus 71.6%; P<0.001), and for those with ST-segment–elevation myocardial infarction, had increased time to reperfusion treatment (mean, 2.1 versus 1.1 hour; P=0.05)   Temporal analysis of use of invasive coronary angiography revealed a consistent lower use of an invasive strategy across all months in the COVID-19 period, with almost a 50% reduction in May 2020 compared with May 2019. The use of PCI was also lower across COVID-19 months in 2020 compared with pre–COVID-19 months in 2019.   The study noted a significant reduction in invasive coronary strategy for OHCA in this study, which is associated with improved survival and more favorable neurological outcomes, particularly in those presenting with ST-segment elevation on the ECG. No substantial differences in procedural characteristics and outcomes for patients with OHCA who received PCI during the COVID-19 period were observed | Y |
| Rebecchi 2020 | NA | NR | No significant changes in complications (52.2% in 2020 vs. 51.6% in 2019) p=1.0 | N | NR | N | Elective esophageal cancer surgery  6 (50%) centers - report no change in oncologic surgical practice 3 (25%) declared a reduction in the number of esophageal resections,  2 (16.7%) limited esophageal resections only to selected patients with-out severe comorbidities,  1 (8.3%) completely stopped its surgical activity  Esophageal resection was delayed in 24 (36.9%) patients in 6 (50%) centers (Table 1), due to limited availability of ICU facilities (4 units) and complete lockdown (1 unit).   At the time of completing the questionnaire, 7 (29.1%) patients were still on the waiting list; the delay of surgery in the remaining 17 patients who were operated ranged from 15 to 45 days.   Indications and timing of neoadjuvant treatment were extended in all patients who had their operation postponed. Despite the restrictions related to the epidemic, none of the centers included in the survey opted for a therapeutic shift to definitive chemo (radiation) therapy in patients scheduled for surgery  No significant change in ICU stay (mean [sd]) - 2020 - 2.8 d [2.4], 2019 - 2.3d [1.6] p =0.628 No significant change in hospital stay (mean [sd]) - 2020 - 11.5 d [3], 2019 - 12d [4.4] p =0.767 | Y |
| Richter 2020 | For acute ischemic stroke:  In-hospital mortality (8.1% during pandemic vs. 7.6% pre pandemic) p=0.006 In-hospital mortality (8.1% during pandemic vs. 7.4% 2019) p<0.001  For intracerebral hemorrhage: Increase of the in-hospital mortality in patients with ICH during the pandemic (34.9%) compared with the prepandemic period (29.9%, P<0.001), also increase vs historical control (29.2% p <0.001) | Y | NA | NR | NR | N | Hospital admissions for AIS sharply dropped during the pandemic period (n=31 165) compared with the prepandemic period (n=37 748, −17.4%) and the corresponding time period in 2019 (n=38 247; −18.5%). no p value reported  Decrease of 22.9% in hospitalization of patients with TIA during the COVID-19 pandemic (n=13 015) vs. prepandemic period (n=16 883) and in comparison with the same time period in 2019 (n=17 608; −26.1%). no p value reported  Decrease of 15.8% was also found for hospital admissions of patients with ICH (intracerebral hemorrhage) during the pandemic period (n=3803) compared to the prepandemic period (n=4518) no p value reported | Y |
| Riemann 2020 | NA | NR | NA | N | NR | N | The median number of critical procedures per week fell significantly from 44 before to 24 after March 16, 2020 (8weeks before and 8 weeks after) p = 0.001  Out of the subgroup of critical procedures:   Diagnosis of suspected malignancy from a median number of 21.5 per week to 11.5 p=0.002 (p value in supplementary tables)  No significant difference when analyzing emergency procedure for acute bleeding and acute inflammatory disease (p=0.054), but still have 51% drop  Decrease in procedures for suspected malignancies 8 weeks after vs before March 16 p= 0.002  Tumor operations were not affected significantly 8 weeks before vs after March 16 p=0.43  Procedure for salivary gland masses were not affected significantly 8 weeks before vs. after Mar 16 2020 p=0.05 | Y |
| Rodriguez-Leor 2020 | In-hospital mortality higher during COVID 19 (7.5 % vs 5.1% unadjusted OR 1.50; 95%CI 1.07-2.11; P < .001), adjusting for cofounder[age, sex, Killip class, and time from symptom onset to reperfusion] - (risk adjusted OR 1.88 95%CI, 1.12-3.14 p = 0.017) attenuated after additional adjustment for confirmed COVID-19 diagnosis (OR, 1.56; 95%CI, 0.91-2.67; P = .108).  patients recruited during the COVID-19 outbreak vs those recruited 1 year before: patients with STEMI during the COVID-19 outbreak were at higher risk of in-hospital mortality after adjustment for confounding (P = .033), but this significant association disappeared when COVID-19 status was introduced into the model (P = .203), suggesting that COVID-19 was the driver of the increase in in-hospital mortality between cohorts. | Y | We did not observe an increase in cases of ventricular fibrillation ( 6.2% during covid vs 6.4% p =0.85) or asystole(0.5% vs. 1.1% p=0.092) or in a need for mechanical ventilation (3.7% vs 3.2% p=0.56) prior to the catheterization laboratory in patients with confirmed ST elevated myocardial infarction (STEMI.) | N | NR | N | During COVID-19 there was increase in both time from symptom onset to first medical contact (105 [45-222] vs 71 [30-180] minutes, P < .001) and time from symptom onset to reperfusion (233 [150-375] vs 200 [140-332] minutes, P < .001) Time from cath Lab arrival to reperfusion was 4 minutes longer during pandemic (+20%, 24 min vs. 20 min) p <0.001  First medical contact to cath lab arrival time was not significant (83 [55-125] vs 86 [59-125] minutes, P =0.089)   During COVID-19, patients more frequently arrived at the hospital via the out-of-hospital emergency medical service and, once at the pPCI hospital, were more frequently admitted directly to the catheterization laboratory. p=0.017 | Y |
| Rupa 2020 | For cranial elective procedure March-April 2020 - in house mortality n=1, March-April 2019 - in house mortality n=2 p=1 For spinal elective procedure March-April 2020 - in house mortality n=0, March-April 2019 - in house mortality n=2 p=0.51 | N | The study does include a case study that outline potential deleterious effects of delaying elective surgery. | NR | NR | N | Daily outpatient numbers (ON) and elective procedures (EP) Moving averages of ON (MAON) coronavirus-positive cases in Germany (CPCG) Exponential and arc tangent curves (ATC) of covid cases in Germany [modelling used to obtain an equation] derivative function of the fitted ATC (DFATC). (Rate of corona virus increase)  Elective procedure (include non-oncological) including the subset of non-oncological EP (p = 0.032), were significantly less performed in the first half of 2020 as compared to the first half of 2019 (Jan 1 – June 30) 2019 vs 2020  Correlated the DFATC with the MAON from 1 February 2020 until 31 March 2020, which resulted in a rho value of -0.92 with a p value of less than 0.0001  shifted the DFATC by 10 days and correlated this curve with the MAON from 1 April 2020 until 30 June 2020, which resulted in a rho value of -0.87 with a p value of less than 0.0001  Significant differences were found for the numbers of procedures indicated due to motor deficits (n=15 in 2020 vs. n=5 p=0.0167), visual disturbances (n=8 in 2020 vs n=2, p=0.0488), and spinal instability(n=9 in 2020 vs. n=1, p=0.0012), which were performed more frequently in March and April 2020 as compared to March and April 2019, and for procedures indicated due to radicular pain (n=5 in 2020 vs. n=18 p=0.0489), which were conducted less frequently in March and April 2020 as compared to March and April 2019  Length of stay was examined for specific elective procedure during March and April 2019 vs. March and April 2020 For spinal or cranial elective procedures: significant found: Longer in pandemic period Median = 10 (Range 3-111) vs 8 (Range 2-67 days) p=0.0497  No significance was found when examining spinal (Median = 12 (Range 3-111) vs 9 (Range 3-67 days)p=0.2674) and cranial (Median = 9 (Range 3-77) vs 8 (Range 2-48 days) p=0.0946 elective procedures separately | Y |
| Russo 2020 | NA | NR | NA | NR | NR | N | No significant differences were found for what concerns planned hospitalizations (7.2% vs. 5.6%; P = 0.638) and inpatient stays (3.1% vs. 2.8%; P = 0.903) between the two observation periods | N |
| Salarifar 2020 | All cause mortality at 70 days (n=8, 4.5%) vs (n=4, 2.7%) p=0.4 | N | Single cases of nonfatal myocardial infarction (MI), repeated revascularization with coronary artery bypass grafting, and rehospitalization in the cardiac care unit were reported at a median follow up of 70 days in patients who were admitted in 2019. A single case of nonfatal MI and 2 readmissions to the cardiac care unit occurred at follow- up of the same duration in patients with STEMI during the COVID-19 outbreak. There was no difference between the 2 study groups in terms of 70-day occurrence of nonfatal MACEs (P = 0.8) | N | NR | N | Pandemic data minutes are reported first in brackets  No differences were observed between the 2 study groups in terms of ST-elevation myocardial infarction (STEMI) STEMI-related target times, a trend towards a prolonged time from symptom onset to first medical contact (P = 0.84, median 365.5 min vs 363 min) and shorter time intervals from first medical contact to STEMI diagnosis (P = 0.16 5 min vs 5 min), from STEMI diagnosis to wire crossing (P = 0.95 49.5 min vs 50 min), and from first medical contact to wire crossing (P = 0.12 61min vs 67.5 min) were reported during the COVID- 19 outbreak compared with the same period in the preceding year | N |
| Scholz 2020 | COVID compared to non-COVID in-hospital mortality was not significantly different (9.2% vs. 8.5%, p=0.074) | N | NA | NR | ST elevation myocardial infarction (STEMI) hospital admission rates dropped 12.6% (no p-value given) | NR | (1) Several time-periods from symptoms onset to surgery: Among all the time-points studied, only field-to-hospital time was greater in COVID (no values given, p=0.003) and cath-to-puncture time was longer in COVID (12.9±0.3 min vs. 14.1±0.5 min, p =0.029). These measurements were adjusted for gender, age, thrombolysis in myocardial infarction (TIMI) score, infarct location, and thromolytic therapy using regression models. (2) Pre-hospital electrocardiogram: similar COVID vs. non-COVID (96.7% of patients vs. 96.9% of patients, no p-value given) (3) Direct transfers to percutaneous coronary intervention (PCI) lab from EMS (bypassing emergency room): similar [66.3% vs. 67.8%, p=0.6] (4) TIMI scores after PCI: similar (6% with score 0-2 and 94% with score 3 vs. 6% score 0-2 and 94% score 3, p=0.6) | Y |
| Scortichini 2020 | Excess mortality during COVID (29.5% increase, no p-value given). Excess mortality reaches 400% in some small provinces of Italy (no p-value given)  Relative risk ratios (i.e. risk of mortality) were greatest males compared to females (peaked at ~1.7 vs. ~1.5, respectively; no p-value given), >70yrs age compared to <70yrs age (peaked at ~1.7 vs. ~1.4, respectively; no p-value given), and in Northen Italy compared to Italy as a whole (peaked at ~2.2 vs. ~1.6, respectively; no p-value given). [Values are approximate because estimated from a graph. No precise values were supplied in the article text] | N | NA | NR | NR | N | NA | N |
| Secco 2020 | No significant difference (just stated, no specific values given) | N | Insignificant differences in rates of hypertension, diabetes, dyslipidemia, body mass index>30, coronary artery disease, atrial fibrillation, and chronic kidney disease comorbidities  Significant difference in rates of chronic obstructive pulmonary disease (COPD) comorbidity (COVID-8.3% vs. non-COVID-21.6%, p<0.01) | Y | NR | N | Significant findings (COVID group vs. non-COVID): greater Global Registry of Acute Coronary Events (GRACE) score (126±27 vs. 116±26, p<0.01),  greater ST elevation myocardial infarction (STEMI) with time-to perfusion delay >24hrs (17.8% vs. 4.3%, p<0.01),  lower rates of non-STEMI (NSTEMI) (39.3% vs. 57.4%, p<0.01),  greater rates of other Acute Coronary Syndrome (ACS)' (2.4 vs. 1.9%, p<0.01), longer door-balloon times (66±17 vs. 40±12 min, p<0.01), longer STEMI symptoms to percutaneous coronary intervention (PCI) time (5.8±3.1 vs. 3.9±2.2 hrs, p<0.001),  longer NSTEMI symptoms to percutaneous coronary intervention (PCI) time (36.9±38.4 vs. 18.8±20 hrs, p<0.01), greater basal and peak troponin levels (5138±9408 vs. 1142±4017 and 13681±10936 vs. 9143±13825, respectively, both p<0.01),  greater rates of left ventricular ejection fraction (LVEF)<40% (42.8% vs. 24.7%, p<0.01) | Y |
| Seiffert 2020 | Increased mortality in COVID period for stroke patients only (9.8% vs. 8.5%, p<0.05) | Y | NA | NR | NR | N | Admission rates decreased for ST elevation myocardial elevation (STEMI) (-12.2%), NSTEMI (-15.2%), Acute limb ischemia (-12.4%), stroke (-8.9%) and transient ischemic stroke (-14.6%) (all p<0.05). Admission rates for aortic rupture decreased but was not statistically different (-16.3%) | N |
| Sharma 2020 | NA | NR | Significantly increased National Institutes of Health Stroke Scale in COVID vs. non-COVID group (10 [8-11] vs. 7 [7-10]) [reported as median [range], p<0.01] | Y | NR | N | NA | N |
| Silva 2020 | Significantly increased excess mortality and standardized mortality ratios (SMR) (using the non-COVID period as the standard) in various locations in:  1) Brazil as a whole: Excess mortality- 39,146 deaths over March-May (no p-value). SMR- March was 1.02 (95% confidence interval (CI): 1.01-1.03), April was 1.04 (1.03-1.05), May was 1.14 (1.13-1.15) 2) Individual provinces in Brazil: Excess mortality- ranged from 0-3,142 over March-May (no p-values). SMRs- March ranges from 0.41 (95% CI: 0.3-0.55) to 96.16 (78.15-117.07), April ranges from 0.54 (0.39-0.73) to 2.22 (2.07-2.37), May ranges from 0.62 (0.52-0.74) to 3.16 (3.04-3.29) | Y | NA | NR | NR | N | NA | N |
| Sinnathamby 2020 | Mortality risk hazard ratios (RHR) for different household sizes in pandemic compared to non-pandemic time (all have p<0.001): 0.785 (2-4 person household), 1.465 (5-8 person), and 5.082 (>9 person) | Y | NA | NR | NR | N | NA | N |
| Slullitel 2020 | COVID vs. non-COVID: 10.8% vs. 0% (p=0.002) | Y | COVID compared to non-COVID:  Preoperative Charleson's Comorbidity Index (CCI) scores: moderate: 17.6 vs. 36%; severe: 79.7 vs. 60.5%; p = 0.030 Preoperative instrumental activities of daily living (IADL) scores: 5 [IQR, 3–6] vs. 3 [IQR, 2–4]; p = 0.001 Preoperative Frailty index: 56.8% vs. 37.2%; p = 0.013 Thromboembolic events: 6.75% vs. 0%; p = 0.02 | Y | NR | N | COVID compared to non-COVID:   Median time to surgery: 24 [interquartile range (IQR), 24–48] vs. 16.5 [IQR, 9–30], (p<0.0001)  Post-operative length of stay: 6 [IQR, 5–8] days vs. 5 [IQR, 4–7] days, p = 0.00001 | Y |
| Sobti 2020 | No significant difference  COVID: 8.5%; non-COVID 2020: 8.2% (p=0.64); non-COVID 2019: 4.2% (p=0.15) | N | NR | NR | NR | N | Number of surgeries performed within 24hrs: COVID- 55.5%, non-COVID 2020- 63.4% (p=0.62), non-COVID 2019- 69.8% (p=0.36)  post operative American Society of Anaesthesiologists score = 3 or 4: COVID- 79.8%, non-COVID 2020- 81.7% (p=0.83), non-COVID 2019- 75.5% (p=1.0) | N |
| Stang 2020 | Excess mortality (COVID compared to non-COVID): there was an excess in age groups 60-69 (1,336 deaths), 80-89 (7,287 deaths) , and 90+ (4,167 deaths). There was a deficit in age groups 0-29 (-62 deaths), 30-49 (-313 deaths), 50-59 (-497), 70-79 (-3848 deaths). Net excess accounting for all age groups was 8071 deaths. Raw standardized mortality ratios (SMRs): Net SMR was increased (1.03 [95% CI 1.03-1.04]). Age specific SMRs were most highly increased in age groups 80-89 (1.08) and 90+ (1.09).  SMRs adjusted for population inflation: Net SMR was decreased (0.98 [95% CI 0.98-0.99]). No p-values given | Y | NR | NR | NR | N | NR | N |
| Stohr 2020 | NR | NR | NR | NR | Lockdown period vs. Non-COVID period: significant decrease in hospital admission rates of 'discretionary' diseases (i.e. patient can decide to visit hospital or not) (-23.9%, no p-value given), but no significant change in admission rates of 'unavoidable' diseases (i.e. patient must visit hospital due to severity) (-0.4%, no p-value given). The specific 'discretionary' disease with significant reduction in hospital admission were unstable angina (-23%, p=0.004), HF (-38%, p=0.002), COPD (-28%, p=0.033), and dizziness/syncope (-53%, p<0.001). | N | NR | NR |
| Stokes 2020 | Significant findings (COVID group compared to non-COVID group):  1) Using ordinary least squares regression analysis, the correlation coefficient between COVID-19 deaths and all-cause deaths (β2) is estimated to be 1.2 (95% confidence interval (CI) 1.16-1.24). This suggests that for every 100 COVID-19 attributed deaths, there were 120 total excess mortalities (i.e. 20/120 excess mortality deaths during COVID were not directly caused by COVID illness). | Y | NR | NR | NR | N | NR | N |
| Strang 2020 | NR | NR | NR | NR | NR | N | Significant findings (COVID-19 deaths during pandemic as compared non-COVID-19 deaths pre-pandemic):  1) less proportion of patients retained ability to express their will (65% vs. 68%, p<0.05) 2) less patients had end-of-life (EOL) discussions (74% vs. 79%, p<0.001) 3) less patients died with someone present (59% vs. 83%, p<0.001) 4) less patients died with a relative present (17% vs. 50%, p<0.001)  Insignificant findings:  1) EOL discussion with patient relatives (84% vs. 86%, p -value is 'non-significant') 2) patients dying with staff present (47% vs. 39%, p -value is 'non-significant') 3) follow-up discussions offered to relatives (79% vs. 78%, p -value is 'non-significant') | Y |
| Strang 2020 | March (2020 compared to 2016-2019): 23% excess mortality (p<0.001) April: 113% excess mortality (p<0.001) May: 44% excess mortality (p<0.001) | Y | NR | NR | NR | N | >1 changes in location-of-care during last two weeks of life in nursing home residents (March-May): 15.2% during COVID period (2020) vs. 28.3% during non COVID period (2016-2019) (p<0.001) | Y |
| Strauss 2020 | NA | NR | NA | NR | NR | N | NA | N |
| Tanacan 2020 | NA | NR | NA | NR | Admitted to hospital (Covid compared to non-covid): 717 vs. 1165 (p<0.001)  Number of patients hospitalized: 1.1% vs. 1.7% (p=0.51) | Y | Differences in rates of therapies/interventions (COVID comapred to non-COVID):  Biopsies- 1.8% vs. 5.7% (p<0.001) Electrocautery- 0% vs. 1.4% (p=0.002) Use of biological agents- 0.6% vs. 2.1% (p=0.01) All others had similar rates | Y |
| Teo 2020 | NR | NR | NR | NR | NR | N | Median stroke onset-to-door time (stroke onset to hospital arrival): 154 versus 95 minutes, *P*=0.12).  Proportion of individuals with onset-to-door time within 4.5 hours: 55% versus 72%, *P*=0.024.  Median ambulance scene arrival to hospital time: 26 vs. 24 minutes, P=0.31.  Intravenous thrombolysis: 7% vs. 8%, P=0.95 Median door to needle time: 53 vs. 67 minutes, P=0.25 Mechanical thrombectomy: 4% vs. 7%, P=0.43 Median door to groin puncture: 98 vs. 119 min, P=0.059 Median arrival to operation theatre-to-perfusion time: 82 vs. 88, P=0.39 Cases of transient ischemic attack: 4% versus 16%, *P*=0.016, despite no increase in referrals to the transient ischemic attack clinic (P>0.05). | Y |
| Thakrar 2020 | The 30-day mortality rate was 16.3% vs. 9.8% (p = 0.022) in Control group A, 2.1% (p = 0.003) in Control group B and 4.2%, (p = 0.001) in Control group C. All control groups are in the pre-pandemic period. | Y | NR | NR | NR | N | NR | N |
| Tomasoni 2020 | Inhospital deaths: 4 (12%) vs. 3 (6%), p=0.376 | N | Incidence of the composite end-point, including free-wall rupture, severe left ventricular dysfunction, left ventricular aneurysm, severe mitral regurgitation and pericardial effusion, was higher (19.6 vs 41.2%; P = 0.030; odds ratio = 2.87; 95% confidence interval 1.09–7.58). | Y | Hospitalizations: 51 (60%) vs. 34 (40%), p value not reported | NR | Time between symptoms onset and first medical contact (FMC): 148 (79–781) versus 130 (30–185) min, P = 0.018.  Time between FMC and primary percutaneous coronary intervention (PPCI): 75 (59–148) versus 45 (30–70) min; P < 0.001. | Y |
| Toner 2020 | In-hospital mortality, mean (IQR): 3 (9.4) vs. 13 (5.9) p=0.47 | N | NR | NR | Hospitalizations for heart failure: 32 vs. 54 (41% reduction, range 44% to 74%; p < 0.001) | Y | NA | NR |
| Tousek 2020 | In-hospital mortality rate of non-ST elevation acute coronary syndrome (NSTE-ACS): 3% vs. 3.3% p = 0.960) In-hospital mortality rate of ST-elevation myocardial infarction (STEMI): 11.8% vs. 9.6% p = 0.735 | N | NR | NR | Intensive care unit (ICU) stay for Non-ST elevation acute coronary syndrome (NSTE-ACS) patients: 6.2 ± 6.6 days vs. 4.7 ± 6.2 days (p = 0.024).  ICU stay for ST-elevation myocardial infarction (STEMI) patients: 4.6 ± 5.4 days vs. 4.3 ± 4.3 days (p = 0.751).  Total hospital stay for the NSTE–ACS patients: 11.1 ± 8.0 days vs. 9.4 ± 9.2 (p = 0.016).  Total hospital stay for STEMI patients: 8.5 ± 7.0 days vs. 8.0 ± 5.4 days (p = 0.491). | Y | The time from symptom onset to first medical contact (FMC) and electrocardiogram (ECG) was less than 24 h in 49% of patients vs. 46% of patients (p = 0.588).  Coronary angiography was performed in 99% vs. 99.8% (p value not reported). Of those, procedure was performed within 24 h of admission in 49% of patients vs. 44.5% (p = 0.462). The time from symptom onset to FMC did not differ between patient groups admitted to the hospital within 24 h of symptom onset ((p=0.275) The time from FMC to vessel recanalization did not differ between groups (p = 0.728). | N |
| Trabattoni 2020 | In-hospital mortality rate: 38% vs. 10% | NR | Among the non ST-elevation myocardial infarction (NSTEMI) cases a higher incidence of myocardial infarction with nonobstructive coronary arteries was observed (45% vs. 22%, p value not reported) | Y | 46 admissions (24 STEMI; 22 NSTEMI) vs. 19 (10 STEMI; 9 NSTEMI), accounting for a 2.5-fold increase in ACS cases (monthly rate 46 vs 18 cases), p values not reported.STEMI = ST-elevation myocardial infarction; NSTEMI = non-STEMI, ACS = acute coronary syndrome | NR | Delay ( > 24 hours) in seeking first medical contact after chest pain onset in 41% of ST-elevation myocardial infarction (STEMI) patients (24.8 ± 51 hours) vs. 20% (6.4 ± 6 hours), p value not reported. | NR |
| Uchino 2020 | NA | NR | NA | NR | Median (IQR) reported.  The daily stroke alerts decreased to 8 (4-10) vs. 10 (8–13), p = 0.001 Stroke telemedicine activations decreased to 4 (3-5) vs. 5.5 (4-7.75), p = 0.02 The daily administration of thrombolysis decreased to 0 (0-1) vs. 1 (0-2), p = 0.03) Daily thrombectomies remained unchanged: 0 (0-1) vs. 0 (0-1), p=0.94 | Y | Median (IQR) reported. Time to presentation among stroke alerts, min: 160 (43.5-430.5) vs. 114.5 (50-404), P=0.63).  Stroke severity by National Institutes of Health Stroke Scale (NIHSS) in the overall stroke alerts: 3 (1-9) vs. 3 (1-7), P=0.435)  Door-to-Computed Tomography completion time, min: 22.5 (14-50.75) vs. 27 (15-55), P=0.30 Door-to-needle time, min: 37 (30.75-58) vs. 46 (35.75-59.75), P=0.23 Door-to-needle time <= 45 min, %: 77% vs. 48%, P=0.35 Door-to-puncture time, min: 84 (68-166) vs. 67 (35-116), P=0.067 Door-to-puncture time <= 90 min, %: 53% vs. 66%, P=0.54 | NR |
| Vandoros 2020 | There is an increase in deaths not reported as Covid-19-related in the pandemic vs non-pandemic period [D-I-D coeff: 967.50; 95%CI: 470.55 to 1464.45]. There are an additional average 968 weekly deaths not officially registered as Covid-19 compared to what would have been expected in the absence of the pandemic. | Y | NA | NR | NR | N | NA | N |
| Vanni 2020 | NA | NR | NR | NR | Hospitalizations: 27.6% vs. 19.7% (Pre-Lockdown 2019 group, p = 0.001) and 18.3% (Pre-Lockdown 2020 group, p < 0.001) | Y | Use of ambulance transport to emergency department (ED) increased to 39.6% of cases vs. 32.5% (Pre-Lockdown 2019 group, p = 0.012) and 30.8% (Pre-Lockdown 2020 group, p = 0.002). Use of autonomous transport to ED decreased to 59.1% of cases vs. 65.9% (Pre-Lockdown 2019 group, p = 0.019) and 67% (Pre-Lockdown 2020 group, p = 0.0065).  Discharging of patients to be followed in outpatient visits decreased to 18.2% vs. 25.9% (Pre-Lockdown 2019 group, p = 0.0027) and 25.3% (Pre-Lockdown 2020 group, p = 0.0077). | Y |
| Vestergaard 2020 | Cumulative excess mortality (all ages): 185,287 deaths vs. 55,441 in 2019; 110,483 in 2018; 83,009 in 2017; and 29,849 in 2016.  At the peak level of mortality in week 14, excess mortality (all ages) was 35,802 deaths vs. 16,165, which is the highest excess mortality in any week during the pre-pandemic periods. P values not reported. | NR | NA | NR | NR | N | NA | N |
| Vieira 2020 | Excess of 1,255 deaths, 14% more than expected based on the previous 10 years' average. A 15.2% increase in deaths from natural causes and a 57% reduction in deaths from external causes compared to the previous 6 years' averages. Excess mortality of 1030 deaths in people aged 75+, compared to the daily averages of deaths in this age group, in the last 6 years. P values not reported. | NR | NA | NR | NR | N | NA | N |
| Wang 2020 | Deaths at discharge: 12 (4.7%) vs. 25 (7.8%), p = 0.131 | N | NA | NR | Hospital admissions: 255 vs. 320, p value not reported. | NR | Percentage of patients who received intravenous (IV) thrombolysis increased to 6.3% vs. 11.8% (p = 0.020).  Percentage of patients who received mechanical thrombectomy increased to 19.2% vs. 13.4% (p = 0.060).  Treatment delay in both IV thrombolysis (median 94.5 min vs. 38.5 min) and mechanical thrombectomy (median 244 min vs. 86 min) in COVID-19 patients; P values not reported. | Y |
| Weinberger 2020 | Excess all-cause deaths: 122,300 (95% prediction interval; 116,800-127,000), which is 28% higher than the official tally of 95,235 COVID-19 reported deaths | Y | NA | NR | NR | N | NA | N |
| Westgard 2020 | NA | NR | NA | NR | NR | N | Decline in emergency department (ED) visits: 147 vs. 250 average daily visits; 7.7% (95% CI: 1.1 to 13.7%) weekly decline, a 49.3% decline overall, and a 35.2% (-38.4 to -31.9%) decline with respect to 2019. Decreases in the proportions of patients presenting with syncope (-70.5%), cerebrovascular accidents (-58.3%), abdominal pain (-43.3%), urolithiasis (-70.0%), and back pain (-50.7%). Increases in the proportions of patients presenting with upper respiratory infections (-10.0%), shortness of breath (25.1%), and chest pain (-13.1%). | Y |
| Wong 2020 | NA | NR | NA | NR | Inpatient hospitalizations decreased by 41.2% (mean ± SD 1,390.8 ± 53.3 vs. 2,364.8 ± 243.1 per week; p < 0.001).  Elective admissions decreased by 58.9% (mean ± SD 377.6 ± 59.4 vs. 918.1 ± 167.1 per week; p < 0.001).  Emergency admissions decreased by 30.0% (mean ± SD 1,013.1 ± 90.4 vs. 1,446.6 ± 119.4 per week; p < 0.001).  Pediatric and adolescent admissions (< 18 years old) decreased by 59.1% (mean ± SD 63.1 ± 8.8 vs. 154.1 ± 28.5 per week, p < 0.001).  Geriatric admissions (>= 65 years old) decreased by 36.7% (mean ± SD 719.6 ± 29.6 vs. 1136.7 ± 118.1 per week, p < 0.001). | Y | Total number of orthopedic operations decreased by 44.2% (mean ± SD 443.6 ± 25.8 vs. 795 ± 115.1 per week; p < 0.001).  Elective operations decreased by 73.5% (mean ± SD 92.8 ± 30.6 vs. 350.1 ± 80.8 per week; p < 0.001).  Emergency operations decreased by 21.2% (mean ± SD 350.8 ± 27.1 vs. 444.9 ± 47.8 per week).  Numbers of operatively treated upper and lower-limb fractures decreased by 23% (75.9 ± 15.2 vs. 98.5 ± 14, p < 0.001) and 20% (168.4 ± 16.9 vs. 210.6 ± 29.5, p < 0.001), respectively.  The number of operatively treated hip fractures decreased by 21.2% (mean ± SD 113.3 ± 17.2 vs. 143.7 ± 21.9 per week, p < 0.001).  Operations for malignant bone and soft-tissue lesions decreased to 3.2 ± 2.1 vs. 5.3 ± 2.5 per week (p = 0.03), which was not significant after Bonferroni correction. Total knee replacements decreased by 80.0% (mean ± SD 11.4 ± 12.9 vs. 57 ± 16.4, p < 0.001).  Total hip replacements decreased by 74.0% (mean ± SD 2.6 ± 2.4 vs. 10 ± 3, p < 0.001).  Anterior cruciate ligament reconstructions decreased by 83.9% (mean ± SD 2.4 ± 2.2 vs. 14.9 ± 4.6, p < 0.001). | Y |
| Woolf 2020 | Excess deaths of 87,001 (95% CI, 86 578-87 423), of which 56,246 (65%) were attributed to COVID-19. | Y | NA | NR | NR | N | NA | N |
| Yalamanchi 2020 | The cardiac intensive care unit (CICU) mortality rate: 4.6% compared to 3.9% in 2018 (p = 0.83) and 5.6% in 2019 (p = 0.70). The in-hospital mortality was also similar in all 3 years (6.5%, 7.8%, and 7.9% in 2018, 2019, and 2020, respectively, p = 0.61). | N | NA | NR | Cardiac intensive care unit (CICU) admissions: 216 vs. 322 in 2019 (33% decline) and 307 in 2018 (30% decline). The decline in admissions with the primary diagnosis of acute coronary syndrome (ACS), acute decompensated heart failure (ADHF), arrhythmia, and other diagnoses were 27%, 38%, 62%, and 59%, respectively, while there was a 50% increase in acute pulmonary embolism (PE) admission compared to the mean admission in 2018 and 2019. P values not reported. | NR | NA | NR |
| Zhang 2020 | Deaths: 4 (3.36%) vs. 2 (1.65%) in 2019 and 4 (2.58%) in 2018. The mortality rates of the patients who did not receive reperfusion was 3.1% in 2020, which is higher than 2.1% in 2019 and 2.9% in 2018. The peak mortality was in February in 2020, 7.6%. P values not reported. | N | NA | NR | Hospitalizations (n): 119 vs. 121 in 2019 and 155 in 2018. P value not reported. | NR | % of no reperfusion patients increased: 52.62% vs. 38.84% in 2019 and 44.52% in 2018.  % of patients who received thrombolysis increased: 18.49% 5.78% in 2019 and 10.97% in 2018.  For patients who received primary percutaneous coronary intervention (PPCI), the % of direct to cardiac catheterization laboratory decreased: 6.25% vs. 17.91% in 2019 and 40.58% in 2018.  PPCI% decreased: 26.89% vs. 55.37% in 2019 and 44.52% in 2018.  Door to device time (min) increased: 72 (66, 87) vs. 57 (47, 71) in 2019 and 55 (33, 71) in 2018.  P values not reported. | Y |

**CI:** confidence interval, **COPES**: Coronavirus Disease (COVID-19) and Outcomes Associated with Pandemic Effects Study (COPES), **COVID-19**: Coronavirus Disease-2019, **mRS:** modified Rankin score, **N:** no, **NA:** not applicable, **NR**: not reported, **NSTEMI**: non-STEMI **p:** p-value, **STEMI**: ST-segment elevation myocardial infarction, **Y**: Yes
