# Supplementary material for: Non-COVID outcomes associated with the coronavirus disease-2019 (COVID-19) pandemic effects study (COPES): A systematic review and meta-analysis
Source: PLoS One. 2022 Jun 24;17(6):e0269871. doi: 10.1371/journal.pone.0269871 (PMC9231780; doi:10.1371/journal.pone.0269871)
Supplement: S7 Table — (DOCX) [file pone.0269871.s008.docx]

**S7 Table: Summary statistics of statistically significant outcomes** for COPES Non-COVID Illness during COVID pandemic**

| **Changes in mortality (statistically significant)** | **n (%)** |
| --- | --- |
| Yes | 50/167 (30%) |
| - Increase in mortality | **49/50 (98%)** |
| - Decrease in mortality | 1/50 (2%) |
| No | 47/167 (28%) |
| Not reported | 71/167 (42%) |
|  |  |
| **Changes in morbidity (statistically significant)** |  |
| Yes | 28/167 (17%) |
| - Increase in morbidity | **26/28 (93%)** |
| - Decrease in morbidity | 2/28 (7%) |
| No | 30/167 (18%) |
| Not reported | 110/167 (66%) |
|  |  |
| **Changes in acute care hospitalizations/occupancy (statistically significant)** |  |
| Yes | 39/167 (23%) |
| - Increase in hospitalizations/occupancy | 1/39 (3%) |
| - Decrease in hospitalizations/occupancy | **38/39 (97%)** |
| No | 111/167 (66%) |
| Not reported | 18/167 (10%) |
|  |  |
| **Disruptions to care (statistically significant)** |  |
| Yes | 63/167 (38%) |
| - Increase in disruptions to care | **47/62 (76%)** |
| - Decrease in disruptions to care | 15/63 (24%) |
| No | 62/167 (37%) |
| Not reported | 43/167 (26%) |

**COPES**: Coronavirus Disease (COVID-19) and Outcomes Associated with Pandemic Effects Study (COPES), **COVID-19**: Coronavirus Disease-2019; **CI**: confidence interval

****p-values < 0.05 or 95% CI does not cross 1**
