# Supplementary figures and images for: Non-COVID outcomes associated with the coronavirus disease-2019 (COVID-19) pandemic effects study (COPES): A systematic review and meta-analysis
Source: PLoS One. 2022 Jun 24;17(6):e0269871. doi: 10.1371/journal.pone.0269871 (PMC9231780; doi:10.1371/journal.pone.0269871)

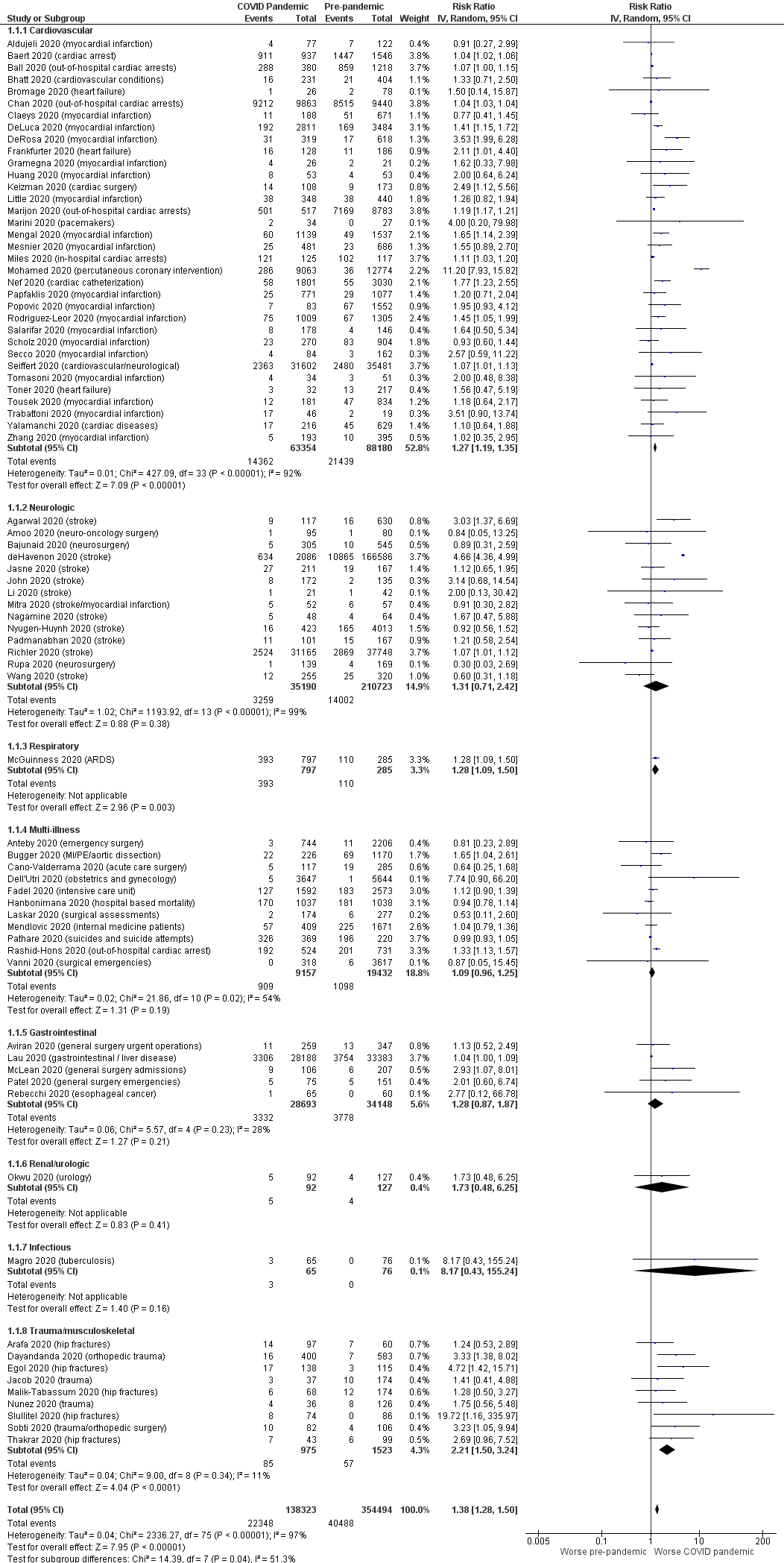

Supplement: S1 Fig — (TIF) [file pone.0269871.s009.tif]

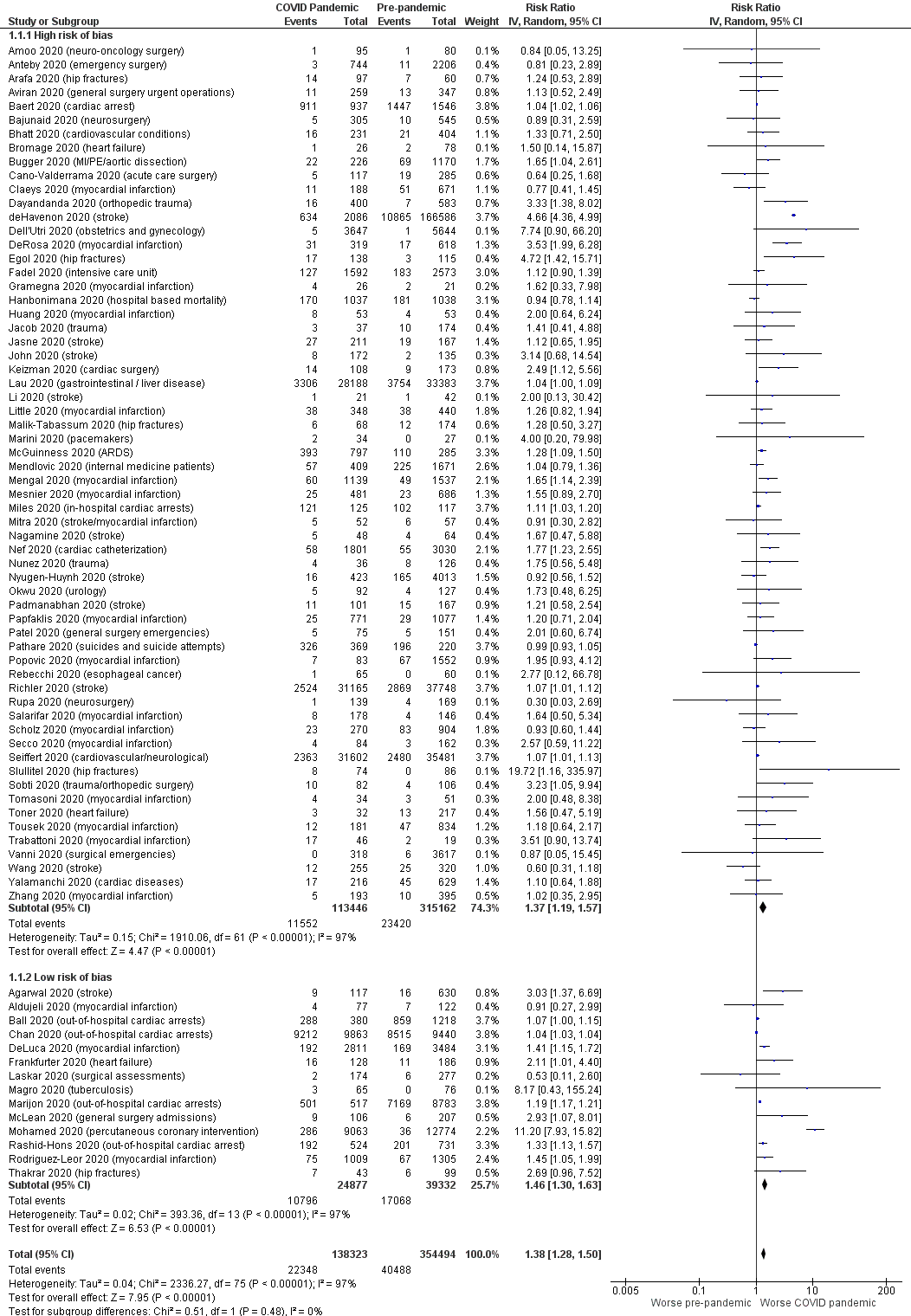

Supplement: S2 Fig — (TIF) [file pone.0269871.s010.tif]

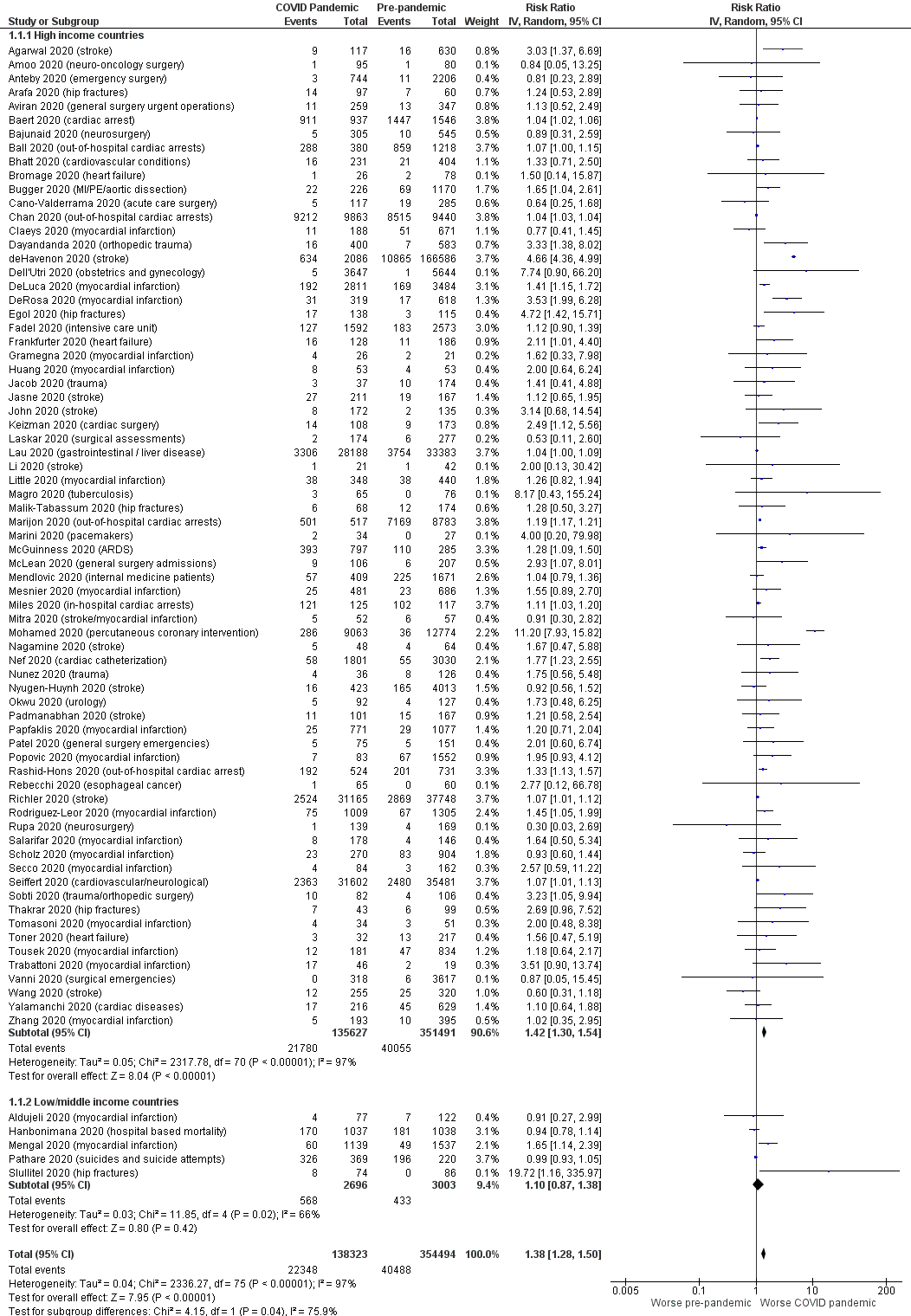

Supplement: S3 Fig — (TIF) [file pone.0269871.s011.tif]

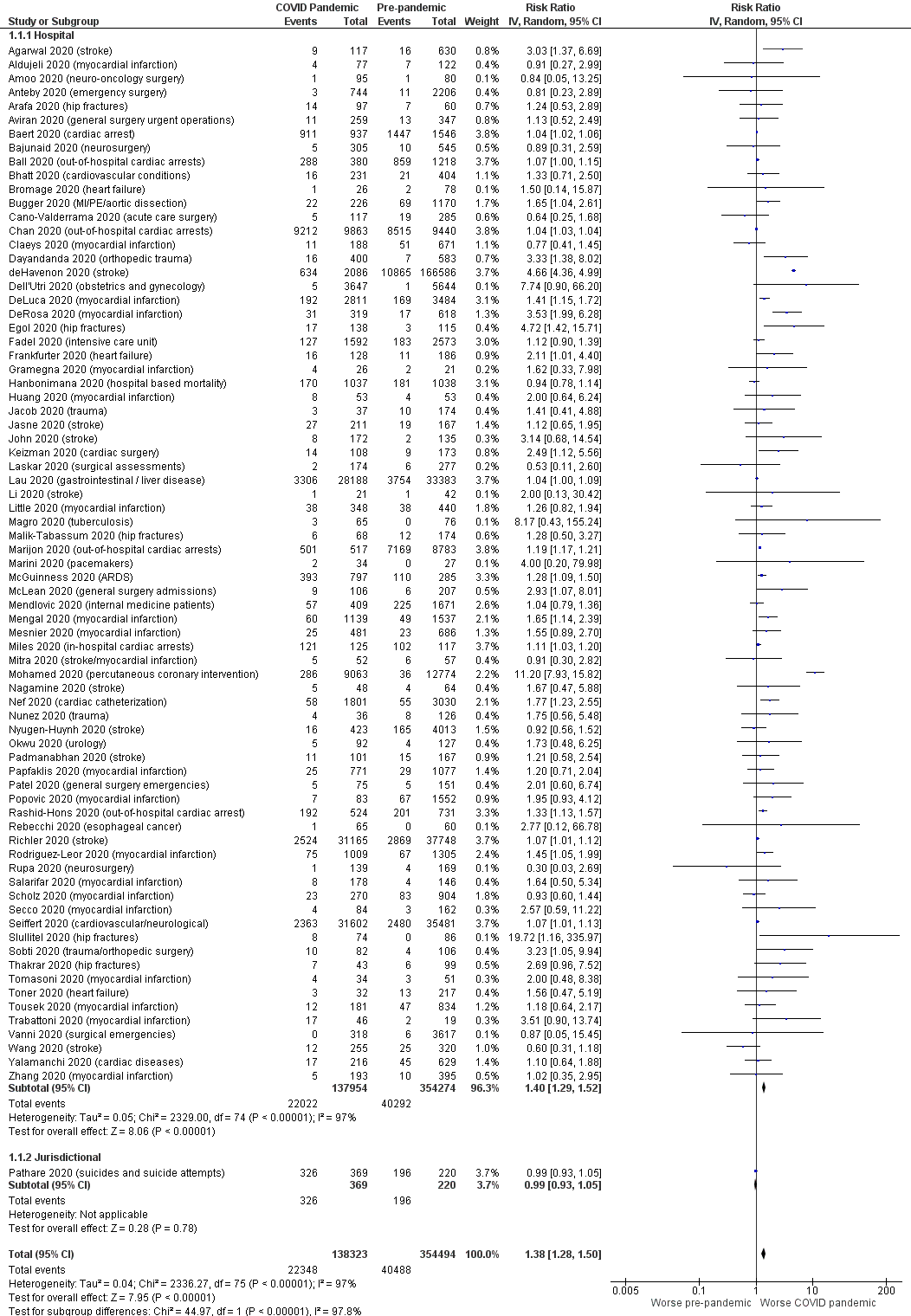

Supplement: S4 Fig — (TIF) [file pone.0269871.s012.tif]

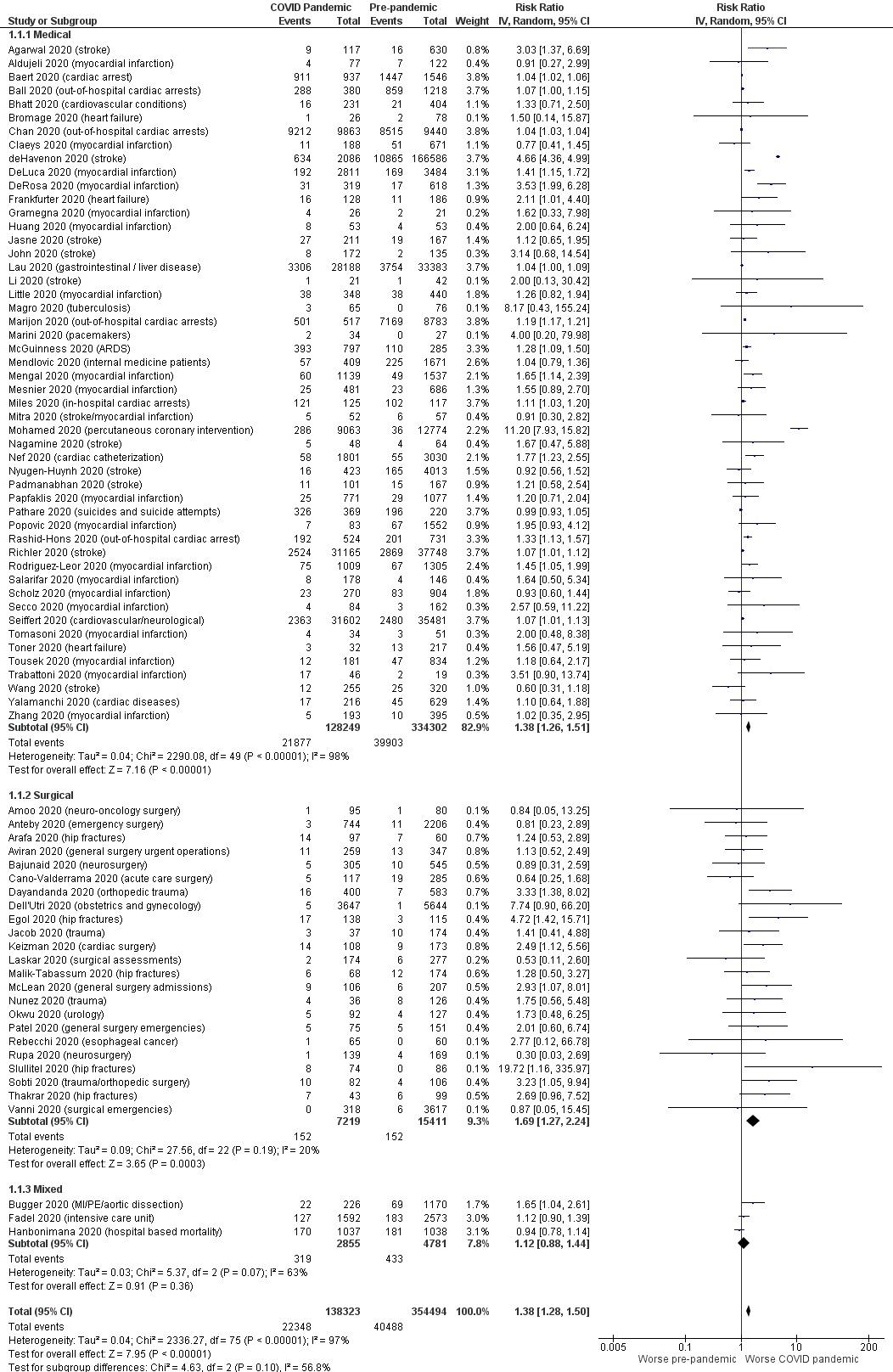

Supplement: S5 Fig — (TIF) [file pone.0269871.s013.tif]

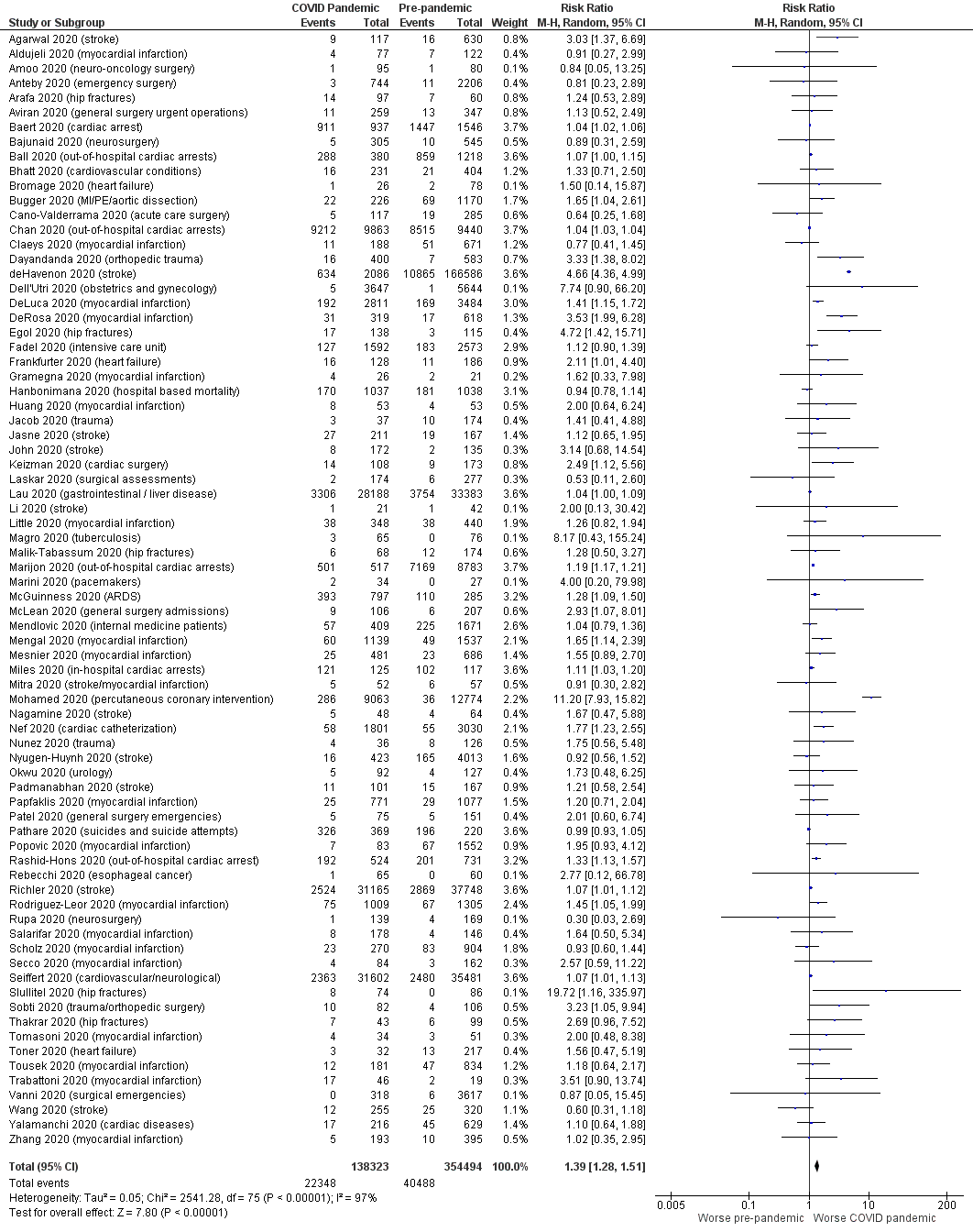

Supplement: S6 Fig — (TIF) [file pone.0269871.s014.tif]

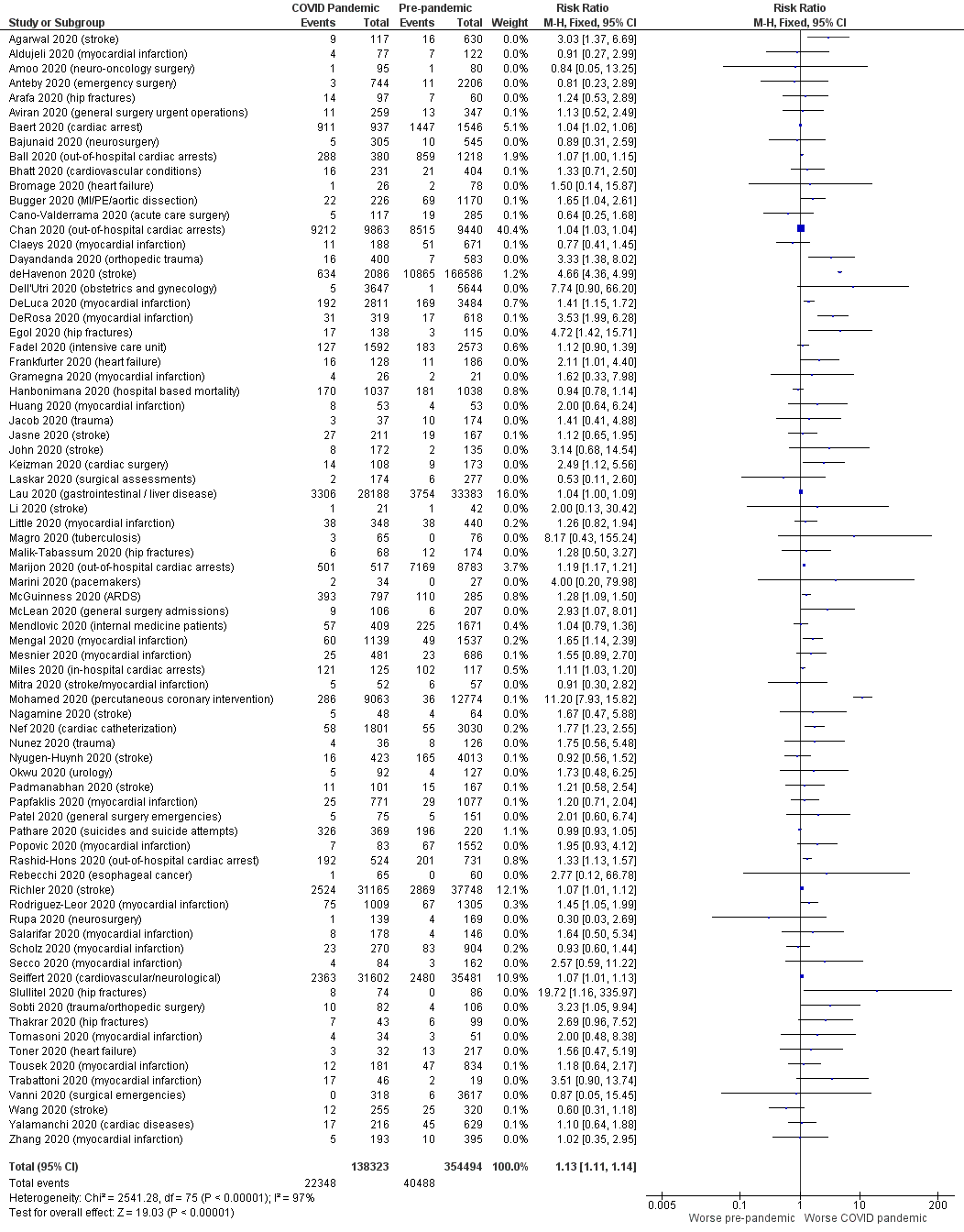

Supplement: S7 Fig — (TIF) [file pone.0269871.s015.tif]

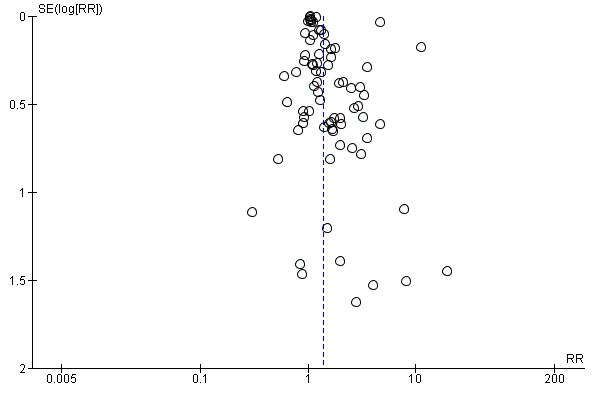

Supplement: S8 Fig — (TIF) [file pone.0269871.s016.tif]
